# Efficacy and safety of small extracellular vesicle interventions in wound healing and skin regeneration: A systematic review and meta-analysis of animal studies

**DOI:** 10.7150/thno.73436

**Published:** 2022-09-06

**Authors:** Maimonah Eissa Al-Masawa, Mohammed Abdullah Alshawsh, Chiew Yong Ng, Angela Min Hwei Ng, Jhi Biau Foo, Ubashini Vijakumaran, Revatyambigai Subramaniam, Nur Azurah Abdul Ghani, Kenneth Whitaker Witwer, Jia Xian Law

**Affiliations:** 1 Centre for Tissue Engineering and Regenerative Medicine, Faculty of Medicine, Universiti Kebangsaan Malaysia Medical Centre, Jalan Yaacob Latif, 56000, Kuala Lumpur, Malaysia.; 2 Department of Pharmacology, Faculty of Medicine, Universiti Malaya, 50603, Kuala Lumpur, Malaysia.; 3 School of Pharmacy, Faculty of Health and Medical Sciences, Taylor's University, 47500, Subang Jaya, Selangor, Malaysia.; 4 Department of Obstetrics and Gynaecology, Universiti Kebangsaan Malaysia Medical Centre, Jalan Yaacob Latif, 56000, Cheras, Kuala Lumpur, Malaysia.; 5 Department of Molecular and Comparative Pathobiology, The Johns Hopkins University School of Medicine, Baltimore, MD, USA; Department of Neurology and Neurosurgery, The Johns Hopkins University School of Medicine, Baltimore, MD, USA; Richman Family Precision Medicine Center of Excellence in Alzheimer's Disease, The Johns Hopkins University School of Medicine, Baltimore, MD, USA.

**Keywords:** extracellular vesicle, exosome, wound healing, skin regeneration, animal models

## Abstract

Small extracellular vesicles (sEVs) have been proposed as a possible solution to the current lack of therapeutic interventions for endogenous skin regeneration. We conducted a systematic review of the available evidence to assess sEV therapeutic efficacy and safety in wound healing and skin regeneration in animal models. 68 studies were identified in Web of Science, Scopus, and PubMed that satisfied a set of prespecified inclusion criteria. We critically analyzed the quality of studies that satisfied our inclusion criteria, with an emphasis on methodology, reporting, and adherence to relevant guidelines (including MISEV2018 and ISCT criteria). Overall, our systematic review and meta-analysis indicated that sEV interventions promoted skin regeneration in diabetic and non-diabetic animal models and influenced various facets of the healing process regardless of cell source, production protocol and disease model. The EV source, isolation methods, dosing regimen, and wound size varied among the studies. Modification of sEVs was achieved mainly by manipulating source cells via preconditioning, nanoparticle loading, genetic manipulation, and biomaterial incorporation to enhance sEV therapeutic potential. Evaluation of potential adverse effects received only minimal attention, although none of the studies reported harmful events. Risk of bias as assessed by the SYRCLE's ROB tool was uncertain for most studies due to insufficient reporting, and adherence to guidelines was limited. In summary, sEV therapy has enormous potential for wound healing and skin regeneration. However, reproducibility and comprehensive evaluation of evidence are challenged by a general lack of transparency in reporting and adherence to guidelines. Methodological rigor, standardization, and risk analysis at all stages of research are needed to promote translation to clinical practice.

## Introduction

Poor skin healing continues to have a substantial impact on the quality of life of millions of individuals around the globe. Skin is the body's first line of defense. In response to injury, skin activates a series of intricately orchestrated events controlled by numerous signals 1, with the goal of restoring the multi-layered structure and the continuum of the skin and reinstating its protective, thermogenic, endocrine and sensory functions 2. Generally, wounds heal through four distinct but overlapping phases. These phases are: 1) hemostasis (platelet aggregation and fibrin clot formation); 2) inflammation (recruitment of inflammatory cells); 3) tissue regeneration (restoration of skin structure via cell proliferation, extracellular matrix deposition, new blood vessel, and appendage formation, resulting in granulation and re-epithelialization); and 4) remodeling (long-term maturation of the newly formed tissue to closely resemble the native equivalent) 2-4. Disruption of any of these phases—due to systemic or local causes—may result in a prolonged healing process or suboptimal recovery, marked by a failure to restore the architecture and function of the healing tissue 5. Due to population aging and comorbidities, the prevalence of chronic non-healing wounds has risen dramatically, affecting millions of individuals each year. This imposes an increasing burden on health systems and economies 5. Acute wounds are also widespread, accounting for millions of medical treatment facility visits and hospital admissions annually. Deep wounds can result in permanent disability and scarring, while burn injuries require lengthy hospitalization, incur high costs, and have high morbidity and fatality rates 6. Unfortunately, currently available remedies for skin wound healing are incapable of meeting the urgent clinical needs 3, 7. Even though standard therapies such as routine debridement, infection management, and dressings may demonstrate some benefits, they fall short of addressing the pathophysiology of dysfunctional healing. Hence, researchers have placed great emphasis on developing biologically active formulations to rescue inadequate repair 3. Of these, single bioactive factors that target specific wound indications—such as cytokines 8 and growth factors 9—have garnered research interest, with a few gaining regulatory approval 3. However, therapeutic modalities that target multiple inherent deficits in non-healing lesions might be more effective in addressing their complex pathophysiology that may include vascular, neurologic, inflammatory, and metabolic impairments 10.

Extracellular vesicles (EVs), which transfer cocktails of functional cargo (such as proteins, lipids, miRNAs, other RNAs, and DNA) horizontally between cells 11, 12 may be multipotent stimulants of endogenous tissue repair 13. EVs are a class of natural anuclear cell-released particles delimited by a phospholipid bilayer. As colloid members of the cell secretome 14, EVs are produced by almost all types of cells, in varying sizes and with different subcellular origins 15. Each EV displays surface molecules that may target recipient cells. EVs are believed to communicate signals by fusing with target cells or simply binding to cell receptors 16, ultimately causing recipient cells to undergo phenotypic changes 12. EVs can interact with target cells residing in the microenvironment or be carried to distant cells via biological fluids, and their internal and external cargo contribute to intercellular communication 17. Recent studies have recognized the role of EVs in the pathogenesis of diseases 18 and in various natural physiological processes 19. Indeed, the potent effects that were once credited to stem cells, for instance, are now thought to be partially mediated by EVs 20, making EVs a promising alternative to potentially risky cell therapies 21. Moreover, EVs from certain sources may benefit from relative immunological tolerance in cross-species and interindividual transfer 22. In the absence of functional definitions, EVs were classically categorized according to combinations of size, biogenesis, and biophysical separation process. For example, as microvesicles (100-1000nm, budding from the plasma membrane, also called ectosomes); apoptotic bodies (1-5μm, released from fragmented apoptotic cells) and exosomes (30-150nm, endosomal multivesicular body-derived nanovesicles) 23, 24. However, due to the increasingly recognized overlap in size between these categories 25 and the absence of universal differentiating markers, the term EVs is preferred 14, 26. This systematic review will focus on the therapeutic applications of a nanosized subclass termed small EVs (sEVs, ~30-200 nm), which includes but is not limited to endosome-origin exosomes. sEVs have been demonstrated to enhance tissue regeneration 27 and to modulate the immune system 28. They have also been used for drug delivery 29, 30, as vaccines 31 as biomarkers 32, and as therapeutic targets in “vesicle-mediated pathogenesis” 33. EVs mediate signaling in all phases of physiological cutaneous wound healing (reviewed extensively in 34). Platelet-[35] and monocyte-derived EVs 36 regulate clot formation and thus hemostasis. Neutrophil-derived EVs modulate inflammation 37. Macrophage-[38] and endothelial progenitor cell-derived EVs 38 drive angiogenesis, and myofibroblast-derived EVs remodel the extracellular matrix (ECM) 39. In recent years, the number of studies examining the therapeutic potential of sEVs in wound healing and skin regeneration has expanded dramatically.

The rapid progression of sEV therapeutic modalities toward clinical applications prompted us to critically appraise the available preclinical evidence for the benefits and adverse effects of sEVs in skin healing and regeneration. In our approach, we emphasized methodological rigor and reporting quality in accordance with field guidelines, including the Minimal Information for Studies of Extracellular Vesicles 2018 (MISEV2018) 14 and the criteria for MSC identification of the International Society for Cell and Gene Therapy (ISCT) 40. We used a systematic review methodology for inclusive, bias-free coverage of existing studies, which could not be achieved by a conventional narrative review approach 41. We further performed a meta-analysis for a quantitative pooled estimate of sEV efficacy across a vast body of literature, while assessing the heterogeneity of study outcomes and the likelihood of publication bias. Our work thus informs the scientific community of the available evidence from preclinical animal research and provides insights into the likelihood of clinical translation.

## Results

### Search results

**Figure 1:**
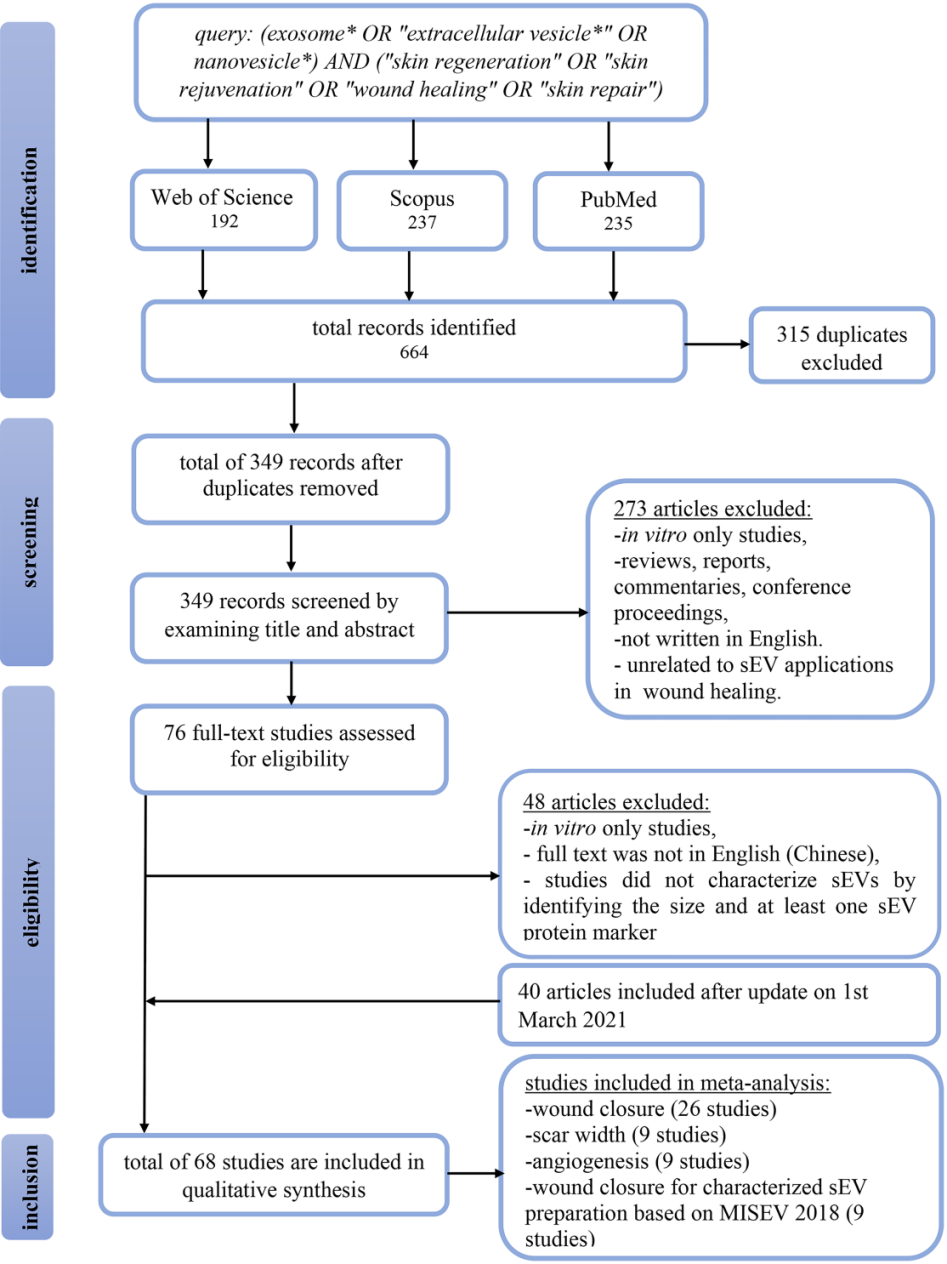
PRISMA flow chart summarizing study screening and selection procedure. Web of Science, PubMed and Scopus were searched for relevant articles from inception to March 1^st^, 2021.

This systematic review was reported according to the Preferred Reporting Items for Systematic Reviews and Meta-Analysis (PRISMA) guidelines. On November 11th, 2019, a search on Web of Science, Scopus, and PubMed retrieved a total of 664 articles. All articles were pooled into Endnote X9.3.3 software, and 315 duplicates were removed. Titles and abstracts were screened to include articles investigating the therapeutic application of sEVs in skin repair, rejuvenation, and wound healing in mammalian models. We excluded 273 studies that were *in vitro* studies, reviews, reports, commentaries, conference proceedings, or articles written in languages other than English. The remaining 76 articles were read in full to determine satisfaction of the eligibility criteria. As a result, 48 studies were excluded, of which two studies were not in English (Chinese), 20 studies did not characterize sEVs by size and/or at least one sEV protein marker, and 26 studies exclusively reporting *in vitro* findings. Additionally, on March 1st, 2021, we updated our search to include another 40 studies, bringing the total number of manuscripts eligible for this systematic review to 68. The flow chart in Figure 1 summarizes the study selection approach.

### General characteristics of the included studies

**Figure 2:**
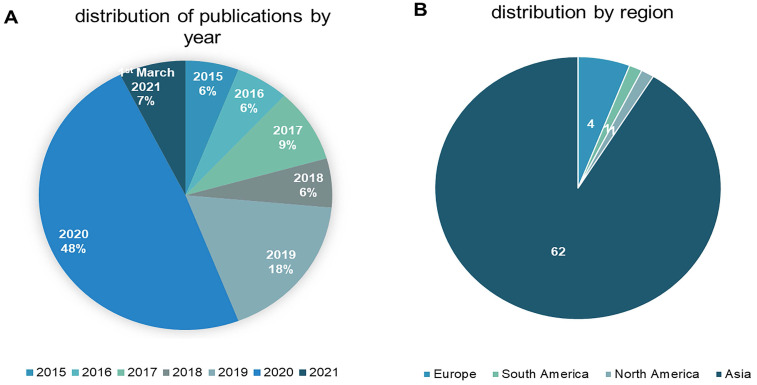
The distribution of the reviewed studies by year (2A) and region according to the corresponding author's affiliation (2B).

The 68 studies identified as eligible for inclusion were published between 2015 and March 1^st^, 2021. Approximately 56% (n = 38) were published in 2020 or later, reflecting a surge in interest in sEVs to promote wound healing and skin regeneration. The studies originated from nine different countries, with China accounting for 84% (n = 57). Figure 2 depicts year of publication (2a) and region according to the corresponding author's affiliation (2b).

### Characteristics of wound healing animal models

#### Animal species

**Figure 3:**
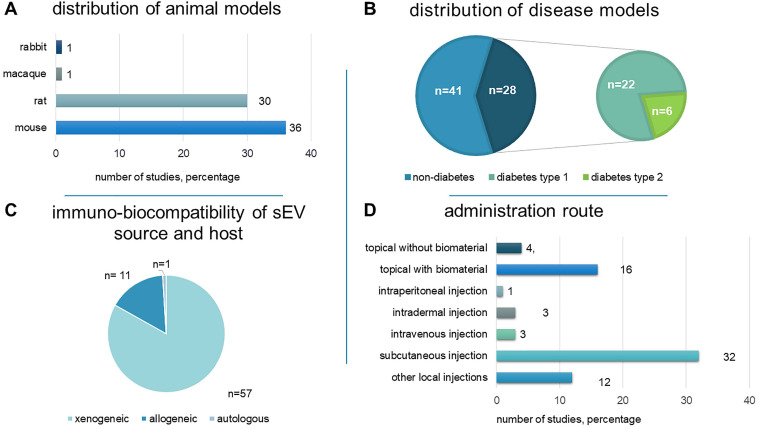
An overview of study characteristics, including distribution of **A)** animal models **B)** disease models **C)** immuno-biocompatibility of the sEV source and host and D) administration route.

Animal models have been used to reveal the intricate physiological and biochemical processes involved in wound healing and skin regeneration, as well as to assess the efficacy and safety of proposed therapeutic interventions. Rodents were used in 66 studies (97%): mice (36 studies) and rats (30 studies). One study tested a non-human primate model (macaque) 42, while another used the New Zealand rabbit model 43 (Figure 3A).

#### Disease models

Non-diabetic wounds and diabetic wounds were investigated in 41 (60.3%) and 28 (41.2%) studies, respectively. One study examined both wound models 44. 22 used streptozotocin (STZ)-induced diabetic rats (n = 15) or mice (n = 7) as a type 1 diabetes model. Six studies utilized genetically modified diabetic db/db mice to represent type 2 diabetes (Figure 3B).

#### Wound models

Full-thickness excisional wounds were the most-studied models (n = 63, 92.6%), of which 58 were “dorsal”, three were diabetic foot ulcer (DFU), one was leg, and one was ear excisional wounds. Other models (n = 6, 8.8%) included burns (n = 3) 45-47, photoaging (n = 1) 48, pressure ulcer (n = 1) 49, and excisional ischemic wounds (n = 1) 50. Wound size ranged in diameter from 4 to 20 mm.

### Intervention characteristics

#### Cellular origin of sEVs

**Figure 4:**
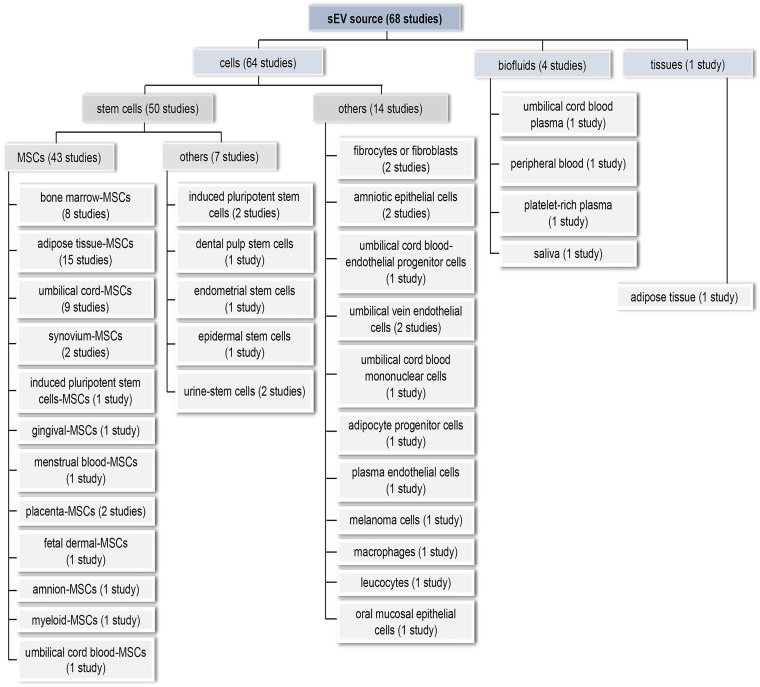
Sources of sEVs used to promote wound healing and skin regeneration. sEVs were isolated from cells, biofluids and tissues.

For comprehensiveness, all sEV source types were included, resulting in a diverse array of sources (Figure 4). In 64 studies (94.1%), sEVs were prepared from a single cultured cell type. We broadly categorised these into MSCs (n = 43, 63.2%), other stem cells (n = 7, 10.3%), and non-stem cell sources (n = 14, 20.6%). Only eight studies (11.8%) used immortalized cell lines. Adipose tissue-derived sEVs were examined in a single study 51. Biofluids—peripheral blood, cord blood, platelet-rich plasma (PRP), and saliva—were the EV source in the remaining investigations, representing a more heterogeneous source of sEVs (n = 4, 5.9%). 58 studies (85.3%) used sEVs from human sources, while ten (14.7%) used non-human sources, *i.e*., rodent (n = 8), pig (n = 1), and macaque (n = 1).

#### Modification of sEVs

##### Preconditioning

Nine studies (13.2%) exposed sEV-producing cells to preconditioning regimens prior to sEV separation. Parent cells were primed with growth factors such as PDGF-BB, TGFβ1, and FGF2 52; enzymes such as thrombin 53; and stressors such as the pro-inflammatory agent lipopolysaccharide (LPS) 50, 53, hypoxia 44, 53, hypoxia mimetic agent deferoxamine (DFO) 54, and hydrogen peroxide (H_2_O_2_) 53. Priming was also done with hormones like melatonin 55 and parathyroid-hormone related peptide (PTHrP-2) 56 or with pharmacological drugs such as Atorvastatin (ATV), an HMG-CoA reductase inhibitor 57. A single study preconditioned source cells with neonatal and adult serum-derived sEVs 58.

##### Genetic modification

Genetic modification of sEV-producing cells was performed in 11 studies (16.2%) to enhance endogenous loading of sEVs with active ingredients such as nucleic acids and proteins/peptides and thus potentiate sEV efficacy. Nucleic acids were introduced by transduction with lentiviruses 47, 59-62; transfection of plasmids 63 or miRNA mimic sequences 64; or electroporation of miRNA sequences 65. Specific noncoding RNAs included 1) miRNAs (miR-126-3p 60, 62, miR-135a 66, miR-21-5p 65, and miR-126 64); 2) long non-coding RNA (lncRNA H19 67), and 3) circular RNA (mmu_circ_0000250 68). Overexpressed specific proteins included the transcription factor nuclear factor-E2-related factor (Nrf-2) 63, tumor necrosis factor (TNF)-stimulated gene-6 (TSG-6) 59, angiopoietin-2 (Ang-2) 47, and PD-L1 61.

##### Loading sEVs with nanoparticles

Two studies loaded superparamagnetic iron oxide nanoparticles (Fe_3_O_4_-NPs) into sEVs by incubating the nanoparticles with the parent cells before sEV isolation 46, 69. Following intravenous administration of nanoparticle-loaded sEVs, Li *et al*. magnetized the nanoparticles using an external magnetic guide positioned beneath the wound site to improve targeting and distribution capabilities 46. In another study, Wu *et al*. applied static magnetic fields (SMF) to parent cells to enhance the therapeutic properties of the secreted nanoparticle-loaded sEVs 69. In that experiment, nanoparticle-loaded sEVs were introduced locally to the wound via subcutaneous injection.

##### Loading sEVs into biomaterial scaffolds

20 studies (29.4%) loaded sEVs into biomaterial scaffolds. Hydrogels were the most preferred choice (n = 18). The remaining studies used polyvinyl alcohol (PVA) (n = 1) 51 and human acellular amniotic membrane (HAAM) (n = 1) 49. Seven studies used synthetic hydrogels: Pluronic F-127 based (n = 4) 61, 70-72, peptide nanofiber (HydroMatrix, n = 2) 73, 74 and gelatin methacryloyl (GelMA) hydrogel 75. Eleven studies used natural hydrogels: chitosan-based (n = 5), plain (n = 1) 62 or incorporated with silk (n = 1) 76, hydroxyapatite (n = 1) 60, glycerol (n = 1) 77, or methylcellulose (n = 1) 78. Alginate-based hydrogels were investigated in three studies 79-81. Hydrogels were usually pre-mixed with sEVs prior to application. Injectable hydrogel formulations were introduced to wound beds in seven studies 44, 70-74, 78.

#### sEV preparation

##### sEV collection conditions

62 studies (91.2%) separated sEVs from conditioned medium. Since serum contains sEVs, 22 studies (32.4%) collected sEVs from serum-free medium. Others prepared culture medium with sEV-depleted FBS (n = 22, 32.4%) or platelet lysate (n = 1, 1.5%). However, only six studies revealed the details of FBS-EV depletion protocols, and without reporting before-and-after particle counts. Chemically defined serum replacements were used in nine studies (13.2%), while autologous serum was the supplement of choice in a single study 82. 11 studies (16.2%) did not report how they dealt with the issue of contaminating sera. 15 studies (22.1%) did not disclose the duration of cell culture conditioning before harvest. In the remaining studies, sEVs were collected after 24 hours (10.3%, n = 7, 11%) or 48 hours (n = 33, 48.5%) of conditioning.

##### sEV separation techniques

**Figure 5:**
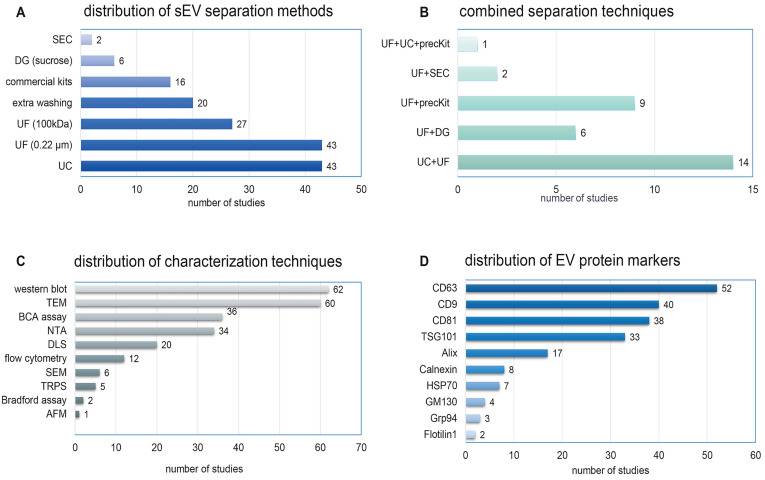
Distribution of **A)** separation methods, **B)** combined separation techniques, **C)** characterization techniques, and **D)** protein markers of sEVs across the 68 reviewed studies. AFM: atomic force microscope; DG: density gradient ultracentrifugation; DLS: dynamic light scattering; NTA: nanoparticle tracking analysis, precKit: precipitation-based isolation kits; SEC: size exclusion chromatography; SEM: scanning electron microscope, TRPS: tunable resistive pulse sensing; TEM: transmission electron microscope; UF: ultrafiltration; UC: ultracentrifugation.

There is no gold standard separation technique for sEVs, and sEV separation methods varied considerably across the studies (Figure 5A). Ultracentrifugation (n = 43, 63.2%) was the most widely used technique, but with various centrifugation protocols. Ultrafiltration by membranes of pore size 0.22 µm (n = 43, 63.2%) or 100 kDa (n = 27, 39.7%) was often done as an adjunct to other separation steps. Commercial precipitation-based isolation kits, density gradient ultracentrifugation, and size exclusion chromatography (SEC) were used in 16 (23.5%), six (8.8%), and two (2.9%) studies, respectively. Additional washing steps were reported in 20 studies (29.4%). No study used tangential flow filtration (TFF), asymmetrical flow field flow fractionation, or microfluidics. 32 studies (47.1%) combined two or more separation techniques to achieve higher purity (Figure 5B).

#### Characterization of sEV preparations

MISEV2018 recommends characterizing EVs using complementary approaches 14 to evaluate the outcome of separation methods, assess properties of EVs, and assess the extent to which biological functions can be attributed to sEVs versus co-separated materials. A diverse array of characterization procedures was used in the studies we reviewed (Figure 5C). Size distribution was determined by single-particle analysis methods such as nanoparticle tracking analysis (NTA) (n = 34, 50%), tunable resistive pulse sensing (TRPS) (n = 5, 7.4%), and atomic force microscopy (AFM) (n = 1, 1.5%), while others used ensemble methods such as dynamic light scattering (DLS) (n = 20, 29.4%). Morphology was checked by transmission electron microscopy (TEM) (n = 60, 88.2%), scanning EM (SEM) (n = 6, 8.8%), and cryo-TEM (n = 1, 1.5%). Protein quantification was done by bicinchoninic acid assay (BCA) (n = 36, 52.9%) or Bradford assay (n = 2, 3%). Surprisingly, most studies did not report sEV total protein yield.

EV-specific markers were detected by Western blot (62 studies, 91.2%), flow cytometry (12 studies, 17.7%), or both (six studies). The tetraspanin transmembrane proteins CD63 (n = 52), CD9 (n = 40), and CD81 (n = 38) were the most frequently examined markers (Figure 5D). Other positive markers included the cytosolic proteins TSG101 (n = 33), Alix (n = 17), and HSP70 (n = 7). Only 17 studies (25%) checked for the presence of negative or depleted non-EV markers, including Calnexin (n = 8), Grp94 (n = 3), GM130 (n = 4), Lamin (n = 1), and Calregulin (n = 1). A total of 26 studies (38.2%) examined four or more protein markers. Only one study evaluated the lipidomic profile of sEVs 83. Broader profiling of EV proteins 53, 83-86 or RNA (mainly miRNA) 44, 50, 52, 54, 69, 73, 74, 86-90 was also reported.

#### sEV administration and dosage regimen

##### Dose

The administered sEV dose differed widely. sEV amount was approximated as protein amount in most studies, ranging from 2 µg to 5 mg (n = 45, 66.2%). In 7 studies (10.3%), dose was reported as number of particles, ranging from 2×10^10^ to 2×10^12^ particles (n = 7, 10.3%). However, only one study explicitly took into account the size of the animal, reporting sEV dose as protein per animal weight (5 mg/kg) 85, and amount was not reported at all in 15 studies (22.1%). Dose-response was assessed in one trial with three doses of 25, 50, and 100 µg/ml of PBS 91 and in five studies (7.4%) with low and high doses 44, 52, 82, 92, 93. In these studies, wound healing was reported to be positively associated with dose.

##### Administration route

Local injection was the most prevalent approach (n = 47, 69.1%): subcutaneous (n = 32), intradermal (n = 3), and other (n = 12). sEV-loaded hydrogels were injected into the wound in seven studies 44, 70-74, 78. sEVs were topically applied in 17 studies (25%), either mixed with PBS (n = 2) or embedded in hydrogels or other scaffolds (n = 13). Intravenous (n = 3, 4.4%) and intraperitoneal (n = 1) routes were less common (Figure 3D). One study compared the influence of subcutaneous and intravenous administration, reporting superiority of intravenous administration 94.

##### Dosing frequency and intervention duration

The majority of studies (n = 51, 75%) involved a single dose. Of the multi-dose studies (n = 17, 25%) (Table 2), two compared repeated-dose vs single-dose administration, concluding that repeated administration of low doses outperformed a single high dose 44, 82. The intervention period was diverse ranging mostly from eight to 28 days (Table 2).

### Immuno-biocompatibility

Human sEVs were administered to immunocompetent animals in 57 studies (83.8%) (Figure 3C). Allogeneic sEVs (from the same species) were used in 11 studies (16.2%), whereas autologous sEVs (from the same subject) were investigated in only one study 42. Direct comparisons of the efficacy and immune response to autologous, allogeneic, or xenogeneic sEVs were inadequately considered in the included studies in this review, with only a single study comparing the therapeutic efficacy of sEVs from xenogeneic and allogeneic sources 51, and another comparing autologous sEVs with allogeneic sEVs 42. No difference in efficacy was found between xenogeneic and allogeneic sources 51. Autologous sEVs were reported to be more effective and viable in treated tissues than allogeneic sEVs, even though the latter had a sufficient therapeutic effect when compared with placebo 42. More studies are needed to verify these findings and investigate the mechanism behind.

### Labelling and tracking of sEVs *in vivo*

Only six studies (8.8%) reported tracking of transplanted sEVs 42, 44, 82, 86, 87, 94. In these studies, sEVs were pre-labeled with lipophilic fluorescent dyes, namely PKH26 (n = 3), PKH67 (n = 1), DiR (n = 1), and lipid conjugated Cy7-dipalmitoylphosphatidylethanolamine (DPPE). All but one of these studies 82 administered a single dose of sEVs. A wide span of time points (1 hour to 21 days) was investigated. sEVs were tracked *in vivo* in four studies 44, 86, 87, 94, *ex vivo* (post-mortem) in one study 42, and *in vitro* and *ex vivo* in another study 82. None of these studies examined the biodistribution of sEVs to organs other than the skin tissue around the wound area. See Table 4 for detailed findings about sEV bioavailability.

### Quality assessment

We sought to evaluate the quality of both methodology and reporting. Methodological biases may skew the outcomes of studies, resulting in misleading estimates of therapeutic efficacy and flawed inferences. Poor reporting impedes experiment evaluation and reproducibility. We thus evaluated several methodological aspects and compliance with established guidelines.

#### Quality of reporting

Quality of reporting was generally low. Most of the reviewed studies did not report pre-processing details such as donor number, age, and gender. For EV production, the number, seeding density, and passage number of EV-secreting cells were poorly reported, along with cell viability at harvest. EV depletion protocols were reported for only six of 22 studies that depleted exogenous EV collection medium. For EV separations, centrifugation details such rotor type, adjusted K factor, and the volume centrifuged were deficient. For *in vivo* experiments, no study indicated how the sample size for animal models was calculated. In 14 studies (20.6%), the sample size was not even disclosed. 15 studies did not reveal administered dose (22.1%). Several studies reported results for experiments that did not have a corresponding methodology section and thus could not be reproduced. In terms of outcome reporting, only seven studies (10.3%) reported the actual numerical data. All comparisons were depicted exclusively as graphical data presentations. In terms of statistical analysis, most studies did not report the absolute p-value and confidence interval of the measured outcomes.

#### Risk of bias assessment

**Figure 6:**
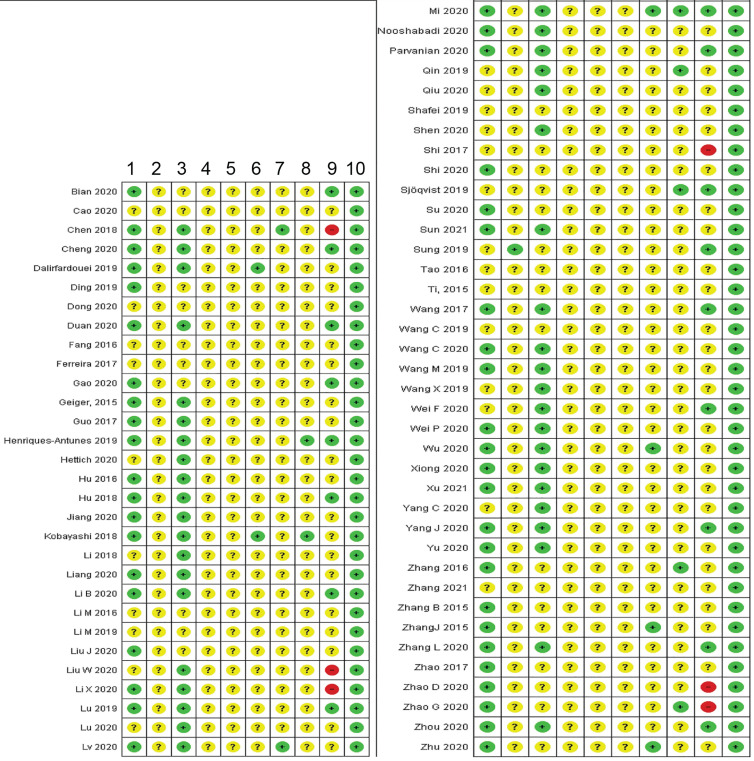
Risk of bias assessment of the 68 reviewed studies based on SYRCLE's ROB tool represented by RevMan 5.4.1. (1) Randomization (selection bias); (2) Random sequence generation (selection bias); (3) Baseline characteristics (selection bias); (4) Allocation concealment; (5) Random housing (performance bias); (6) Blinding of personnel (performance bias); (7) Random outcome assessment (detection bias); (8) Blinding of outcome assessment (detection bias); (9) Incomplete outcome data (attrition bias); (10) Selective reporting (reporting bias). A domain concerning the declaration of the randomization method was added (domain 1), while the domain of “other sources of bias” was not covered in this review. Symbols used: 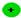: low risk; 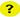: unclear risk; 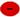: high risk.

We used the SYRCLE's ROB tool 95 to assess the risk of bias in animal experiments (Figure 6). Overall, there was an unclear risk of bias for most of the elements investigated. Randomization of animals was reported in 44 studies (64.7%) but without disclosing the randomization method. 24 studies (35.3%) did not report randomization. While 42 studies (61.8%) reported comparable baseline characteristics between control and experimental groups, judgment was not possible in 26 studies (38.2%) due to insufficient reporting of certain animal characteristics, particularly age, which is a determinant factor in wound healing.

None of the studies clarified if allocation was concealed, or if animals were randomly housed. Blinding while performing the experiments was reported for only two studies 96, 97. Six studies conducted random outcome assessment (mostly angiogenesis experiments) 43, 65, 69, 84, 88, 98. We identified a high risk of attrition bias in six studies (8.8%) 46, 55, 75, 76, 84, 99, low risk in 17 (25%), and an uncertain risk in the remaining 45 (66.2%). Blinding while assessing the outcomes was reported for seven studies (10.3%). Low risk was captured for all studies in relation to the selective reporting item, based on what was reported in the methods, although none of these studies reported publishing an *a priori* protocol to verify this judgment. The summary of the risk of bias assessment is shown in Figure 6.

#### Adherence to ISCT criteria for MSC characterization

To ensure comparability of studies of mesenchymal stromal cells (MSCs), the International Society for Cell and Gene Therapy (ISCT) has proposed minimal criteria to characterize and define these cells 40. Specifically, cells should: 1) show ability to adhere to plastic; 2) be positive for surface markers CD105, CD73, and CD90, and negative for CD45, CD34, CD14 or CD11b, CD79a or CD19, and HLA-DR; and 3) show *in vitro* multi-lineage differentiation capacity into osteogenic, adipogenic, and chondrogenic lineages under specific culture-differentiating conditions. Among the 68 included studies, 43 (63%) used MSC-derived sEVs. However, just over half of the MSC studies (n = 23) characterized cells as recommended by ISCT. 16 studies (23.5%) did not report any MSC characterization; in nine, MSCs were said to have been obtained commercially or from other institutes. One study investigated only two ISCT criteria, namely MSC adherence and surface antigen expression, and three focused exclusively on one ISCT criterion, namely surface antigen expression.

#### Adherence to MISEV2018 for sEV characterization, purity, and nomenclature

##### sEV characterization

To verify the identity of the isolated preparations, MISEV2018 indicates that EVs should be characterized by 1) concentration (such as protein and particle count); 2) at least two positive EV protein markers (including at least one transmembrane and one cytosolic marker), plus at least one source-appropriate negative, non-EV protein marker; and 3) two complementary single-vesicle analysis techniques to assess morphology and biophysical properties such as counts and size distribution 14. Of the 68 studies analyzed here, only 14 (20.6%) satisfied the above criteria 49, 52-55, 69, 82, 83, 88, 90, 96, 100-102.

##### sEV preparation purity estimation

MISEV2018 also suggests reporting protein:particle, lipid:particle, or lipid:protein ratios as surrogates of EV purity. Only two studies (2.9%) quantitatively estimated sEV purity according to MISEV2018, reporting particle:protein ratio 44, 83. Additionally, only 17 studies (25%) checked for the presence of negative/depleted (non-EV) markers that indicate the presence of non-EV contaminants.

##### sEV nomenclature

Since cells release EVs of varying sizes via different biogenesis pathways, and in the absence of specific, universal markers to distinguish EV subtypes, MISEV2018 recommends the term “extracellular vesicles” 14. MISEV2018 also encourages that EVs be further described by physical properties (such as size), biochemical makeup, source cell, or culture condition. Historical and variously defined terms such as exosome and microvesicle are discouraged unless biogenesis can be proven. Here, we included studies that investigated the therapeutic potential of “small” extracellular vesicles (30-200nm) in wound healing and skin regeneration. 56 studies (82.4%) used the term “exosome” to describe the preparation without presenting clear justification for use of this term. This included 78% (39/50) of the studies published in 2019 onwards, following the MISEV2018 release. 12 studies (17.6%) used the term “extracellular vesicles” 43, 44, 49, 51, 53, 86, 100-105, 11 of which were published in 2019 or later. Of these, four followed MISEV2018 nomenclature by size identification and specified that they were small extracellular vesicles 44, 49, 51, 100, while the remaining studies described them by the cell of origin and culture condition.

##### Reporting to EV-TRACK

MISEV2018 highly encourages submitting methodological details to EV-TRACK, a crowdsourcing tool developed to enable reproducibility and understanding methodology and experimental outcomes 14, 106. An “EV-METRIC” is assigned to each submitted study based on the proportion of required methodology details that are submitted. Here, only one study (1.5%) reported submitting details to the EV-TRACK knowledgebase 82.

### Outcomes

#### Wound healing

61 studies (89.7%) investigated wound closure (Table 3). 60 studies reported that sEVs significantly accelerated wound healing at at least one time point (*p* < 0.05 and as low as 0.001), while a single study found this effect to be not statistically significant when compared with experimental placebo-control and liposomes 83. While one study detected substantial enhancements at an early stage of the wound healing process but not at the endpoint 105, others observed these enhancements exclusively at 47, 93 or near the endpoint 55, 69, 72, 91, 96, 100, 107. Several studies reported improvement at all time points, ranging from day 2 to day 21 post wounding 44, 52, 57, 60, 80, 87, 88, 103. Surprisingly, reporting of precise complete wound closure time was frequently overlooked, as only 12 studies reported wound closure timepoints. Total closure of diabetic wounds was reported on day 14 62, 72, 92 and day19 97. Diabetic wounds were observed to be “almost closed” on day 14 68, 76, 80, 103 and day 18 79. Meanwhile, complete non-diabetic wound closure was noted on day 8 53, day 14 49, 104, 105, 107, day 21 74, 91, and day 25 73, and “almost closed” on day 10 61, 85 and day 14 75, 77, 81, indicating no major differences between diabetic and non-diabetic wound closure time in the examined studies.

#### Re-epithelialization

Of the 38 studies (56%) that evaluated re-epithelialization, 37 indicated improvements as a result of sEV intervention. These studies also noted enhanced granulation, culminating in well-formed tissue that resembled the native tissue in thickness and cellularity. Several studies demonstrated that sEV-treated wound beds had significantly enhanced cellularity 45, 84, which gradually reduced towards the end of the study 96. Higher levels of proliferation and migration protein markers such as cytokeratin 14 44, 52, cytokeratin 19 45, 46, 108, cytokeratin 10 99, PCNA 45, 46, 100, 108, Ki67 61, 71, 72, 74, 84, and CXCR4 100, as well as lower p21 expression 100 were detected *in vivo*. Additionally, twelve studies reported neogenesis of appendages including hair follicles and sebaceous glands. In contrast, a single study observed no clear improvement in re-epithelialization after treatment with sEVs 83. However, the same study showed considerable improvement in mature collagen deposition in the sEV-treated group.

#### Collagen deposition

The degree of collagen deposition was investigated in 45 studies (66.2%). While 25 studies quantified either collagen deposition or expression in sEV treated tissues, the remaining 20 studies described it qualitatively. 34 investigations evaluated total collagen deposition, organization, and maturation using Masson's trichrome staining. Nine studies determined the presence of messenger RNA (mRNA) encoding collagens type I and III in sEV-treated tissues by RT-qPCR. Other techniques were Western blotting (n = 7), immunohistochemistry (n = 6), picrosirius red staining (n = 3), and Herovici staining (n = 1). All 45 studies reported enhanced collagen deposition, maturation, and organization following sEV administration in diabetic and non-diabetic rats. Immature collagen fibers were observed near the wound bed shortly post-treatment and improved significantly in alignment and maturity as healing progressed compared with the control, with an increased mature-to-young fiber ratio as early as five days after wounding 83.

Only 20 studies examined collagen deposition at multiple points, and most frequently at days 7, 14, 21, and 28, while a single study investigated collagen deposition for up to five weeks 46. Some studies reported a trend towards increased collagen deposition towards the endpoint 52, 70, 76, 92, 98, but others showed a declining trend 63, 94. Similarly, mRNA expression of collagen type 1 showed a spike followed by a gradual decline towards the endpoint 74, 96, 109, while collagen type III expression varied among studies 48, 74, 94, 96, 109.

#### Scar formation

The extent of scarring in tissues that received sEVs was assessed in 22 studies (32%), all of which concluded that sEVs effectively minimized scar formation. Scars were evaluated quantitatively by measuring the width 54, 64, 69, 84, 87, 88, 93, 96, 98, 100, 102, 104, 109, depth 96, 109, length 73, height 43, or area 96, showing a significant reduction in the measured index versus controls. No study reported hypertrophic or keloid scar formation in sEV-treated groups. In addition, collagen type I/collagen type III ratios were examined to assess fibrosis. One study noted higher collagen type I to collagen type III ratios in EV-treated groups 45, but three observed the reverse 74, 96, 109. Several studies examined α-smooth muscle actin (α-SMA) levels to evaluate myofibroblasts in treated tissues 43, 58, 73, 74, 109. α-SMA was significantly reduced, suggesting that sEVs suppressed myofibroblast aggregation in wounds. Reduced levels of proteolytic enzymes, i.e., MMP-9 92, MMP-13 49, MMP-1 and MMP3 48, were also reported, while levels of transforming growth factor-β (TGF-β) varied among studies 67, 72, 74, 85, 109.

#### Angiogenesis

45 studies (66%) examined vascularization of newly formed tissues, reporting a significant improvement in both diabetic and non-diabetic wounds that was associated with sEV treatment. These studies detected a rise in new vessel density, quantified by positive markers like CD31 (n = 35), α-SMA (n = 18), CD34 (n = 4), and Meca32 (n = 2). 12 investigations identified mature blood vessels as those positive for both CD31 and α-SMA, with substantial enhancement approaching the study endpoint. Several studies also showed upregulated vascular endothelial growth factor (VEGF) 67, 72, 78, 79, 96, 104. Marker detection techniques included immunohistochemistry (n = 20), immunofluorescence (n = 25), and Western blotting (n = 3). Techniques to assess neovascularization were microfilm perfusion and micro-CT scanning (n = 9) and small animal doppler detection (n = 1). Hettich *et al.* observed an enhanced vascularization localized to the wound margins where sEVs were injected, implying a possible enhanced local impact near the injection site 83.

#### Inflammation

A limited number of studies (n = 16, 23.5%) assessed inflammation. Overall, sEVs exerted an anti-inflammatory effect via different mechanisms. sEVs promoted the transition of macrophages from M1 (pro-inflammatory) to M2 (anti-inflammatory) phenotypes, as evidenced by a higher M2:M1 ratio 50, 55, 96, increased expression of arginase (ARG) to inducible nitric oxide synthase (iNOS) 96, and CD206- to CCR7-positive cells 55. Decreased infiltration of inflammatory cells, predominantly macrophages and neutrophils 50, 65-67, 72, 85, 92, was observed in sEV-treated groups, with no noticeable presence of these cells towards the endpoints of two studies 65, 92. In contrast, inflammatory cells were abundant in control groups. These findings were in line with downregulated expression profiles of pro-inflammatory mediators such as tumor necrosis factor (TNF) α 53, 59, 61, 67, 85, 92, IL (interleukin)-6 53, 59, 61, 85, 92, IL-1 49, 59, 67, MCP-1 59, Toll-like receptor 4 and p-P65 expression 50 in groups receiving sEVs. At the same time, anti-inflammatory factors were elevated: IL-10 67, 85 and PTEN 55, 67 as well as activation of p-STAT3 and p-AKT 50. Both MSC-derived sEVs (n = 9) and non-MSC-derived sEVs (n = 7) showed immunomodulatory effects.

#### Adverse events

No harmful events were reported by any study. 12 studies (17.6%) presented information related to adverse event evaluation, four of them having pre-specified in advance that the potential for harm would be investigated 57, 59, 61, 96. Yu *et al* noticed no erythema, edema, or irritation in skin tissues receiving sEV local injections, and no increase in renal injury markers (creatine and BUN) or liver function indicators (ALT and AST) 57. Su *et al*. evaluated CD4+ and CD8+ T cell counts in the spleen and lymph nodes proximal to the treated sites in mice and found no difference between treatment with unmodified EVs and a negative control, although they observed a slight decrease of CD8+ T cells in the lymph of animals treated with EVs overexpressing PD-L1 61. One study monitored overall well-being and behavior of mice 96, whereas another checked for indicators of degeneration and necrosis 59. However, neither study reported the outcome. Three studies assessed apoptosis in treated skin tissues using TUNEL assay 67, 68 or by evaluating Bcl-2 and iBax levels 78, all of which showed a reduction in apoptosis. Four studies made generic statements that no negative effects or discomfort 60, 62, 80, 104 or no impact on body weight 58, 82 were detected. Lastly, one study reported that allogeneic iPSC-derived sEVs did not endow recipient cells with pluripotency or elicit immune rejection following repeated doses over 14 days of treatment 42.

#### The effect of modifications on the “characteristics” and “therapeutic outcomes” of sEVs

##### Preconditioning

Nine studies employed different preconditioning regimens as elaborated earlier (Section 2.4.2.a). Six (8.8%) found that preconditioning improved efficacy by promoting wound closure, re-epithelialization, collagen deposition, and angiogenesis 50, 53-57. One study found that MSC-derived sEVs were more potent when parent MSCs were preconditioned with neonatal serum sEVs versus adult serum sEVs 58. Additionally, effects of preconditioning on sEV size distribution, cargo components, marker expression, concentration, and release profiles were examined, suggesting that preconditioning modified sEV cargo composition but not size distribution 50, 53. Priming cells with PDGF-BB, TGF-β1, FGF2 52, LPS 50, 53, thrombin, H_2_O_2_, hypoxia with 1-10% O_2_ 53, and PTHrP-2 56 appeared to increase sEV release, whereas treatment with melatonin 55 and ATV 57 did not. Most studies reported that preconditioning did not alter sEV marker profiles 50, 54, 55, 57. However**,** Geiger *et al*. showed that the immunogenic surface markers MHC class I, MHC class II, CD80, and CD86 were deficient in growth factor-stimulated fibrocyte-sEVs compared with their unstimulated counterparts 52. Also, Sung *et al*. detected an increase in mitochondrial cytochrome C expression in sEVs from H_2_O_2-_ and hypoxia-primed cells, but not in LPS, naïve, and thrombin groups 53. In that study, a comparison of the different preconditioning agents revealed a superior effect of thrombin, highlighting the importance of optimizing preconditioning regimens.

##### Genetic modification

11 studies (16.2%) examined the impact of genetic manipulation of producing cells on the *in vivo* activity of released sEVs. Manipulation of parent cells affected the cargo of released sEVs. Five studies revealed a proangiogenic role of the engineered components, including miR-126-3p 60, 62, miR-126* 64, Nrf-2 63, and angiopoietin-2 47. Other studies established the involvement of manipulated proteins and non-coding RNAs, notably TSG-6 59, PD-L1 61, and lncRNA H19 67, in inflammation regulation or autophagy activation 68. Upon comparison with sEVs from naïve cells, overexpression of Nrf-2 63, angiopoietin-2 47, miR-126* 64, TSG-6 59, PD-L1 61, lncRNA H19 67, and mmu_circ_0000250 68 conferred accelerated wound closure efficacy to sEVs 47, 61, 63-68, enhanced re-epithelialization 47, 61, 65, 66 and/or angiogenesis 47, 63-65, 67, 68, or suppressed inflammation 59, 61, 65, 67 and scar formation 59, 64. Importantly, none of these studies reported adverse effects.

* The arm was not specified.

##### Nanoparticle loading

Superparamagnetic iron oxide nanoparticles (Fe_3_O_4_-NPs) were reported to be efficiently loaded into parent cells and sEVs 46, 69. Accumulation of membrane-encapsulated nanoparticles was demonstrated in the cytoplasm 46, 69 and nucleus 69 of sEV-producing cells. The use of nanoparticles and static magnetic fields significantly enhanced the beneficial impact of sEVs when administered locally 69. Analyzing sEV content revealed an abundance of miRNA content, predominantly miR-21-5p, compared with their naïve-sEV counterparts 69, as well as increased sEV release but with no changes to size distribution 69. However, loading NPs into sEVs alone did not appear to improve their performance when introduced intravenously, as noted by Li *et al.* Improved wound closure rate, re-epithelialization, neovascularization, and collagen deposition was observed only after employing magnetic guidance, which apparently enhanced targeting and localization 46.

##### Incorporation of sEVs into biomaterial scaffolds

20 studies examined the efficiency of sEV-functionalized biomaterial scaffolds on wound healing. sEV-loaded scaffolds demonstrated superior therapeutic potential compared with either sEVs or scaffolds alone. A considerable improvement in wound closure (n = 20), new blood vessel formation (n = 14), re-epithelialization (n = 16), and collagen deposition (n = 17) was reported. 16 studies compared gel-only with gel-sEV preparations, while only three studies included sEV-only preparations, demonstrating superior effectiveness of gel-sEV preparations. Interestingly, Henriques-Antunes *et al*. indicated that light-triggered hydrogel-sEV preparations significantly outperformed preparations of hydrogel-sEV alone 44. In addition, hydrogels enriched with sEVs were as potent as hydrogels loaded with bFGF in promoting wound healing 61.

#### A head-to-head comparison with source and conditioned media (secretome)

Few studies attempted to compare the performance of enriched sEV preparations in promoting skin healing and regeneration with that of their source and conditioned medium, i.e., the total secretome. Interestingly, seven studies compared sEV efficacy with that of their source (menstrual blood-MSCs, platelet-rich plasma (PRP), iPSCs, saliva, hADMSCs, and hucMSCs) 42, 45, 80, 88, 96, 99, 107. Four studies detected an overall equivalent effect of sEVs and their source cells on wound closure, re-epithelialization, and collagen deposition 42, 45, 99, 107. However, Dalirfardouei *et al*., observed that sEVs were superior at inducing wound closure rate, neovascularization, and M1 to M2 polarization 96. Similarly, PRP and saliva-derived sEVs appeared to outperform PRP and saliva respectively in promoting wound closure, angiogenesis, and neo-epithelialization 80, 88. Just two studies examined the efficacy of sEVs versus conditioned medium. While unfractionated conditioned medium was found to have a similar beneficial effect on wound closure as sEVs, with enhancement of angiogenesis and epidermis thickness 99, EV-depleted conditioned medium had an impact inferior to sEVs but comparable to that of a control 99, 109.

### Meta-Analysis

**Figure 7:**
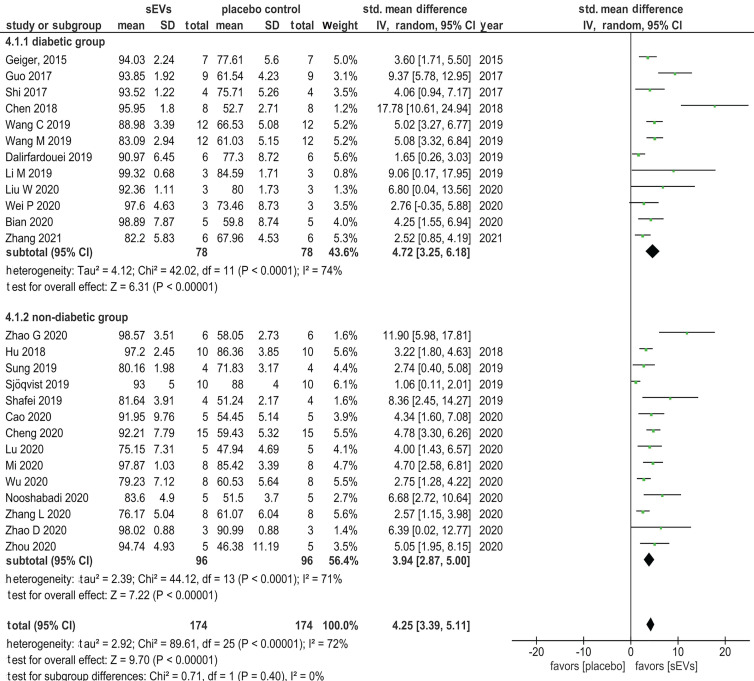
Forest plot of mean difference of wound closure rate of 26 studies following sEV interventions in diabetic or non-diabetic skin wound models in comparison to placebo controls. The diamond represents the pooled SMD. sEV interventions were effective in promoting wound closure, (pooled SMD = 4.25, 95% CI: 3.39 to 5.11, *p* < 0.00001).

26 studies were eligible for meta-analysis of wound closure outcome, involving 174 animals from 12 diabetes and 14 non-diabetes model studies. An overall significant enhancement of wound closure rate was scored for wounds treated with sEVs (SMD = 4.25, 95% CI: 3.39 to 5.11, *p* < 0.00001) in comparison with control (Figure 7). The heterogeneity index was relatively high (I^2^ = 72), reflecting the variability in sEV source, preparation, and dosage regimen. Similarly, subgroup meta-analyses in diabetic and non-diabetic groups demonstrated that sEV therapy was significantly more effective than control in accelerating wound closure in both models (SMD = 4.72, 95% CI: 3.25 to 6.18, *p* < 0.00001; SMD = 3.94, 95% CI: 2.87 to 5.00, *p* < 0.00001 in diabetes and non-diabetes models, respectively). Heterogeneity was likewise high in subgroups regardless of the disease model (I^2^ = 74%, and 71% in diabetes and non-diabetes models, respectively).

**Figure 8:**
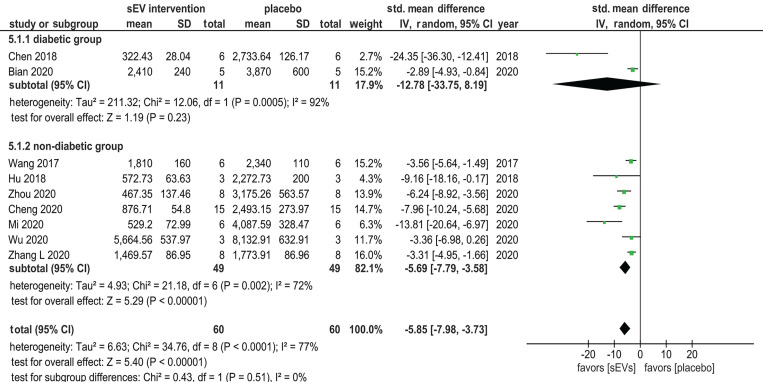
Forest plot of mean difference of scar width (in µm) of nine studies following sEV interventions in diabetic and non-diabetic wound models in comparison to placebo controls. The diamond represents the pooled SMD. sEV interventions were effective in inhibiting scar formation, (pooled SMD = -5.69, 95% CI: -7.79 to -3.58, *p* < 0.00001).

Among all included studies, 22 assessed scar formation, of which 13 studies used scar width (in µm) as the scar assessment metric for examining the influence of sEV interventions. Of the 13 studies that used this metric, nine studies reported sample size and were included in the meta-analysis (a total of 60 animals; two studies used diabetes models, and seven used non-diabetes models). Overall, sEV therapy resulted in a substantial decrease in scar width compared with controls (SMD = -5.85, 95% CI: -7.98 to -3.73, *p* < 0.00001). However, when subgroup analysis was performed, the difference in scar width between the control and experimental groups in diabetes studies was insignificant (SMD = -12.78, 95% CI: -33.75 to 8.19, *p* = 0.23). Indeed, the results of only two studies (11 animals) are insufficient to draw conclusions from a meta-analysis, particularly given the high heterogeneity index (I^2^ = 92%). In comparison, sEVs significantly inhibited scar development in the non-diabetic group (SMD = -5.69, 95% CI: -7.79 to -3.58, *p* < 0.00001). Overall heterogeneity of the effect was high (I^2^ = 77%) (Figure 8).

**Figure 9:**
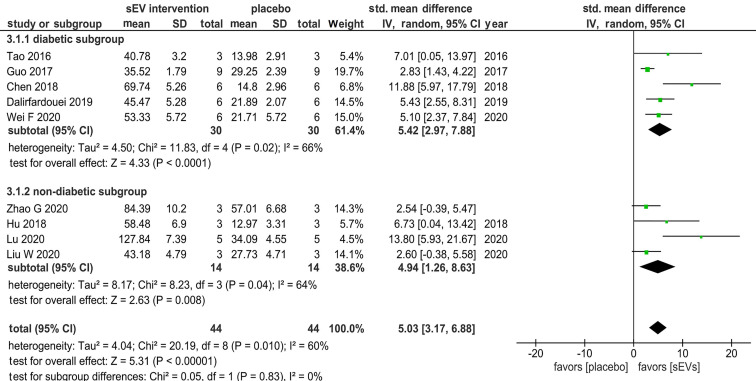
Forest plot of mean difference of blood vessel density (number of blood vessels/mm^2^) of nine studies following sEV interventions in diabetic and non-diabetic wound models in comparison to placebo controls. The diamond represents the pooled SMD. sEV interventions were effective in promoting new blood vessel formation, (pooled SMD = 5.03, 95% CI: 3.17 to 6.88, *p* < 0.00001).

Nine studies assessing blood vessel density (number of blood vessels/mm^2^) to evaluate the effect of sEV transplantation on angiogenesis were eligible for meta-analysis (44 animals), and subgroup analysis (5 diabetes model studies of 30 animals; 4 non-diabetes model studies of 14 animals) was thus performed. A meta-analysis revealed an overall significant impact of sEVs in supporting blood vessel development (SMD = 5.03, 95% CI: 3.17 to 6.88, *p* < 0.00001). The heterogeneity index was moderate (I^2^ = 60%). In subgroup analysis, both diabetic and non-diabetic subgroups demonstrated a significant positive effect of sEV treatment compared with control (SMD = 5.42, 95% CI: 2.97 to 7.88, *p* < 0.0001; and SMD = 4.94, 95% CI: 1.26 to 8.63, *p* = 0.008; in diabetes and non-diabetes models, respectively) (Figure 9). Heterogeneity indices in the two groups were moderate (I^2^ = 66% and 64%, in the diabetic and non-diabetic groups, respectively).

**Figure 10:**
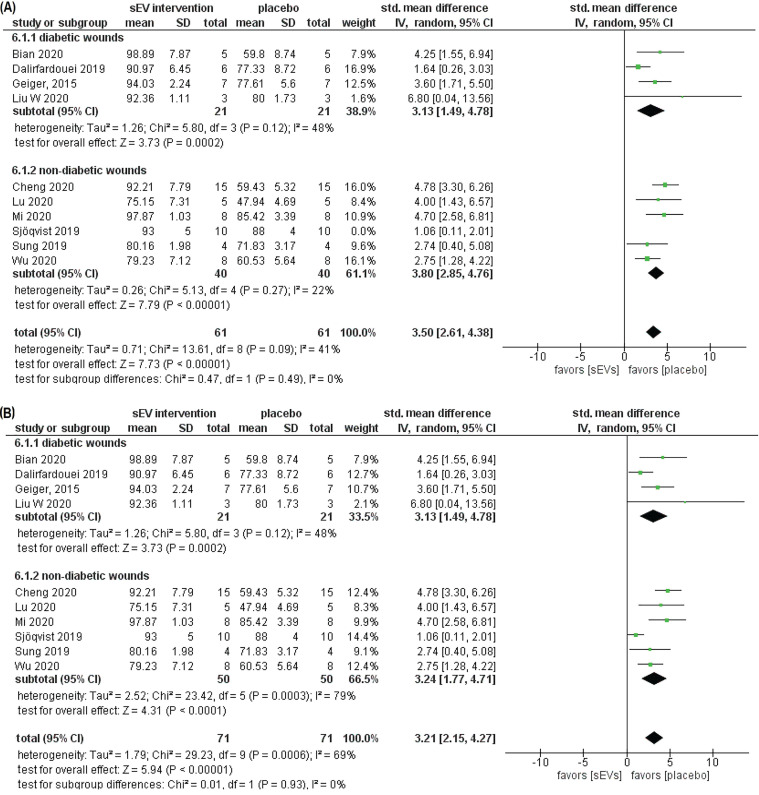
** A)** Forest plot of mean difference of wound closure rate in diabetic and non-diabetic wound models of studies that characterized sEV based on MISEV 2018. The diamond represents the pooled SMD. A) sEV interventions were effective in promoting wound closure, (pooled SMD = 3.50, 95% CI: 2.61 to 4.38, *p* < 0.00001). **B)** A sensitivity analysis resulted in excluding one study 82, causing considerable heterogeneity in the meta-analysis (Figure 10A, without the study, and Figure 10B with the study, I^2^ = 41% vs 79%).

**Figure 11:**
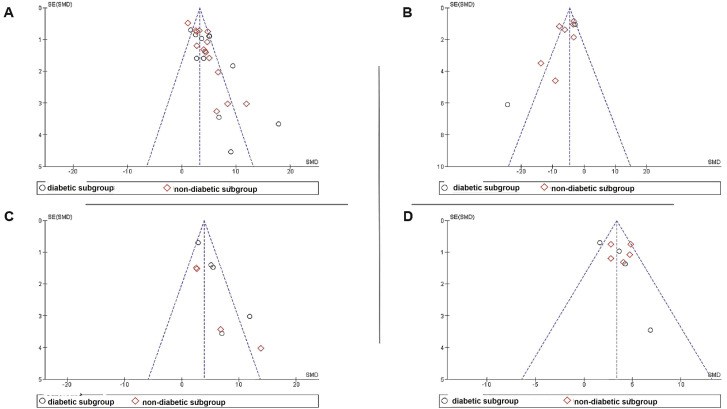
** Funnel plot for the assessment of publication bias. a)** Funnel plot of the studies included in wound closure rate meta-analysis. **b)** Funnel plot of the studies included in scar width meta-analysis. **c)** Funnel plot of the studies included in angiogenesis meta-analysis d) Funnel plot of the studies included in wound closure rate meta-analysis for studies that characterized sEV based on MISEV 2018. The funnel plots for the four meta-analyses performed showed no evidence of publication bias.

We also performed meta-analyses of the wound closure rate outcome for studies that 1) characterized their EV preparation as required by MISEV2018 (section 2.7.4.a) and 2) disclosed the number of animals used in the experiments. A total of ten studies were considered eligible by these criteria (4 studies of 21 diabetic animals and 6 studies of 50 non-diabetic animals). We performed a sensitivity analysis that resulted in excluding one study 82, which produced considerable heterogeneity in the meta-analysis. Thus, only nine studies were included in the meta-analysis (4 studies of 21 diabetic animals and 5 studies of 40 non-diabetic animals) (Figure 10). Consistent with our earlier findings, sEV intervention had a substantially favorable influence on wound closure across all studies (SMD = 3.50, 95% CI: 2.61 to 4.38, *p* < 0.00001) and in subgroup analyses (SMD = 3.13, 95%CI: 1.49 to 4.78, *p* < 0.0002; SMD = 3.80, 95% CI: 2.85 to 4.76, *p* < 0.00001) for diabetes and non-diabetes animal models, respectively (Figure 10A). The sEV interventions, on the other hand, had a more homogeneous effect, as demonstrated by the lower I^2^ statistics in each subgroup (I^2^ = 48, and 22%, respectively) as well as in the overall meta-analysis (I^2^ = 41%), in contrast to the higher heterogeneity observed in the overall analysis of wound closure that also included studies that did not comply with MISEV2018 (Figure 7). The funnel plots for the four meta-analyses showed no evidence of publication bias (Figure 11).

## Discussion

We have systematically reviewed the available evidence on therapeutic efficacy and safety of sEVs in wound healing and skin regeneration using animal models. We summarize recent research in this area and critically appraise the quality of the included studies, with an emphasis on methodology, reporting quality, and compliance with related guidelines. By doing so, we inform the scientific community of the main findings and the quality of evidence provided by the current literature. We detected a recent exponential surge in publications exploring sEV therapeutic potential in wound healing and skin regeneration, highlighting growing interest and enthusiasm towards sEV research in this area. 68 studies met our inclusion criteria, exploring a diverse spectrum of sEVs: not only from MSCs, but also from other cell types, as well as from complex tissues and biofluids. Although separated EV preparations contain mixed populations of sEVs, and not only exosomes (of endosomal origin), the bulk of the analyzed studies continued to describe their preparations as “exosomes,” to which they related the observed functionality. In absence of techniques capable of identifying EVs from distinct intracellular origin 110, it is likely that these particles were instead a broader population of sEVs 14, 26. Throughout this discussion we will highlight unresolved issues and address several crucial questions, beginning with this central, fundamental question:

### Was sEV intervention therapeutically effective?

Overall, our systematic review and meta-analysis concluded that sEV intervention had significant efficacy in promoting skin regeneration in diabetic and non-diabetic animal models. This finding agrees with earlier systematic reviews that examined the therapeutic efficacy of MSC-derived sEVs in wound healing in general 111 and diabetic wound healing in particular 112. Based on our analysis, sEV intervention targets multiple features of the intricate healing process, resulting in enhanced regeneration and suppressed fibrosis.

As prior research established that deficient vascularization is a key contributor to the chronicity of diabetic lesions 113, it was remarkable to observe the proangiogenic effect of sEVs on diabetic wounds, which was on par with the effect on non-diabetic wounds. This was supported by our meta-analysis findings that quantitatively revealed that there was no substantial difference between diabetic and non-diabetic models. Diabetic lesions usually exhibit diminished levels of VEGF, which contributes to compromised angiogenesis 114. sEV interventions boosted blood vessel regeneration and maturation with increased expression of VEGF.

Only a few studies addressed the immunomodulatory effects of sEVs. Nonetheless, those studies provided evidence for sEV modulation of the inflammatory milieu, favoring the transcriptional transition of pro-inflammatory M1 to anti-inflammatory M2 macrophages and reduction of immune cell infiltration. This is crucial for the inflammatory phase to resolve and the subsequent proliferative phase to commence, as persistent inflammation is a typical feature of non-healing wounds 115. Accordingly, sEVs reduced the production of pro-inflammatory mediators such as TNFα, IL-1, and Toll-like receptor 4 (TLR4), whilst elevating levels of anti-inflammatory counterparts such as IL-10. It is worth noting that this immunomodulatory activity was not limited to MSC-derived sEVs. More studies are needed to explore the potential and mechanisms of sEV interventions on inflammation in this context.

Furthermore, sEVs promoted re-epithelialization by enhancing skin cell proliferation and extracellular matrix secretion. sEVs fostered deposition of collagen, especially collagen type I, the major structural protein of the skin, and supported collagen maturation and organization. Although collagen production is critical for efficient wound closure, excessive production may lead to tissue fibrosis and scarring 116. In ideal healing scenarios, collagen production increases during the proliferation stage, then decreases and matures during the remodeling stage. Nonetheless, our evaluation of the reports on collagen expression levels and ratios of collagen type I/III at later phases of healing revealed discrepancies among studies, demanding further investigation. Even so, by examining scar formation macroscopically and histologically, a number of studies noted a considerable reduction in scar indices following sEV application, supporting the anti-scarring role of sEVs. Myofibroblasts, which are key contributors in collagen deposition and wound contraction 117, were reported to be suppressed, and there was no evidence of fibrosis or hypertrophic scar development. Most studies had relatively short follow-up periods (14- or 21-days post-wounding), though, limiting their ability to thoroughly assess collagen and scar development and maturation in healing tissues. It is estimated that scars in rodent models mature within 70 days of injury 118. Hence, it is necessary that relevant endpoints be defined and validated for future studies evaluating sEV usefulness in minimizing scarring and promoting tissue maturation. We observed that the protein marker α-smooth muscle actin (α-SMA) was used both to examine myofibroblast abundance and to mark blood vessel formation, revealing suppressed levels in the former and elevated levels in the latter. This creates some uncertainty over the actual expression of this marker in response to sEVs. Further examination of the utility of α-SMA as a differential marker may be needed.

Apparently, sEV modulation of the different healing mechanisms improved wound closure and tissue regeneration, with no differences in closure times for diabetic and non-diabetic wounds. sEVs likely improved the wound microenvironment, eventually encouraging tissue repair 118. However, some studies found wound closure acceleration throughout the follow-up period, and others only during the early or late stages of healing. This could be due to heterogeneity of sEV sources, preparation methods, and delivery strategies, necessitating additional head-to-head comparisons. Given the challenges of restoration of appendages in adult skin 119, 120, it was remarkable that sEVs supported the regrowth of hair follicles and sebaceous and sweat glands, indicating high-quality skin repair 119.

Comparisons with other treatment modalities were very limited. Interestingly, one study reported superior reparative activity of sEVs compared with the FDA-approved PDGF-BB therapy 44. Also, comparisons of sEV preparations with their cell sources demonstrated comparable, if not superior, efficacy in stimulating skin repair and regeneration. Such comparisons are necessary to establish the relative value of sEVs over cells. Theoretically, cells may be more beneficial than EVs: although EVs have a “set message” that may not further respond to the microenvironment, cells might perceive signals from the milieu and respond by releasing various factors, including EVs. Thus, observations of sEV efficacy versus cells are crucial to establishing comparative value. Efficacy could be due in part to EVs being less immunogenic and carrying a unique payload that may be delivered in trace levels, yet exert a profound impact 121.

To summarize, our in-depth analysis of the various aspects of wound healing and skin regeneration indicates the usefulness of sEVs as regenerative agents to promote skin repair. In the context of diabetic wounds, the evidence reviewed demonstrated that sEV interventions may overcome the barriers to lesion repair. Nevertheless, longer durations of examination may be required to effectively establish the influence on tissue maturation.

While some studies evaluated native sEV efficacy, others modified EV content and functionality, bringing us to the following question:

### Could modifying sEVs influence their therapeutic efficacy?

Modifying cells to improve their therapeutic qualities has been extensively investigated in recent years, with encouraging results. Several studies examined if manipulation of EV-producing cells by a variety of strategies translated into improved therapeutic functionalities of released sEVs in wound healing and skin regeneration. Endogenous loading of sEVs was achieved by physical loading with nanoparticles or genetic modification to overexpress certain proteins or nucleic acids. However, efficiency of loading and quantitation of cargo were seldom assessed. Furthermore, priming cells with assorted physiological and pharmacological cues showed a differential influence on the release profile, payloads, and hence downstream functionality of released sEVs 53, emphasizing the need for careful protocol and agent selection. Culturing MSCs with sEVs from neonatal versus adult sources resulted in more potent regenerative potential of released EVs 58, showing the importance of stimulant selection in sEV production. Notably, sEVs derived from modified cells had improved healing properties compared with their non-modified counterparts at all levels (re-epithelialization, proliferation, angiogenesis, collagen deposition, and maturation). It was not clear, however, if these modifications affected the stability, half-life, targeting (tropism), or internalization of these sEVs in injured tissues. On the other hand, encasing sEVs with biomaterials boosted their efficacy, possibly due to an enhanced release profile or other properties.

Indeed, the capability to manipulate EVs has sparked interest in creating “designer EVs” as a means to achieve desirable properties compared with native EV forms, including using them as drug delivery vehicles 29. Besides manipulating parent cells (i.e., endogenous loading) as described by most studies here, manipulating sEVs post isolation is also possible. Such exogenous loading has been attempted passively 122 and actively (e.g., by sonication 123), with varying success rates and loading efficiencies. In an exhaustive review aimed at developing a preliminary set of guidelines to standardize reporting of exogenous EV loading, Rankin-Turner *et al*. were surprised at the paucity of research into fundamental parameters such as incubation time and temperature, along with the variability and inconsistency in strategies 124. They stressed the need for optimizing loading procedures, which we also fully endorse.

In the midst of numerous studies aimed at improving sEV function, none reviewed here explored altering the sEV surface to enhance targeting, retention, or delivery, or to prevent non-target uptake to prolong EV half-life. For example, conjugating EVs with polyethylene glycol (PEG) might enhance stability in the circulation and reduce build-up in the liver 125, most probably by masking scavenger receptors. Likewise, EV CD47 extends half-life by signaling macrophages, “do not eat me” 126. EV surface modification has been done by chemical modification (CLICK chemistry and enzymatic conjugation) 127 and membrane cloaking 128. These strategies may be worth investigating in the future to increase sEV stability and targetability in skin tissues.

### Translational challenges to sEV therapy development

Despite encouraging findings, we have identified translational challenges that must be addressed before moving forward with human clinical trials.

#### Source

What is the ideal source of sEVs? In our review, sEVs were derived from tissues, biofluids, and cultured cells (Figure 4), but research comparing the efficacy of different preparations is lacking. Only a single study compared the efficacy of human umbilical cord-derived-MSCs with human lung fibroblasts (HFL1), concluding that MSC-EV preparations were more potent in enhancing skin regeneration 45 and suggesting source-dependent variation in activity. This emphasizes the need for evidence-based source selection. No studies examined the effect of 3D culture or bioreactors as scalable culture approaches for generating sEV skin therapeutics. Additionally, production of biological therapeutics requires verification of source identity. We examined compliance with ISCT guidelines for minimal identification of MSCs 40, finding that the criteria were met by just over half of the MSC-EV studies. To exclude heterogeneous and non-MSC cell populations, these criteria are important 129, 130, and ongoing efforts to develop standardized EV potency assays are crucial 130.

#### Depletion of exogenous EVs

*In vitro*, serum-derived EVs from culture medium additives may contaminate EV isolates. These EVs might not negatively affect the potency of therapeutic EVs, but their removal may be necessary to ensure reproducibility. Also, contamination with xenogeneic EVs (e.g., from FBS) could complicate translation. Almost half of the studies included in this systematic review collected sEVs from serum-free media. While this is a good strategy to minimize contaminating EVs, switching to a serum-free condition might starve and stress the cells 131, modifying EV release profile and biological properties 132. Depleting FBS of EVs before supplementation, primarily using ultracentrifugation, can also alter the activity of cultivated cells 133, although gradual transitioning can help 129. However, depletion is rarely complete. Driedonks *et al*. revealed that various depletion strategies had a varying influence on the concentration and types of fetal calf serum RNA contaminants in medium and that optimizing purification techniques can result in lower contaminant levels 134. While a minimum of 18 hours of ultracentrifugation was recommended to remove bovine EVs 135, depletion of only 70 minutes to three hours was reported in studies we examined. Additionally, contaminants can be present even in serum-free and so-called “chemically defined” media 136. Because some of the reviewed studies did not disclose their depletion protocols, we cannot fully assess the degree of purity of EV preparations. Indeed, MISEV2018 asserts the importance of specifying the precise source, procedures, and reference for depleted components, as well as the significance of verifying the “EV-free” status (or otherwise) of all supplements 14.

#### Separation and purification

Separating a specific EV subpopulation effectively while eliminating non-EV impurities may be one of the biggest technical hurdles in developing EV therapeutics 137, 138. Since “the process is the product” 139, different preparation procedures, even starting with the same source, might yield a diverse mixture of co-isolates and sEV subsets 140 with varying attributes such as size and biogenic origin. Thus, different separation techniques could lead to functional and physical differences 140. While it is still not clear how the different sEV subgroups with varying roles (e.g. disposal *vs.* signaling) and origin (endosome *vs.* plasma membrane) interact to influence the final healing outcomes 141, contaminants, on the other hand, were found to affect the therapeutic effect of EV preparations 142. Interestingly, it cannot be excluded that so-called contaminants might even contribute to desirable effects.

In part for this reason, MISEV2018 does not recommend a specific separation strategy, and combining multiple strategies may improve on single technique approaches. If the goal is to attribute all therapeutic effects to EVs only, high purity preparations should be obtained 14. Multiple pre-processing steps are needed to isolate EVs from complex biofluid or tissue sources, and thus specific protocols must be tailored to the particular source to remove contaminants before EV separation 143. The studies we reviewed used diverse separation procedures. Heterogeneity in the separation methods reflects a lack of standardization, hampering comparability and possibly delaying clinical translation 141. Classical methods like the various protocols of ultracentrifugation still predominate, even though this technique may result in aggregation 144 or functional 140 of EVs and has limited scalability 145. Precipitation-based concentration kits were also popular among the analyzed studies despite high protein contamination 146 and debatable usefulness 129, 146. Liangsupree *et al*. found a trend towards size exclusion chromatography (SEC) in recent years 147, but we did not observe this trend for the field that we reviewed. We also did not see uses of size-based separation techniques such as tangential flow filtration (TFF), SEC, or asymmetrical flow field-flow fractionation, or affinity-based technologies. Nonetheless, there was an increased reliance on method combinations and washes to improve purity.

Separation methods for sEV therapeutics should be selected for their scalability, automation potential, ability to optimize the purity and recovery of target sEVs 147, and translatability. Given the parallels between EVs and viruses, established virus purification techniques could be useful for separation and purification of EVs on a large-scale 145. Concerningly, none of the research reviewed here reported producing GMP-compliant sEVs, mirroring a recent systematic review of EV therapies for lung conditions 148. Unfortunately, high cost is not a valid excuse for lack of GMP compliance, as EVs will otherwise not be usable in the clinic.

#### Characterization

Given the inherent difficulties of standardizing sEV source and separation techniques, quantitative identity measures are required for improved post-separation sEV identification, quality control, and comparability. Characterization covers three aspects: identity, integrity, and purity 14. MISEV2018 guidelines recommend that, at a minimum, the concentration of EV preparations (such as protein and particle count) and EV markers (proteins expected to be enriched or depleted in EVs) should be determined. Of these markers: (1) at least two EV-protein specific markers (referred to as positive or enriched markers) must be detected to confirm EV isolation: one that is an integral membrane or membrane-anchored protein, signifying the presence of the lipid bilayer, and another that is an established cytosolic protein, implying the presence of enclosed cytoplasmic content and not just membrane fragments; and (2) at least a negative or depleted protein marker including source-related protein contaminants (such as albumin in EV plasma or uromodulin in EV urine samples), subcellular compartments, or cell death artifacts. Moreover, MISEV2018 suggests two complementary single vesicle analysis approaches to visualize and evaluate EV biophysical features. Here, preparations were mostly evaluated for protein concentration, particle count, morphology, and EV protein markers, employing multiple techniques; but just a few satisfied all MISEV2018 characterization criteria. To determine size distribution, nanoparticle tracking analysis (NTA) was the most-used method. Since our inclusion criteria stipulated that studies of sEVs with a size distribution beyond 30-200 nm were to be excluded, the studies that were included fell within this range. As different sizing platforms may have varying detection limits resulting in diverse outputs, employing multiple orthogonal technologies may be useful 149.

Although NTA was used to measure particle concentration, most studies reported protein concentration, but not particle concentration, usually by colorimetric assays like BCA. However, data on total protein yield were mostly lacking. Total protein assays do not distinguish EV and non-EV proteins, i.e., sEV preparation purity 150. As a result, the MISEV2018 guidelines advocate assessing protein-to-particle, lipid-to-particle, or lipid-to-protein ratios to indicate sample purity, as well as negative or depleted markers. Since ratios and negative markers were rarely documented in the reviewed research, the purity of the tested preparations cannot be assessed. It is important to note that co-isolated impurities should not be confused with the extravesicular cargo or loosely-associated factors 17, which may contribute to EV bioactivity 151-153, although the distinction between the two is still being defined 17.

Interestingly, our meta-analysis revealed a lower heterogeneity index for the wound closure rate outcome for the studies that adhered to MISEV2018. However, the reviewed articles emphasized “positive” EV markers, particularly tetraspanins (CD63, CD9, and CD81), over cytosolic markers (TSG101, Alix, and HSP70). While tetraspanins may be functional 154, their involvement in sEV-mediated skin wound healing is still largely unknown. Western blot was the preferred approach in these studies due to its utility for bulk analysis and protein marker identification, but it is less helpful to analyze EV subpopulations or to understand marker allocation within positive subpopulations 150. Single-particle approaches like flow cytometry were less popular here. Morphology was assessed mainly by TEM. Collectively, these findings are consistent with previous reports, in that these analysis techniques remain the most favorable by the EV community 155; however, expanded approaches are needed to understand the contributions of EV subtypes and non-EV components.

Encouragingly, opportunities for advancement abound. Despite the unique signature of lipids in EVs, they were rarely characterized in the covered studies. The current state of knowledge on the structure and function of EV lipids is still rudimentary 156. EV lipids may play a part in mediating EV bioactivity 157, including during senescence induction 158. Here, only a single study identified sEV lipids as key players in the mechanism of sEV-mediated stimulation of wound regeneration 83, highlighting the need for more investigation. Similarly, standardized reference materials are needed to facilitate benchmarking. Nanoparticles and EVs are being engineered or recombinantly synthesized to allow calibration, validation, and quality control 150. As EV research advances, characterization technologies are also being applied in novel ways to EVs: for example, ion-mobility spectrometry (IMS), Raman spectroscopy (RS), and nano-flow cytometry (Nano-FCM) 159.

#### Dosage regimen

Optimization of dosing (method of delivery, amount per administration, frequency, interval between doses, and duration of the intervention 160) is crucial to achieve full therapeutic potential while mitigating off-target effects. For wound healing and skin regeneration, the optimal sEV dosage regimen has yet to be established.

##### Methods of administration

In most studies we reviewed, sEVs were directly infused to the wound site, primarily via injection, and to a lesser extent by direct application after incorporation into biomaterial scaffolds. Local delivery of sEVs has several advantages, circumventing phagocyte and circulatory clearance (primarily in the liver and spleen 126) while increasing bioavailability in target tissues and reducing the required therapeutic dose. sEVs were found to exhibit tropism for specific organs (lung, liver, kidney, spleen) when delivered to the systemic circulation 161, but the single study that compared sEV intravenous (IV) with subcutaneous (SC) administration produced an unexpected finding: IV-administered sEVs promoted skin regeneration more effectively than SC-administered equivalents, homing to the wound bed starting from day one of treatment 94. While prior studies suggested preferential affinity of sEVs for injured tissues 162, the reason for the observed variation in therapeutic impact between IV and SC requires additional investigation.

Non-healing lesions requiring protracted therapy may have limited clinical translatability because of patient compliance. Incorporating sEVs into biomaterial scaffolds might reduce the frequency of application and enable delivery of sEVs in a targeted and concentrated manner. Here, hydrogels (both synthetic and natural) were the most preferred biomaterials. In these biomaterials, EVs are shielded against rapid clearance and destruction in the hostile wound environment, prolonging their duration of release and bioavailability 44. Hydrogel dressings are typically used to rehydrate the injured tissues, insulate against infections, and provide a temporary framework for host cells to penetrate and adhere to before being replaced by native ECM 163, or for their antibacterial qualities 164. Combining these qualities with the proangiogenic, pro-regenerative, anti-inflammatory, and anti-scarring features of sEVs was beneficial. Several studies in this review utilized “smart” biomaterials: temperature, light, and pH-responsive, enabling spatially and temporally orchestrated release of sEVs. Light-triggerable hydrogels loaded with sEVs accelerated wound closure better than non-triggered hydrogels or even several doses of sEVs, underscoring the critical role of the controlled release system in boosting healing kinetics 44. Advances in 3D bioprinting will enable scaffolds to be tailored to each patient's unique demands. It is critical, however, to investigate the possible interaction of biomaterials with sEVs, and their effect on the functionality and physical attributes of the vesicles.

##### Dose and frequency

To date, there are no guidelines for EV dose selection. Here, experimental doses ranged from 2 to 5000 µg of protein. In most studies, dose selection was unclear and did not account for wound size, condition, type of injury, skin type, animal weight, or route of administration. Since protein levels are influenced by purity, dosage estimation may also be unduly influenced by impurities, which were mostly unreported. Dose-response studies were also lacking, and dose was based only on *in vitro* experiments in 2D in some studies, although the dose needed *in vivo* to present the therapeutic benefits may differ substantially from that projected *in vitro* 16. Two studies concluded that sequential administration of small doses was superior to a single administration of a larger dose 44, 82. Furthermore, sustained release from a light-responsive biomaterial scaffold outperformed high-dose and sequential administration regimens [CITE]. Both head-to-head comparisons and quantifiable measures of potency and quantity of biotherapeutic molecules in EVs are needed to establish dosage 165.

#### Labeling and tracking of sEVs

Despite the importance of *in vivo* stability, only six studies tracked transplanted sEVs (Table 4). In these studies, sEVs were found to be localized to the wound bed and were detected in the cytoplasm of skin tissue cells. However, the wide range of follow-up time points and duration hampered comparability and conclusions about how long a single sEV dose remained detectable in tissue. While one study observed that sEV signals lasted up to 21 days (IV delivery) 94, another noted that they disappeared after only five days 87. Sjöqvist *et al*. reported a loss of signal four days after injection due to interference by hair growth and scab formation; however, the signal could be detected later after extraction of tissue (*ex vivo*) 82. Another study noted that the drop in sEV detection signal was less pronounced for light-triggered hydrogels than for free sEVs, pointing, as previously highlighted, to the potential of sustained-release systems in prolonging sEV bioavailability.

Accelerated clearance of systemically introduced sEVs after repeated administration has been reported in the literature, probably due to development of immune responses 166. Further investigation is needed to determine if this happens in skin tissues. Additionally, the depth to which sEVs penetrated damaged tissues was not investigated. When Zhang *et al*. assessed the potential of sEVs to permeate an *ex vivo* model of intact human skin, they noticed that sEVs could not penetrate beyond the stratum corneum of the epidermis, proposing it as a possible area of activity 167. However, sEVs would plausibly penetrate deeper in skin injury versus intact skin because of barrier disruption. This should be tested in skin injury.

Beyond general dose and tracking questions, we believe that upcoming studies should address fundamental questions including: 1) What proportion of sEVs are internalized by cells? What are the target cells? Is internalization required to exert the therapeutic effect? How about surface ligand-receptor interactions? Does uptake of sEVs necessarily mean functional delivery of payloads (membrane-membrane fusion)? Are sEVs similarly internalized by different types of cells in the skin, or do they show different tropisms? What is the influence of cell source or various preparation techniques on sEV uptake and tissue distribution?

Importantly, sEVs were predominantly labeled with fluorescent lipophilic dyes in the analyzed studies. These dyes sequester into the lipid areas of the phospholipid membranes and are highly photostable 168. As this is a widespread approach for *in vivo* tracking of labeled cells with negligible alteration of functional and mechanical properties of cells 168, this approach may not be preferable for tracking sEVs due to the lack of specificity. Unfortunately, lipid dyes also label cellular membranes 168, and some have been found to have low EV staining efficiency 169, bind to non-vesicular impurities such lipoproteins and soluble proteins 170, or aggregate into nano-sized micelles that can be confused with sEVs 130, 169. They could also leak from EVs and persist in tissues, greatly outlasting the labeled EVs *in vivo* due to long half-life, causing misinterpretation of EV fate *in vivo* 171. This lack of specific labels makes tracking EVs a challenging task. New labeling techniques with improved performance are emerging, such as radiolabeling 172 and magnetic labeling 173, yet all with pros and cons, mandating use of complementary technologies for a better understanding of EV distribution in tissues 130.

#### Relevance of animal models and external validity aspects

The studies here investigated rodent models for sEV efficacy (97% of studies), including diabetic and non-diabetic mice and rats. Type 1 diabetes was predominantly modeled, while type 2 diabetes, the most prevalent of the two, was underrepresented. Similarly, full-thickness excisional wounds were assessed more frequently than other lesions, such as diabetic foot ulcers, pressure ulcers, incisional or partial thickness wounds, and burns.

Although rodent models have been crucial in the initial stages of new drug screening and testing, inherent structural and physiological differences between rodent and human skin have spurred heated arguments on the translational relevance of these models 174. Indeed, 90-95% of drugs that show promise in pre-clinical trials fail to translate to the clinic 175. Despite similarities in portraying the four overlapping phases of wound healing, variances in life expectancy, the number of skin layers and thickness, and healing mechanism (e.g., contraction-based) are among the many factors that might undermine clinical applicability 174. Furthermore, the extent to which existing diabetes models accurately represent chronic, non-healing wounds remains an open subject 3. For example, in the reviewed studies, diabetic wounds in control groups were capable of independent healing. Also, relevant non-healing wound scenarios such as infected wounds or aged-animal wounds were understudied. Collectively, these concerns challenge the “external validity” of the studies, that is, the degree to which the outcomes can be generalized to human disease 176.

Since each strategy has limitations, we recommend a combination of approaches. Apart from rodent models, which have already demonstrated significant success in promoting cutaneous regeneration, larger animal models such as swine and non-human primates might be important to study. Together, evidence from diverse species might better predict human response 177. Supporting data from emerging approaches that aim to achieve the 3R principles (replacement, reduction, refinement) for animal experiments are also valuable. This includes evidence from technologies such as microfluidic tissues-on-a chip that involve multiple system interactions and enable real-time mechanistic readouts 178. Other approaches include in silico experiments, artificial intelligence, and machine learning 179. Additionally, more representation of lesions such as burns and diabetic ulcers is needed. Consistent evaluation of the testing system's predictability and applicability is critical for determining the effectiveness of each approach.

#### Internal validity aspects

Successful translation requires not only external, but also “internal validity" 176: the rigor with which experiments are designed, conducted, and reported 176. Inadequate reporting impedes assessing experiment validity and is a common issue in preclinical research 180. In some domains, the scientific community has developed guidelines and checklists of a minimum set of items to be documented in comprehensive and transparent reporting, such as the MISEV2018 recommendations 14, EV-TRACK, and the MSC-EV criteria mentioned previously. Compliance with these guidelines has been correlated with enhanced reporting, experimental design, and conduct 181, 182. Here, we observed that incomplete reporting affected many aspects of research design and methodology. For instance, there was an overall poor reporting of pre-processing and EV source, the number and age of animals, how sample size was determined, and of numerical data for means or variation measures for the outcomes. In some cases, experiments were reported that were not covered in the method section. Only a single study submitted procedures to EV-TRACK 82. This incomplete reporting limited our ability to examine key elements that are identified by the SYRCLE's ROB tool as plausible sources of bias in animal experiments 95. As a result, we concluded an unclear risk of bias for most of the elements (Figure 6), presenting reasonable grounds for concern about the true level of bias in the included studies. Failing to handle internal validity threats is likely a key contributor to overestimated effect sizes in preclinical research 183. Adoption and documentation of better reporting measures would help to mitigate or identify bias and increase confidence in the evidence and quality of the experiments 184. The non-mandatory nature of the reporting guidelines may explain their low uptake in published studies. While authors should strive to provide detailed experiment reports, journals and funding agencies are the gatekeepers and should endorse compliance with reporting guidelines 180.

#### Is sEV intervention a safe option in skin regeneration therapy?

The possibility of adverse reactions to sEV therapy received scant attention, although the few studies that examined this possibility detected no serious adverse effects. Interestingly, no reports indicated immune rejection even of allogeneic or xenogeneic preparations, which constituted the majority of the tested preparations. Of note, Lu *et al*. observed a mild immune response to allogeneic iPSCs but not to their sEVs, although allogeneic sEV viability in treated tissues was inferior to that of their autologous counterparts 42. These findings collectively are in line with prior research supporting sEV safety in treating various conditions 20. Although hundreds of animal studies and clinical trials have demonstrated the biosafety profile of sEVs, safety assessment is integral to any novel therapeutic product development. As emphasized earlier, each EV preparation is a unique product due to the heterogeneous components involved in its manufacture 139. In the absence of guidelines by regulatory bodies to assess the associated risks, verification of each product's safety is necessary 29. This entails evaluating the potential risks associated with each stage, from source selection to delivery to patients 185.

Given that sEVs are messengers for their parent cells, caution is needed in source selection. While MSCs have an established general safety profile 20, 129, sEV therapies are derived from various sources, not just from MSCs. Some of these sources have unclear safety profiles. Earlier studies in the literature showed examples of immune responses to immune cell-derived-EVs, for instance, when delivered to patients 186. Additionally, immortalized cell lines and cancer cells may pass along oncogenic components through EVs 141, 187. EV-associated extravesicular cargo 17 such as protein coronae may also elicit immune responses, which should be considered 188.

As discussed earlier, adverse effects associated with manipulation of source cells or EVs should also be assessed. For instance, extending EV half-life might have the potential to induce fibrosis. Additionally, the risks associated with recurring sEV administration, overdosing, and off-target interactions merit further examination. An improved understanding of sEV biodistribution and bioavailability in treated tissues will surely aid in deciphering these interactions 141. It is also imperative that regulatory approval is obtained before the initiation of clinical trials and application in clinics to confirm safety and efficacy 29. Unlicensed “exosome”-containing products that might result in devastating consequences for patients led the FDA to issue a “Public Safety Notification on Exosome Products” 189, stressing the need for regulatory approval to ensure safety and efficacy before initiating clinical trials and application in clinics 29.

## Concluding remarks and future prospects

Providing effective therapeutic options to promote skin regeneration is a key goal that has yet to be met, placing a continual burden on public health. This health burden is projected to continue to grow as diabetic and elderly populations expand. sEV therapy is proposed as a promising biological therapeutic approach that is able to potentiate skin regeneration. In this review, we have systematically examined studies evaluating sEV potential to induce wound healing and skin regeneration. We critically analyzed findings, reporting, and methodology and discussed strengths, pitfalls, and challenges. Collectively, our critical review of the available preclinical evidence supports the therapeutic efficacy and safety of sEVs in cutaneous wound healing and skin regeneration across all outcomes (wound closure, angiogenesis, anti-inflammation, re-epithelialization, scarring reduction, collagen production), regardless of cell source, production protocol, or disease model. Modifying sEV cargo appears to potentiate therapeutic functionality, resulting in better healing compared with unmodified sEVs. Moreover, biomaterial scaffolds could offer a promising tool for targeted sEV delivery.

However, these exciting findings should be interpreted with caution for several reasons. First, most studies have unclear risk of bias, and insufficient reporting hampered our ability to make an informed judgment. Second, the absence of purity assessment makes it difficult to attribute the observed efficacy to sEVs versus co-isolates. Third, the animal models used may not be faithful representatives of human disease, although this is a general limitation of animal testing and not unique to sEVs. Lastly, heterogeneity in sEV production, sources, characterization, and dosage regimens challenge direct comparisons.

We believe that adherence to established guidelines such as MISEV2018 will facilitate the conduct of rigorous research and enhance its validity. The high failure rate in translating preclinical discoveries to the clinic represents a waste of time, effort, and resources. Therefore, applying rigor and analyzing risks from the early stages of development will largely contribute to greater translatability and success stories. As a result, we urge researchers to follow best reporting practices, including a detailed description of their rigorous work in order to enable reproducibility, comparability, and evaluation of the studies' evidence. Submission to EV-TRACK is a great approach to enhance transparency and may also serve as a guide for authors regarding which items should be reported. To account for the shortcomings of confining research to rodent models, we propose examining a variety of models, including non-rodent and non-animal models. We further recommend that the follow-up period be extended to adequately assess the effect of sEVs on the healed skin maturation and scarring, as well as the immunological response. Future research should cover other skin lesions as well, such as burns, ulcers, incisional ischemic lesions, and more representative models of diabetic type 2 and non-healing wounds.

Moreover, there are several unresolved issues that warrant future research. These include establishing the optimal sEV source with the minimal possible risks and greatest therapeutic efficacy as well as identifying the components responsible for the ascribed functions. In this regard, the evaluation and reporting of the preparations' purity should not be overlooked and should be regularly identified. Furthermore, more research is needed to establish the basis for dosage regimen selection to achieve the best outcome; and to evaluate the *in vivo* pharmacokinetics and pharmacodynamics of sEVs in skin tissues. Since the evaluation of possible unwanted effects has received less attention, it is critical for future research to conduct a full review of the safety aspects associated with sEV therapy of skin lesions. Comprehensive characterization is key in evaluating the role of EV cargo safety. Indeed, the variability of sEV production processes, sources, and characterization presents a challenge, however, the development of quality controls and suitable reference materials might provide the foundation for standardization, allowing for lab-to-lab comparability. Nonetheless, the current expansion of novel and intriguing approaches is encouraging. Also, personalized sEV skin therapy products might be the future direction as each skin type, condition, and defect may have different therapeutic demands.

The future for sEV skin therapies seems bright if we learn the right lessons. Along the way, decades of experience with enveloped viruses and synthetic nanoparticles, which have many similarities with sEVs, have much to teach the EV field, particularly regarding the former's large-scale production and purification and the latter's delivery. Safety and efficacy evaluation of biologics (such as cell therapy) may also serve as reference models for EV therapy. However, clinical applications should not be hastened prior to clinical trials and regulatory permissions, as this will put patients at risk and may jeopardize the field. Although the road to the clinic is still paved with obstacles, sEV therapy holds tremendous potential as a biological cell-free therapeutic modality capable of promoting wound healing and skin regeneration. Collaborative efforts are needed to realize this potential and successfully translate it into practice.

## Methodology

### Literature search strategy

The protocol of this study was developed a priori, peer-reviewed, registered, and published in the International Prospective Register of Systematic Reviews (PROPSPERO; protocol ID: CRD42020159994). The current review covers only the *in vivo* part of the intended study. The search was initiated by forming a query (i.e., keywords) sourced from a number of related published studies and the Medical Subject Headings (MeSH) Database. The formed query was further checked by experts in the field (JX.L., M.A.A., JB.F.). Three bibliographic databases containing peer-reviewed journals were searched, namely, Web of Science (Science Citation Index Expanded), Scopus, and PubMed. Where applicable, filters were applied to include only original research publications, that were written in English and had the search terms in the title and abstract. No restriction on the date was applied. All returned articles were pooled from the three databases to EndNote, and duplicates were removed. Initially, the search covered studies that were published until 11^th^ November 2019. Later, the search was updated to include related studies published from 11^th^ November 2019 to 1^st^ March 2021, obtained through an activated search-alert that was set earlier. Study selection was carried out at two stages by two reviewers independently (M.E.A., J.X.L.). Disagreements were resolved by discussion and by consulting the third and fourth reviewers (M.A.A. and J.B.F.). The first search stage involved screening titles and abstracts of retrieved studies, while the second stage was based on reviewing the full text of articles according to the predefined exclusion and inclusion criteria. The search query was adjusted as needed to function with each respective database. Keywords used were as follows:

Web of Science: (TS= (exosome* OR “extracellular vesicle*” OR nanovesicle*) AND TS=(“skin regeneration” OR “skin rejuvenation” OR “wound healing” OR “skin repair”))* AND* LANGUAGE: (English) *AND* DOCUMENT TYPES: (Article).

PubMed: ((exosome* OR extracellular vesicle* OR nanovesicle* AND (English[lang]))) AND (wound healing OR skin repair OR skin regeneration OR skin rejuvenation AND (English[lang]))

Scopus: TITLE-ABS-KEY ((exosome* OR “extracellular vesicle*” OR nanovesicle*) AND (“skin regeneration” OR “skin rejuvenation” OR “wound healing” OR “skin repair”)) AND DOCTYPE( ar ).

### Eligibility criteria

For the qualitative synthesis of studies, at the first stage we included only those articles that were 1) peer-reviewed original research articles; 2) written in English; 3) evaluated exosome/sEV therapeutic roles in wound healing and skin regeneration; and 4) conducted in mammalian animal models. Human trials were not covered in this review. We excluded studies that 1) were not original research, such as reviews, letters, commentaries, and conference proceedings, as well as those that were non-peer-reviewed (including preprints); 2) were written in languages other than English; 3) were unrelated to sEV applications in wound healing; and 4) were on non-mammalian animal models.

At the second stage of search, only studies that had 1) controlled interventional design; 2) examined at least one sEV positive marker (of the markers recommended in MISEV2018 14); 3) experimentally confirmed the size of isolated sEVs (30-200nm); 4) investigated wound healing and skin regeneration either macroscopically or microscopically, qualitatively or quantitatively; and 5) of which full text could be accessed either online or after request from the authors. There were no restrictions on sEV source, source cell manipulation, scaffold or vehicle use, or control type. We excluded studies that: 1) did not establish sEV identity by determining the size; 2) did not establish sEV identity using at least one EV positive protein marker; 3) did not assess wound healing or skin regeneration macroscopically or microscopically, qualitatively or quantitatively; 4) were not controlled; 5) did not include the pre-specified primary outcomes or reported insufficient data on the outcomes; or 6) their full text could not be retrieved despite contacting the authors.

### Data extraction and synthesis

Data were extracted by two independent groups of reviewers (M.E.A and CY.N. as well as U.V. and R.S.) and arranged in a pre-designed data extraction form prepared by the reviewers. Disagreements were resolved by discussion and by consulting the third and fourth reviewers (JX.L. and M.A.A.). Data were extracted from texts, tables, figures, supplementary materials and references cited for methods. These included intervention design, dosage (amount), dosing frequency, route of administration, dose-response assessment, biocompatibility, vehicles, and comparators; characteristics of the animal models used, including species/strain, gender, age, body weight, disease model, wound model, size, and location; as well as the number of animals per group. Data on the EV source cells were also collected including cell type, modification type, if any, cell passage at extraction. Also, data on exogenous EV depletion regimens, methods of sEV separation, and characterization. In addition, any data on labeling and tracking of sEVs after administration were also collected. *The primary outcomes* extracted from studies were quantitative and qualitative data related to wound healing process evaluation, including wound closure rate; re-epithelialization; collagen deposition and maturation; angiogenesis; and scar assessment. *The secondary outcome data* extracted were qualitative data related to any reported adverse effects and inflammation. Additionally, general study characteristics such as authors, year, and country of corresponding author/s were obtained.

### Quality and risk of bias assessment

The Systematic Review Centre for Laboratory Animal Experimentation (SYRCLE's) risk of bias tool was used to assess each study's risk of bias 95. This tool comprises a ten item-checklist: (1) random sequence generation, (2) baseline characteristics, and (3) allocation concealment, to evaluate selection bias; (4) random housing and (5) researcher blinding, to evaluate performance bias; (6) random outcome assessment, and (7) blinding of outcome assessment, to evaluate detection bias; (8) incomplete outcome data, to evaluate attrition bias; (9) selective reporting, to evaluate reporting bias; and (10) other source(s) of bias, if any. We modified the tool to include another item, declaration of the randomization method, but we excluded point (10) and thus did not check for other sources of bias.

We further assessed sample size calculation, quality of reporting, adherence to MISEV2018 characterization criteria, nomenclature, purity assessment, and EV-TRACK submission 106. We also evaluated the adherence to ISCT minimal criteria to characterize mesenchymal stem cells (MSCs), where applicable. We evaluated the animal and disease models used to examine the external validity of the studies.

### Meta-Analysis

The studies identified through our comprehensive search were checked for eligibility for a meta-analysis. We performed meta-analysis for three outcomes: wound closure rate, scar reduction, and angiogenesis, using Review Manager 5.4.1 (Cochrane) 190, comparing naïve sEVs (unmodified) with placebo controls. Meta-analysis was performed only when three studies or more reported the same outcome using the same scale. We retrieved means, standard deviations, or standard error of the mean as stated in the studies and emailed the authors to obtain missing data (including sample size), where necessary. We extracted data from figures in studies where numerical data were not available using an online application (WebPlotDigitizer, https://apps.automeris.io/wpd/). We excluded from the meta-analysis the studies for which the sample size was not provided even after contacting the authors. Standard mean differences (SMD) and pooled size effects were estimated using random effects model, taking into account the diversity in sEV preparation, source, and wound size. Statistical heterogeneity (a measure of the heterogeneity of intervention effects across studies) was measured using I^2^ index. A subgroup analysis of naïve sEVs versus placebo control was performed to assess if the effect of sEVs varied between diabetes and non-diabetes models. Additionally, we conducted another meta-analysis of wound closure rates for studies that reported characterization using MISEV2018 criteria, with subgroup analysis of diabetes and non-diabetes models. Statistical significance was considered when *p* < 0.05. To note, outcomes of studies that were not included in the meta-analysis were described qualitatively.

## Figures and Tables

**Table 1:** Summary of the methods used for separation and characterization of small extracellular vesicle used by the reviewed studies for treatment of wounds in animal models

Ref	Source	Collection Medium Supplementation	Isolation	Characterization	EV characteristics		
					Size Markers	Morphology	Detected
52	Primary human fibrocyte (preconditioned with PDGF-BB, TGFβ1, FGF2, ITS)	5% exosome depleted FBS, 48 h	Ultrafiltration	-Protein concentration: BCA assay;-Morphology: TEM;-Size distribution and concentration: NTA;sEV markers -Western blot TSG101, flotillin-1, GM130, calnexin -Flow cytometry; CD9, CD63, CD81	50-100 nm	Cup-shaped	Positive for: CD9, CD63, CD81, TSG101, and flotillin-1; Negative for: GM130 and calnexin
50	Primary hUC-MSCs (preconditioned with 100ng/ml LPS)	Serum free medium, 48 h	-Filtration: 0.22µm filter; -Centrifugation: 10,000×g for 30min;-Ultracentrifugation: 100,000×g for 3h	-Protein concentration: BCA assay;-Morphology and size: TEM;sEV markers: Western blot (CD9, CD63, CD81)	40 - 90 nm	Cup-shaped	Positive for: CD9, CD63, CD81
45	Primary hUC-MSCs and HFL1	Serum free medium (DMEM)	-Differential centrifugation: 1000×g for 20min, 2000g for 20min, 10,000×g for 20 min;-Ultrafiltration/Concentration: 100kDa filter at 1000×g for 30min;-Density gradient centrifugation: 100,000×g for 60 min in 30% sucrose-D2O cushion. -Washed (x3) in PBS at 1000g for 30 min in 100kDa MWCO filter; -Filtration: 0.22µm filter.	-Protein concentration: BCA assay;-Morphology: TEM; -Size distribution and concentration: NTA; -sEV markers: Western blot; CD63, CD9, CD81	100nm	Spherical vesicle	Positive for: CD63, CD9, CD81 (both hUC-MSC and HFL1 derived exosomes)
98	hu-iPSC-derived MSCs	Chemically defined medium, 48 h	-Differential centrifugation: 300××g for 10min, 2000×g for 10min; -Filtration: 0.22µm filter; -Ultracentrifugation: 100,000×g for 2h; -Ultrafiltration/Concentration: at 4000×g	-Protein concentration: BCA assay; -Morphology: TEM; sEV markers: Western blot, CD63, CD9, CD81	30-100 nm	Spheroidal	Positive for: CD63, CD9, CD81
73	Primary hUC-MSCs	Exosome depleted FBS (UCG : 120,000g for 3h)	-Differential centrifugation: 300×g for 10 min, 16,500×g for 20 min; -Filtration: 0.22µm filter; -Ultracentrifugation: 120,000×g for 70 min	-Protein concentration: BCA assay; -Size distribution and concentration: NTA; -sEV markers: Western blot CD81, CD63; -microRNAs profiling: HTC	55nm	NS/NR	Positive for: CD81, CD63
94	Primary hADMSC (subcutaneous fat)	Serum-free medium, 24h	-Centrifugation: 3,000×g for 15 min; -Filtration: 0.22µm filter; -Ultrafiltration: 100kDa MWCO filter; -Precipitation: Exosome Precipitation (kit); -Filtration: 0.22µm filter	-Protein concentration: BCA assay; -Morphology: TEM; -Size distribution and concentration: NTA; -sEV markers: Western blot CD63, CD9, tubulin and lamin A/C	30-100 nm (85%)	Cup-shaped	Positive for CD63, CD9; Negative for: Tubulin and lamin A/C
60	Primary SMSCs-126 (transfected with miR-126-3p)	Chemically defined medium, 48 h	-Centrifugation: 2,000×g for 30 min; -Filtration: 0.22µm filter; -Ultrafiltration: at 4000××g for 30 min; -Washing with DPBS: 4000××g for 30 min; -Density gradient centrifugation: 100,000×g for 60 min in 30% sucrose-D_2_O cushion; -Washing with PBS: 4000××g for 30 min	-Morphology: TEM; -Size distribution: DLS; sEV markers: Western blot CD9, CD63, CD81, and TSG101	85 nm	Spherical	Positive for: CD9, CD63, CD81, and TSG101
62	Primary human SMSCs-126 (transfected with miR-126-3p)	Chemically defined medium, 48 h	-Centrifugation: 300×g for 15min, 2,000×g for 15 min; -Filtration: 0.22µm filter; -Ultrafiltration: at 4000×g; -Washing pellet in PBS -Ultrafiltration: at 4000×g; -Density gradient centrifugation: 100,000×g for 60 min in 30% sucrose-D_2_O cushion; -Washing pellet in PBS: at 4000×g	-Morphology: TEM; -Size distribution: DLS; -sEV markers: Western blot CD9, CD63, CD81, and TSG101, Alix.	30-150 nm	Spherical	Positive for: CD9, CD63, CD81, and TSG101, Alix
93	Primary hUCB-EPCs	Deprived medium of FBS+ 1 ×serum replacement solution, 24h	-Differential centrifugation: 300×g for 10min, 2000g for 10min; -Filtration: 0.22µm filter; -Ultrafiltration/Concentration: at 4000×g; -Washed (x2) in PBS; -Ultrafiltration/Concentration: at 4000×g; -Density gradient centrifugation: 100,000×g for 60 min in 30% sucrose-D2O cushion; -Ultrafiltration/Concentration: at 4000×g	-Morphology: TEM; -Size distribution and concentration: TRPS; -sEV markers: Western blot CD63, CD9, CD81, epithelial marker CD31	50-60 nm	Cup- or round-shaped	Positive for: CD63, CD9, CD81, EPC marker CD31
105	Primary hADMSCs	Serum-free DMEM, 48 h	-Differential centrifugation: 300×g for 7min, 1000×g for 15 min, 10,000×g for 40 min, 15 min at 1000 ×g; -Ultracentrifugation: 100,000×g for 70 min (x2)	-Protein concentration: Bradford method;-Size distribution and concentration: NTA; -sEV markers: Western blot (Alix and CD9)	135 nm	Ns/NR	Positive for: Alix and CD9
80	hPRP (freshly isolated)	NA	- PRP centrifuged at 250 × g for 15 min; -Pellet washed (3x) with PBS; Pellet activated; 300×g for 10 min; 2,000×g for 10 min; -Filtration: 0.22µm filter; -Ultrafiltration: at 4000×g; -Washing pellet in PBS (3x); -Ultrafiltration: at 4000×g; -Density gradient UG: 100,000×g for 70 min in 30% sucrose-D2O cushion; -Washed in PBS at 100,000×g for 70 min	-Morphology: TEM;-Size distribution and concentration: DLS; -sEV markers: Western blot CD9, CD63, CD81, and the source marker CD41, VEGF, TGFb1, bFGF, PDGFB	40-100 nm	Cup- or sphere-shaped	Positive for: CD9, CD63 and CD81, CD41 (platelet marker) VEGF, TGFb1, bFGF, and PDGFB
76	Primary hGMSCs	10% exosome-free FBS, 48h	-Centrifugation; -Filtration: 0.22µm filter; -Ultrafiltration: 30kDa filter at 5000×g for 30min; -Size exclusion chromatography; -Ultrafiltration: 30kDa filter at 5000×g for 30min	-Protein concentration: BCA assay; -Morphology: TEM; -Size distribution and concentration: TRPS; -sEV markers: Western blot (CD9 and CD81)	127 ± 55.9 nm	Spherical	Positive for: CD9 and CD81
109	Primary hADMSC (subcutaneous)	Serum free medium, 24h	-Centrifugation: 3000×g for 15min; -Ultrafiltration: 100kDa filter; -Filtration: 0.22µm filter; -Precipitation: Exosome Precipitation (kit) at 1500×g for 30min; -Filtration: 0.22µm filter	-Protein concentration: BCA assay; -Morphology: TEM; -Size distribution and concentration: NTA; -sEV markers: Western blot (CD63, CD9)	NR	NR	Positive for: CD9, CD63
91	Primary hAECs	10% exosome-free FBS medium	-Centrifugation: 300×g for 5min; -Filtration: 0.22µm filter; -Ultracentrifugation: 100,000×g for 12h; -Ultrafiltration/Concentration: at 4000×g	-Size distribution, morphology: SEM; -sEV markers: Western blot CD9, CD63, Alix and TSG101; Flow cytometry CD9, CD63, CD81 and HLA-G	50-150 nm	Round or oval	-Western blot: Positive for CD9, CD63, Alix and TSG101; - Flow cytometry: Positive for CD9 (88.8 ± 6.1%), CD63 (98.1 ± 1.2%), CD81 (91.7±3.6%) and HLA-G (95.6±3.4%).
84	Primary hUSCs (transfected with shRNA DMBT1)	Exosome free FBS, 48h	-Differential centrifugation: 300×g for 10min, 2000g for 30min, 10,000 ×g for 30 min; -Filtration: 0.22µm filter; -Ultrafiltration: 100kDa filter at 4000×g; -Washed (x2) w PBP at 4000 ×g; -Precipitation: Exosome precipitation (kit) at 1500 ×g for 30min	-Protein concentration: BCA assay; -Morphology: TEM; -sEV markers: Western blot (CD9, CD63, CD81 and TSG101); -Flow cytometry (CD63, TSG101); -Proteomic analysis: TMT labeling, HPLC fractionation, and LC-MS/MS	51.57 ± 2.93 nm	Cup- or sphere-shaped	Positive for CD9, CD63, CD81 and TSG101
87	Primary hUCBP	NA	-Differential centrifugation: 300×g for 10 min, 2,000×g for 20 min, 10,000×g for 30 min; -Ultracentrifugation: 100,000×g for 70 min; -Washing pellet in PBS (2x); -Ultracentrifugation: 100,000×g for 70 min; -Filtration: 0.22µm filter; -Ultrafiltration: at 4000×g	-Morphology: TEM; -Size distribution and concentration: DLS; -sEV markers: Flow cytometry CD63, TSG101	30-100nm	Cup-shaped or spherical	Positive for: CD63, TSG101
97	Human iPSCs (cell line 201B7)	Cultured in serum free medium (KnockOut Serum Replacement)	Exosome Isolation Kit	-Morphology: TEM; -sEV markers: Flow cytometry; -CD63 or CD81, CD9, HLA-ABC, or HLA-DR	100nm	Spheroidal	Positive for: CD9, CD63, and CD81; Negative for: HLA-ABC and HLA-DR.
63	Primary hADMSCs and hADMSC-Nrf2	FBS-free EGM-2MV media+1× serum replacement solution, 24h.	-Differential centrifugation: 300×g for 10 min, 2,000×g for 20 min; -Precipitation: Exosome Precipitation (kit) at 1500×g for 30min	-Morphology: TEM; -Size distribution: DLS; -sEV markers: Western blot CD4, CD63, and TSG101, β-actin	100nm	Spherical	Positive for: CD4, CD63, and TSG101, negative for β-actin
96	Primary hMenSCs	Exosome free FBS, 48 h	-Differential centrifugation: 300×g for 10 min, 2,000×g for 20 min, 10,000×g for 30 min; -Ultracentrifugation: 100,000×g for 60 min	-Concentration: ELISA kit; -Morphology and size: FSEM, AFM; -sEV markers: Western blot (CD81 TSG101, calnexin).	40-200 nm	Spherical shape	Positive for: CD81 and TSG101 Negative for calnexin
54	Primary hBMMSCs (preconditioned with 200 µM DFO, 48h)	Exosome free FBS, 48h	-Differential centrifugation: 500×g for 10 min, 12,000×g for 20 min; -Filtration: 0.22µm filter; -Ultracentrifugation: 110,000×g for 70 min*;* -Washing pellet in PBS: UG for 110,000×g for 70 min	-Protein concentration: BCA assay; -Morphology: TEM; -Size distribution: TRPS; -sEV markers: Western blot CD9, CD63, TSG101, and GM130	50-150 nm	Cup-shaped	Positive for: CD9, CD63, TSG101; Negative for: GM130 (both Exos and DFO-Exo)
44	hUCBMNCs (Hypoxia 0.5% O_2_,18h)	Serum-free medium +Flt-3+stem-cell factor, 18h	-Differential centrifugation: 300×g for 10 min, 2,000×g for 20 min, 10,000×g for 30 min (2x); -Ultracentrifugation: 100,000×g for 120 min; -Washed in PBS at 100,000×g for 120 min	-Protein concentration: DC assay, BCA assay; -Morphology: TEM; -Size distribution and concentration: NTA, DLS; -sEV markers: Western blot CD63, GAPDH; Flow cytometry; TSG101, CD81, CD9, CD45 - RNAs profiling: HTC	100-130nm	Heterogeneous	Positive for: CD9, TSG101, CD63, and CD81, with low levels of CD45 (hematopoietic marker) and GAPDH
92	RAW 264.7 (mouse macrophage cell line)	heat-inactivated 10% FBS depleted of exosomes by UCG	-Centrifugation: 15,000 rpm for 30 min; -Filtration: 0.22-μm filter; -Ultracentrifuge at: 57,000 rpm for 1 h	-Morphology and size: TEM; -Size distribution and surface charge: DLS; - sEV markers: Western blot CD63 Alix, and β-actin	95 ± 9.9nm	Spherical	Positive for: CD63 and Alix positive for β-actin
42	Primary macaque -fibro-iPSCs	NR	-Differential centrifugation: 200×g for 10 min, 2,000×g for 20 min; -Ultrafiltration: 100Kda MWCO at 5000×g for 10 min; -Precipitation: Exosome Precipitation (kit); -Ultracentrifugation: 100,000×g for 70 min	-Size distribution and surface charge: NTA; -Morphology and size: TEM; - sEV markers: Western blot, Alix, and TSG101	100nm	Spherical (TEM)	Positive for: Alix, and TSG101
86	Mouse-Leukocyte-T BC1D3	NA	-Differential centrifugation: 300×g for 5min, 3,000×g for 20 min, 10,000×g; -Ultracentrifugation: 100,000×g for 70 min	-Size distribution and concentration: NTA; -sEV markers: Flow cytometry (CD9, CD63, and CD81)	125±23.8 nm (mode)	NR/NS	Positive for: CD9, CD81 and CD63
81	Primary Wistar Rat-ADMSC	NR	-Centrifugation: 10,000×g for 20 min; -Filtration: 0.22µm filter; -Ultracentrifugation: 120,000×g for 90 min	-Morphology: TEM; -Size distribution: SEM; -sEV markers: Western blot CD63	30-150 nm	Spherical, cup-shaped (TEM)	Positive for: CD63
82	Primary sOMECs (clinical grade sheets of oral mucosa epithelial cells)	5% autologous, serum	-Centrifugation: 300×g for 10min; -Filtration: 0.22µm filter; -Ultrafiltration: 100kDa filter Pooled; -Concentration: 10kDa filter, Size exclusion chromatography; -Concentration: 10kDa filter	- Protein concentration: BCA assay; -Morphology: TEM; -Size distribution and concentration: NTA; -sEV markers: Western blot (CD9, flotillin, Grp94, HSP70, EpCAM)	124.8 ± 4.1 nm	Spherical	-Positive for: CD9 and flotillin were positive -Negative for annexin V, HSP70, EpCam and contaminating marker Grp94
53	Primary hUCB-MSCs (preconditioned 10% O_2_, 40 U thrombin, 1 µg LPS, or 50 µM H_2_O_2_)	Serum free medium	-Centrifugation: 3,000×g for 30 min; -Ultracentrifugation: 100,000×g for 120 min, Washed (x2) w PBP	-Protein concentration: Bradford; -Morphology: TEM, SEM; -Size distribution and concentration: NTA, DLS; -Single size count: (LUNA-FL); -sEV markers: Western blot (CD9, CD63, CD81 Cytochrome C, GM130 and fibrillarin)	30-100 nm	Round shape	-Positive for: CD9, CD63, CD81; -Negative for: GM130 and fibrillarin; -Positive in H_2_O_2_- and hypoxia-preconditioned sEVs but negative in naïve sEVs or the thrombin or LPS-preconditioned sEVs: Mitochondrial Cytochrome C
70	Primary mouse-ADMSC (4wk old mice, epididymis-fat derived)	NR	-Centrifugation: 800×g for 5min, 2000×g for 10min; -Filtration: 0.22µm filter; -Ultrafiltration: 100kDa filter; -Ultracentrifugation: 100,000×g for 90min	-Protein concentration: BCA assay; -Morphology: TEM; -Size distribution and concentration: DLS; -sEV markers: Western blot (CD63, CD81, CD9, Alix)	60-80nm	Cup-round shaped	Positive for: CD9, CD63, CD81, Alix
71	Primary mouse-ADMSC (4wk old mice, epididymis-fat derived)	NR	-Centrifugation: 800×g for 5min, 2000×g for 10min; -Filtration: 0.22µm filter; -Ultrafiltration: 100kDa filter; -Ultracentrifugation: 100,000×g for 90min	-Protein concentration: BCA assay; -Morphology: TEM; -Size distribution and concentration: DLS; -sEV markers: Western blot	200nm	NR	NR
108	Primary hFDMSCs (human fetus skin)	Serum free medium, 48h	-Centrifugation: 3000×g for 15min; -Filtration: 0.22µm filter, Ultrafiltration; -Precipitation: Exosome Precipitation(kit) at 1500×g for 30min	-Protein concentration: BCA assay; -Morphology: TEM; -Size distribution and concentration: NTA; -sEV markers: Western blot CD63, Alix, TSG101	100nm	Cup-shaped	Positive for: CD63, Alix, TSG101
100	Primary hdMSC	10% exosome-free FBS	-Differential centrifugation: 300×g for 10min, 2000g for 10min, 10,000 ×g for 30 min; -Ultracentrifugation: 100,000×g for 70min; -Washed (x1) in PBS: 100,000×g for 70min	-Morphology: TEM; -Size distribution and concentration: NTA; -sEV markers: Western blot CD63, CD9, CD81, Grp94, TSG101	63.8 and 125 nm (90%, average = 94.4 nm)	Cup-shaped	-Western blot: Positive for CD63, CD9, CD81, TSG101 -Negative for: Grp94
102	Primary hUC-MSCs (transfected with w miR-27b-inhibitor)	EV-depleted FBS, 48 h.	-Centrifugation: R G force: to remove debris and apoptotic studies; -Ultracentrifugation: 110,000g for 70min; -Purification: 110,000g for 70min; -Filtration: 0.22µm filter	-Morphology: TEM; -Size distribution: NTA, DLS; -sEV markers: Western blot (CD63, CD81, TSG101, Calnexin)	30-100 nm	Cup-shaped or cystic-shaped	-Positive for: CD63, CD81 and TSG101; -Negative for: Calnexin
51	rat-AT, or p-AT (Adipose tissue extract)	NA	-Centrifugation: 2000 rpm for 20min: to remove cells and debris; -Filtration: 40µm filter, 0.22µm filter; -Concentration/Ultrafiltration: 3kDa MWCO, 5000g for 30min, Total Exosome Isolation overnight, 10,000g for 1 hr	- Protein concentration: BCA assay; -Morphology: TEM; -Particle size and size distribution: DLS; -sEV markers: Western blot (CD9, CD63, actin and TSG101)	Both samples: 80-200 nm (130nm)	Both had round shaped vesicles	-Positive in both: CD9, CD63, and TSG101 -CD9 at different molecular weights 25kDa vs 50kDa; -Negative for: Actin
74	hEPSCs (cell line)	Serum-free medium, 48h	-Filtration: 0.1µm filter; -Ultrafiltration: 100kDa MWCO; -Density gradient UG: 100,000×g for 70 min in 30% sucrose-D2O cushion, Washed in PBS at 1500 for 30min	- Protein concentration: BCA assay; -Morphology: TEM; - sEV markers: Western blot (CD9, CD63, GAPDH)	30-100nm	Round	-Positive in both: CD9 and CD63 negative for GAPDH
66	hAMSC (transfected with hAMSC-miR-135 OE; or hAMSC-miR-135 KD)	10% exosome depleted FBS, 48 h	-Centrifugation: 300g for 10 min; -Filtration: 0.22-μm filter; -Ultracentrifugation: 120,000g for 10 h.	-Protein concentration: BCA assay; -Morphology: TEM; -Particle size and size distribution: NTA; -sEV markers: Western blot (CD9, CD63, CD81, α-tubulin)	30-150 nm (103 nm)	Circular or elliptical in shape	Positive for CD9, CD63 and CD81, negative for α-tubulin
83	HS-5 (cell line)	Serum-free medium, 48 h	-Differential Centrifugation: Centrifuged at 4 °C and 2000 × g for 5 min; 10 000 × g for 15 min; -Filtration: 0.22-μm filter; -Ultracentrifugation: 100 000× g for 70 min	-Morphology: Cryo-TEM, TEM; -Particle size and size distribution: NTA; - sEV markers: Western blot (TSG101, CD9, CD63, Calregulin and CD73, GAPDH); -Proteomics Analysis: LC-MS; Lipidomic Analysis	89 ±7 nm	Round shaped	-Positive for: TSG101, CD9, CD63, and CD73; GAPDH; -Negative for: Calregulin
59	hBMMSCs (transfected to overexpress TSG-6)	Exosome-depleted FBS, 96h	-Centrifugation: 2000g for 30 min; -Filtration: 0.22-μm filter; -Total exosome isolation reagent: Incubation overnight + centrifuge at 10,000g for 1h	- Protein concentration: BCA assay; -Morphology and size: TEM; - sEV markers: Western blot (Alix, CD63, CD9 and TSG101)	20-100 nm	Cup-shaped	Positive for: Alix, CD63, CD9 and TSG101
67	Mouse-myeloid-MSCs	Serum-free, overnight	-Centrifugation: 2000g for 20 min; 10,000 g for 1 h; Suspended in serum free DMEM and 25 mM 2-[4-(2-hydroxyethyl)-1-piperazinyl] Ethanesulfonic acid (pH 7.4); 10,000 g for 1 h	-Size distribution and concentration: DLS; - sEV markers: Western blot; CD63, CD81, TSG101, heat shock protein 70 (HSP70), GRP94; Flow cytometry CD63; qRT-PCR: Expression of miR-152-3p	30-120 nm	Round or oval	-Positive for: CD63, CD81, TSG101, heat shock protein 70 (HSP70); -Negative for: GRP94
46	hUC-MSCs (loaded with 50 μg/mL of Fe3O4 NPs)	10% Exo-depleted FBS, 48h	-Centrifugation: 1500 rpm for 15 min; -Filtration: 0.22-μm filter; -Ultrafiltration: 100kDa MWCO filter; Membrane affinity spin column method (kit)	-Morphology and size: TEM; -Particle size and size distribution: NTA; - sEV markers: Western blot; CD9 and Alix	NTA: Exosomes only: 98.5±1.4 nm; Exosomes+NPs: 116.7±1.3 nm	Round, cup-shaped	Positive for Alix and CD9 proteins
48	Primary hADMSCs	Serum free media, 48h	-Differential centrifugation: 300 × g, 10 min; 10,000 × g, 60 min; -Filtration: 0.22µm filter; -Ultracentrifugation: 120,000 × g 2h; Washed (x2) with PBS and ultracentrifugation repeated	-Protein concentration: BCA assay; -Morphology: TEM; -Size distribution: NTA; -sEV markers: Flow cytometry (CD63, CD 81)	30-150 nm	Biconcave disc-shaped vesicles	Positive for CD63 and CD81
47	Primary hUC-MSCs (transfected with Lenti-Ang-2)	-Exosome free- FBS depleted medium (UC at 10,000g for 16h at 4°C), 48h	-Centrifugation: 2000g for 20 min; -Concentration/Ultrafiltration: 100 kDa MWCO at 1500×g for 30 min; -Filtration: 0.22µm filter; Overnight incubation with exosome isolation reagent and centrifugated at 1,500 × g for 15 min at 4 °C	-Protein concentration: BCA assay; -Morphology: TEM; -Size distribution and concentration: NTA; -sEV markers: Western blot (CD9, CD63, and CD81)	55nm	Spherical	Positive for: CD9, CD63, and CD81
55	Primary hBMMSCs (preconditioned with melatonin 1µmol/L, 48h)	Serum free medium, 48h	-Differential centrifugation: 300 × g/ 15 min; 2000 × g/ 20 min; -Filtration: 0.22µm filter; -Ultrafiltration: 100,000 × g/ 2 h (x2)	- Morphology: TEM; -Size distribution and concentration: NTA, sEV markers; Western blot: CD81, Tsg101, Alix and Calnexin	TEM: 120nm; NTA: 30-150 nm	Oval	Positive for: CD81, Tsg101, Alix and Negative for Calnexin
101	Primary hADMSC	EVs-depleted FBS and PL (70 000 *g* and 4°C overnight), 24-48h	-Differential centrifugation: 500 ×g for 10 min (2x); 2,000 ×g for15 min (2x); 10,000 ×g for 30 min (2); -Ultracentrifugation: 70,000 ×g for 1 h (2x)	-Protein concentration: BCA assay; -Morphology: TEM; -Size distribution and concentration: NTA; -sEV markers: Western blot (CD63, TSG101, calnexin)	30-100 nm	Cyathiform or spherical	-Positive for: CD63 and TSG101; -Negative for: endoplasmic reticulum marker calnexin
65	Primary hADMSC	Exosome free FBS medium, 48h	-Differential centrifugation: 400×g for 10 min; 2,000×g for 15 min; -Filtration: 0.22µm filter; -Ultrafiltration/concentration: 100kDa MWCO, 4000g; -Ultracentrifugation: 100,000×g for 70 min	-Protein concentration: BCA assay; -Morphology: TEM; -Size distribution: NTA; -sEV markers: Western blot (CD9, CD63, and TSG101)	41-130 nm (105.2 nm)	Spherical shape	Positive for: CD63, CD9, and TSG101
88	hsaliva‑Exos (unstimulated)	NA	-Differential centrifugation: 2,000 g for 30 min; 12,000 g for 45 min; -Filtration: 0.45 μm filter; -Ultracentrifugation: 110,000 g for 70 min; Washing in PBS and 110,000 g for 70 min	-Morphology: TEM; -Size distribution: Flow NanoAnalyzer, NanoFCM; sEV markers; Western blot (CD81, TSG101, Calnexin)	30-150 nm	Spherical	Positive for: CD81, Tsg101; Negative for: Calnexin
77	Primary hENSC	Exosome free serum, 24h	-Differential centrifugation: 300 × g for10 min; 2000 × g for10 min; 10000 × g for 30 min; -Ultracentrifugation: 100,000 × g for 70 min	-Morphology: TEM, SEM; -Size distribution: DLS; -sEV markers: Western blot CD63	40-150nm	Cup-shaped	Positive for CD63
85	mAPCs (3t311, cell line)	NM	-Differential centrifugation: 300 g for 10 minutes; 2000 g for 20 minutes; 10,000 g for 40 min; -Ultracentrifugation: 100,000 g for 120 minutes	-Protein concentration: BCA assay; -Morphology: TEM; -Size distribution: DLS; -sEV markers: Western blot (CD9, CD81, CD63, GAPDH, Hsp70)	30-300 nm	NR	-Positive for: Hsp70, CD9, CD63, and CD81, GAPDH
58	Mouse BMMSCs (pre-treated with either neonatal or adult serum exosomes)	FBS-free medium, 48h	-Differential centrifugation: 2000 g for 10 minutes; 10,000 g for 30 minutes; -Ultracentrifugation: 100 000 g for 70 min; -Washing in PBS: 100 000 g for 70 min	-Protein concentration: BCA assay; -Morphology: TEM; -Size distribution and concentration: DLS; -sEV markers: Western blot CD9, CD63, CD81 and TSG101	1-MSC-exosome: NR; 2-Serum exosomes: a) neonatal serum- 109.5 ± 2.1 nm; b) adult serum- 91.3 ± 2.3 nm	1-MSC-exosome NR; 2-Serum exosomes: spherical	1-MSC-exosome: NR; 2-Serum exosomes (neonatal and adult): -Positive for: CD9, CD63, CD81 and TSG101
56	Primary, HUVEC (preconditioned with PTHrP-2)	Serum-free medium, 48 h	-Centrifugation: 300g for 10 min; 2000 rpm for 15 min; -Filtration: 0.22-μm filter; -Ultracentrifugation: 100,000g for 1.5 h 2x	-Protein concentration: BCA assay; -Morphology and size: TEM; -sEVs size distribution: DLS; -sEVs concentration and size: FNA and NTA; -sEV markers: Western blot (CD9, TSG101and Alix)	40-100 nm (DLS)	Spherical or cup-shaped	Positive for Alix, CD9 and TSG101
68	Primary hADMSCs (transfected with mmu_circ_0000250)	FBS-free, EGM, 1% serum replacement solution, 48h	-Differential centrifugation: 300×g for 10 min; 2,000×g for 10 min; 10,000×g for 30 min; -Filtration: 0.22-μm filter; -Concentration/Ultrafiltration: 100kDa MWCO, 4000g and washed filter unit twice and filtered at 100,000 g	-Protein concentration: BCA assay; -Morphology and size: TEM; -sEV markers: Western blot CD81, CD63	50-120 nm	Cup-shaped or spherical	Positive for CD81, CD63
61	Mouse melanoma B16F10 (cell line, transfected with mouse PD-L1 Gene/ or stimulated with 100 ng/ml IFN-γ)	0.5% exosome-free FBS+ 1% P/S, 48h	-Differential centrifugation: 500 × g for 10 min; 2000 × g for 20 min; 10,000 × g for 40 min; -Ultracentrifugation: 100,000g for 90 min	-Protein concentration: BCA assay; -Morphology: TEM; -sEVs size distribution, zeta potential: DLS; -sEV markers: Western blot (CD63, CD81, Alix)	Peak at 100nm	Round-shaped and membrane-bound	Positive for CD81, CD63 and Alix
78	Primary hPMSCs	NR	-Filtration: 0.22-μm filter; Total Exosome Isolation Reagent overnight; -The mixture was centrifuged at 10,000g for 1 h; Ultrafiltration tube was centrifuged at 4000g at 4 °C	-Morphology: SEM; -sEVs size distribution, zeta potential: DLS; -sEV markers: Western blot (CD9, CD63, CD81)	62.2 nm	Round	Positive for CD9, CD63, CD 81
103	hPEC (from venous blood)	NA	-Centrifugation: EV precipitation solution; -Immunoprecipitation/enrichment: CD31 and CD146 monoclonal antibodies	-Protein concentration: BCA assay; -Morphology: TEM; -sEVs size distribution, concentration: NTA; -sEV markers: Western blot (CD63, CD81, TSG101, VCAM-1, GPVI)	123 ± 8 nm	Cup-shape, round	-Positive for: CD63, CD81, TSG101 and VCAM-1; -Low GPVI
89	Primary hAMSC	10% exosome-depleted-FBS, 48 h	-Differential centrifugation: 300×g for 10 minutes; 2000×g for 10 minutes; 10,000×g for 30 minutes; -Filtration: 0.22-μm filter; -Ultracentrifugation: 100,000g for 2h; Washed in PBS at 100,000×g for 2 h	-Protein concentration: BCA assay;-Morphology: TEM; -sEVs size distribution, concentration: DLS; -sEV markers: Flow cytometry (CD63, TSG101)	105.89±10.36 nm	Cup- and sphere-shaped	Positive for CD63 and TSG101
69	hBMMSCs (exposed to 100mT SMF and 50 μg/mL Fe_3_O_4_ NPs or naïve)	10% exosome-free FBS, 48h	-Differential centrifugation: 300×g for 10 minutes; 2000 ×g for 20 min; -Filtration: 0.22-μm filter; 10,000 ×g for 30 min; -Ultracentrifugation: 100,000g for 70min; Washed in PBS at 100,000×g for 70min.	-Protein concentration: BCA assay; -Morphology: TEM; -sEVs size distribution, concentration: NTA; -sEV markers: Western blot CD9, CD63, CD81 and TSG101 and calnexin	50-150 nm	Cup- or sphere-shaped	-Positive for CD9, CD63, CD81 and TSG101; -Negative for Calnexin
191	Peripheral blood (from DFU and non-diabetic subjects)	NA	-Differential centrifugation: 3000g for 15min; 10,000g for 30 min; 100,000g for 70 min; -Washing with PBS (3x): 100,000g for 70 min; -Filtration: 0.2µm filter; -Ultrafiltration: 4000×g	-Protein concentration: BCA assay; -Morphology: TEM; -Size and size: distribution DSL; -sEV markers: Western blot (TSG101, CD9); Flow cytometry (CD63, TSG101)	30-150 nm	Cup- or sphere-shaped	CD9 and TSG101 (both groups similar features, size, cd markers, shape)
107	Primary hADMSC	Serum-free medium, 24h	-Differential centrifugation: 4 °C, 300 g, 10min; 4 °C, 2000 g, 10 min; -Ultracentrifugation: 4 °C, 100,000 g; 70 min twice to purify	-Morphology: TEM; -EV Markers: Flow cytometry, CD63, CD81	100 nm	Spheroidal shaped	Positive for: CD63, CD81
72	hUC-MSCs (cell line)	10% exosome free serum	-Exosome extraction kit	-Protein concentration: BCA assay; -Morphology: TEM; -Size and size distribution: NTA; -EV markers: Western blot (CD63 and CD81)	30-150 nm (44%)	Saucer-like	Positive for CD63 and CD81
57	Primary hBMMSCs (preconditioned with 1µM-ATV for 48h)	Serum free culture medium, 48h	-Differential centrifugation: 300 g for 5 min; 2000 g for 20 min; -Filter: Filter (0.22 μm); -Ultracentrifugation: 100,000 g for 1.5 h (2x)	-Morphology: TEM; -Size distribution and concentration: NTA; -EV markers: Western blot: TSG101, Alix, CD81	80-120 nm	Spherical	Alix, TSG101, and CD81
64	hBMMSCs (transfected with miR-126 mimic)	Exosome free media, 48h	-Differential centrifugation: 300 g for 10 min; 2000 g for 30 minutes; 10 000 g for 30 minutes; -Ultracentrifugation: 100 000 g for 70 minutes (2x)	-Morphology: TEM; -Size distribution: NTA; -EVs Markers: Western blot: CD81,CD9, Alix	30-200 nm	Sphere- or cup-shaped	Positive for CD81, CD9, Alix
75	HUVECs	1 × Serum replacement solution, 48h	-Differential centrifugation: 300 g for 10 min; 2000×g for 10 min; 10,000×g for 30 min; -Ultra-filtration: 15 mL Amicon Ultra-15 Centrifugal Filters 4000rpm for 20mins; -Ultracentrifugation: 110,000×g for 70 min	-Morphology: TEM; -Size distribution and concentration: TRPS; -EV markers: Western blot: CD9, CD63, CD81 and HSP70	50-140 nm	Cup or spherical	Positive for CD9, CD63, CD81 and HSP70
99	Primary hUC-MSCs	2% exosome depleted FBS (120,000×g overnight), 24h	-Differential centrifugation: 300×g at 4 °C for 10 min; 16,500×g at 4 °C for 20 min; -Filtration: 0.22-μm filter; -Ultracentrifugation: 120,000×g at 4 °C for 90mins	-Protein concentration: BCA assay; -Morphology: TEM; -Size distribution: NTA; -EV markers: Western blot (CD9, CD63, Alix, TSG101, and HSP70)	20-200 nm (85%)	Cup-shaped	Positive for: CD9, CD63, Alix, TSG101, and HSP70
104	Primary h-hDPSCs, P-hDPSCs (matched pairs, 5 n)	Serum-free medium, 48h	-Differential centrifugation: 300×g for 10 min; 2000×g for 10 min; 10,000×g for 30 min; -Ultracentrifugation: 100,000×g for 70min (2x)	-Morphology: TEM; -Concentration and size: NTA; -EV markers: Western blot (ALIX, HSP70, CD9, and CD81)	30-200 nm	Cup-shaped	ALIX, HSP70, CD9, and CD81
43	Primary hADMSC	Serum free medium, 48h	-Differential centrifugation: 500g for 5 min; 3000g for 15 min; -Ultracentrifugation: 100,000g at 4°C for 1 hour; -Filtration: 0.22-mm filter; -Ultrafiltration: (pore size: NR)	-Protein concentration: BCA assay; -Morphology: TEM; -Size and size distribution: NTA; -EV markers: Western blot, CD63, TSG101, Alix.	(95%) 50-200nm	Circular	Positive for CD63, TSG101, and Alix
192	Primary hADMSC (transfected with NC or miR-19b inhibitor)	Serum free medium, overnight	Ultracentrifugation: 2000 g for 30 minutes; 100,000 g at 4°C for 60mins	-Protein concentration: BCA assay; -Morphology: TEM; -Size and size distribution: NTA; -EV markers: Western blot (CD63, HSP70)	100nm	Oval-shaped membrane vesicles	Positive for CD63, HSP70
49	Primary hUSCs	Serum free medium, 48h	-Differential Centrifugation: 300g for 10 min; 2000g for 10 min; -Filtering: 0.22 µm; -Ultra-centrifugation: 100,000×g for 70 min (2x)	-Morphology: TEM; -Size distribution and concentration: TRPS; -EV markers: Western blot: CD9, CD63, TSG101 and GM130	80-200 nm	Cup-shaped	Positive for CD9, CD63, TSG101 and negative for GM130
90	Primary, hADMSCs	Serum free medium, 48h	-Differential centrifugation: 800 g for 5 min; 2000 g for 10min; -Filtration: 0.1 mm pore; -Ultrafiltration and concentration: 100,000-MWCO; -Ultracentrifugation: 100,000 g for 1 h (2X)	-Morphology: TEM; -Size distribution and concentration: NTA; -EV markers: Western blot: CD63, HSP90, calnexin	Peak at 106 and 130 nm	Cup-shaped	Positive for CD63, HSP90, Negative for calnexin
79	Primary hUC-MSCs	10% exosome free FBS (100,000 g for 70 min)	15,000 rpm for half an hour; -Filtration: 0.22 μm filter; -Ultracentrifugation: 57,000 rpm for 60 minutes	-Morphology: TEM; -Size distribution and concentration: DLS; -EV markers: Western blot: TSG101, CD63 and CHAMP4, GAPDH	50 to 200nm	Round	Positive for TSG101, CD63 and CHAMP4, GAPDH

Summary of the methods used for separation and characterization of small extracellular vesicle used by the reviewed studies for treatment of wounds in animal models. **Abbreviations**: AFM: atomic force microscopy; calnexin: the endoplasmic reticulum protein; CDM: chemically defined medium; conf.: degree of confluency; D(+) markers: detected positive sEV markers; D (-) markers: Detected negative sEVs markers; DFcO: deferoxamine; DLS: dynamic light scattering; ELISA: enzyme‐linked immunosorbent assay; FBS: fetal bovine serum; Fibro-iPSCs: iPSCS derived from fibroblasts.; FNA: Flow NanoAnalyzer; FSEM: field‐emission scanning; GF: growth factors; GM130: cis-Golgi matrix protein, a negative exosomal marker; Grp94: glucose-regulated protein 94; (GM)130: the Golgi membrane marker cis-Golgi matrix protein; GPVI; glycoprotein VI; hADMSC: human adipose-derived mesenchymal stem/stromal cells; hADMSCs- Nrf2:human adipose derived mesenchymal stromal cell high expressed Nrf2; hAECs: human amniotic epithelial cells; hAMSC: human amnion mesenchymal stem cells; hAMSC-miR-135 OE: miR-135-overexpressing human amnion mesenchymal stem cell; hAMSC-miR-135 KD: miR-135-knocked down human amnion mesenchymal stem cell; HAPCS: hydroxyapatite/chitosan composite hydrogels; hBMMSCs: human bone marrow derived MSCs; hdMSC: human decidua-derived mesenchymal stem cells; hDPSCs: human dental pulp stem cells (DPSCs), p patient or h healthy; hENSC: human endometrial stem cell; hEPSCs: human epidermal stem cells; hFDMSCs: human fetal dermal mesenchymal stem cells; HFL1: human lung fibroblasts; hGMSCs; human gingival mesenchymal; hMenSCs: Human menstrual blood‐derived mesenchymal stem cells; hPECs: Human plasma endothelial cells; hPRP: human platelet rich plasma; hPMSCs: Human placenta mesenchymal stromal cells; HS-5: HPV-16 E6/E7 transformed human bone marrow mesenchymal stromal cell; hsaliva‑Exos: human saliva; HTC: high-throughput sequencing; hUCB-MSCs: human umbilical cord blood-derived MSCs; hUCB-EPCs: human umbilical cord blood-derived endothelial progenitor cells; hUCBP: human umbilical cord blood plasma; hUCBMNCs: human umbilical cord blood mononuclear cells; hUSCs: human urine stem cells; hUC-MSCs: human umbilical cord derived mesenchymal stem cells; iPSCs: induced pluripotent stem cells; LC-MS: Liquid Chromatography-Mass Spectrometry; LPS: lipopolysaccharide; mAPCs: mouse adipocyte progenitor cells; Macaque-Fibro-iPSCs: macaque-fibroblast-derived-induced pluripotent stem cells; MS: mass spectrometry, MWCO: molecular weight cut-off; NA: not applicable; ND: not detected; nm: nanometre; NR not reported; NS: not studied; Nrf2: nuclear factor-E2-related factor2; NTA: nanoparticle tracking analysis; PTHrP-2: Parathyroid hormone related peptide; P: passage; p-AT: porcine adipose tissue; PBS: phosphate-buffered saline; Pr: Protein; PVA: polyvinyl alcohol; rat-AT: rat adipose tissue; shRNA: small hairpin RNA vector; Con shRNA; scramble control shRNA; shDMBT1: lentivirus shRNAs with deleted in malignant brain tumors1; SEC: size exclusion chromatography; SEM: scanning electron microscopy; SMF: static magnetic field; USC-EVs: urine derived stem cell extracellular vesicles; SMSCs : synovium mesenchymal stem cells; SMSCs-126: synovium mesenchymal stem cells high expressed miR-126-3p; S-NC: immunoprecipitation-supernatant-negative control; sOMECs: sheets of oral mucosa epithelial cells; TEM: Transmission electron microscopy; TRPS: Tunable Resistive Pulse Sensing; TSG101; tumor susceptibility gene; UCG: ultracentrifugation; VCAM-1: vascular cell adhesion molecule 1; W: with; WB: Western blot; WJ: Wharton's jelly stem cells; Wistar Rat-ADMSC: adipose derived stem cells derived from Wistar rats, NS: not studied, NR:not reported.

**Table 2:** Animal study characteristics

Ref.	Animal model	Sample Size	Wound model	sEV intervention	Dose	Frequency	Route of administration	Vehicle	Follow-up period	Dose Response curve?	Comparator
52	B6.Lepr^db/db^ mice; Gender matched; 11 to 12 wks old; Genetically type 2 diabetic	5-7/G (3Gs)	Full-thickness excisional dorsal diabetic wounds; 6mm	Human GF stimulated fibrocyte derived-exos	5 or 50 µg exosome in 80 µl PBS	Once	SC injection and topical	PBS	21 days	No	200 μl of PBS
50	Rats; STZ induced diabetes	6/G (4Gs)	Full-thickness excisional ischemic leg diabetic wound; 10mm	LPS-hUC-MSC-exo	60 µg in 0.5 ml PBS	Once	Injected into wound edge	PBS	14 days	No	1) Untreated normal group; 2)Untreated diabetic group; 3)Diabetic + naïve hUC-MSC-exo
45	Adult female SD rats; 220±20 g	6/G (5Gs)	Full-thickness dorsal, deep second-degree burn wound; 16 mm	1) hUC-MSC-exo or,2) HFL1-exo	200 µg exosome (hUC-MSC-exo or HFL1-exo) in 200 µl PBS	Once	SC injection	PBS	14 days	No	1)1×10^6^ hUC-MSC in 200 µl PBS; 2) 1×10^6^ HFL1 in 200 µl PBS; 3) Untreated control
98	Adult male SD rat; 250-300 g	3Gs (NR)	Full-thickness excisional wound; 18 mm	hu-iPSC- MSCs-exo	160 µg hu-iPSC-MSC-exos	Once	SC injection	PBS	14 days	No	1) 160 µl PBS (SC injection); 2) MesenGro hMSC medium (SC injection)
73	ICR mice and nude mice (BALB/c-n); Adult, male	NR	Full-thickness dorsal skin wound (excisional); 1.5 cm	hUC-MSC-Exo	100 µg /ml of pbs mixed with hydrogel	Once	Injected	In hydrogel (1:1 ratio, (HydroMatrix, Sigma)	25 days	No	1) PBS; 2)HEK-293T-exosome (100 mg/ml); 3) UEFS
94	Adult Balb/c mice; Male; 6-8wks old	4Gs (NR)	Full-thickness excisional dorsal and inguinal skin wounds; 1.5 cm	hADMSC-exo	200 µg exosome in 200 µL PBS	Once	SC injection or I.V.	In PBS	21 days	No	1) untreated wound; 2) 200 µL PBS SC injection
60	SD rats; Male; 300-350g; STZ induced diabetes	NR	Full-thickness excisional diabetic dorsal skin wounds; 18mm	Human SMSCs-126-exo	Unclear	Once	Topical	HAPCS	14 days	No	1)Untreated; 2)HAPCS without exosome
62	Male, SD rats 300-350 g; STZ induced diabetes	NR	Full-thickness dorsal excisional wounds; 18mm	Human SMSCs-126-exo	Unclear (used 1.2ml in hydrogel preparation)	Once	Topical	Chitosan hydrogel	14 days	No	1)Chitosan hydrogel+PBS; 2) untreated control
93	SD rat, male, adult; 250-300 g; STZ induced diabetes	3Gs (6 wounds/G)	Full thickness excisional wound; 15mm	hUCB-EPC-exo	2×10^10^ or 1×10^11^exos in 200 μL PBS	Once	SC injection	PBS	14 days	No	200 µl PBS (SC injection)
105	Wistar rats, Male, 220	24 rats (2 Gs)	Excisional wound-splinting model; 5mm	hADMSC-EVs	Total EVs in gel prep: (1.9×10^8^ vesicles)	Applied daily	Topical with HEC	In a 1: 1 ratio with HEC gel (1%)	21 days	No	Plain HEC gel (1%)
80	SD rats, Male, 300-400g, 12 wks old, STZ induced diabetes	36 (4G, 9/G)	Full-thickness excisional diabetic dorsal skin wounds; 1.5cm	hPRP-Exo	NR (but explained how it was calculated)	Unclear	Topical	SAH	14 days	No	1) No treatment; 2) SAH only; 3) PRP+SAH
76	Male, SD rats; STZ and diet induced diabetes	24 (3Gs, 8/G)	A full-thickness excisional dorsal diabetic wound model; 10mm	hGMSC-exo	150 µg exo	Once	Topical	PBS+CS hydrogel	2 weeks	No	1)PBS+CS hydrogel; 2)PBS only
109	Balb/c mice; 6-8 weeks old	3Gs, 6/G	Full-thickness excisional dorsal wound; 1.5cm^2^ in	hADMSC-exo	200 μg in 200 µl PBS	Once	I.V.	PBS	21 days	No	1)PBS (200 µl); 2) CM-Exo (200µl)
91	SD rats, Male, 250-300g	6 rats (4 wounds/rat)	Full-thickness excisional dorsal wounds; 1 cm × 1 cm	hAECs-exo	25 μg/mL or 50 μg/mL or 100 μg/mL	Once	SC injection	PBS	21 days	Yes	100 µl PBS (SC injection)
84	C57BL/6 mice; Female, 8 months old, 25-30g, STZ induced diabetes	24 (8/G, 3Gs)	Full-thickness diabetic dorsal skin wound (excisional); 6mm	hUSCs^shDMBT1 #1^-Exos	200 μg hUSCs^shDMBT1 #1^-Exos in 100 μL PBS	Once	SC injection	PBS	12 days	No	(1) 100 μl PBS; (2) 200 μg hUSCs^Con shRNA^-Exos in 100 μl PBS
87	C57BL/6 mice; Male, 12 wks old, 26-30g	20 mice/2Gs	Full-thickness excisional dorsal skin wound; 12mm	hUCBP-exo	200 μg in 100 μL PBS	Once	SC injection	In PBS	8 days	No	2) 100 µL PBS SC injection
97	C57BLKS/J-Leprdb (db/db) mice; 9-wks old, Male, adult, Genetically diabetic, 41.0-45.5 g	NR	Full-thickness excisional wound-splinting model; Diabetic, 8mm	Human iPSCs-exo	4 µg in 20 µl PBS	Once	SC injection	In PBS	28 days	No	1)PBS,2) M-Exo
63	SD rats; Female, 150-200g, 4-6 weeks, STZ induced diabetes	NR	Round full-thickness excisional DFU at the dorsum of hind feet wounds; 5-mm	PB-EPC+hADMSCs-exo or PB-EPC+hADMSCs- Nrf2-exo	NR	Once	Injection	NR	15 days	No	1)PBS; 2)PB-EPCs
96	Inbred C57BL/6 mice; Male, 5-7 wks old, STZ induced diabetes	(9 Gs, 6/G at each time point)	Full‐thickness diabetic dorsal skin wound; 8mm	hMenSC-Exo	10 µg hMenSC-EVs in 100 µl of PBS	Once	I.D.	PBS	14 days	No	(1)PBS (control group, 100 µl); (2) hMenSCs (cell group, 1 × 10^6^ cells in 100μl PBS)
54	SD rats; Male, 250-300g, STZ induced diabetes	3 Gs, NR	Full-thickness diabetic dorsal skin wound (excisional); 20mm	DFO-hBMMSCs-exo	100 µg DFO- hBMMSCs-exo in100 µL PBS	Once	SC injection	PBS	14 days	No	1) 100 µg hBMMSCs Exo in 100 μl PBS; 2) 100 μl PBS
44	1) C57BL/6 wild-type; 2) Db/db mice, diabetic II, genetic model; 3) C57BL/6, diabetic I, STZ-induced; Male, 20-30 g, 8-10 weeks old	13 Gs (2 set of exp.)	Full-thickness excisional dorsal skin wounds; 6mm; Diabetic I,II, or nondiabetic	hUCBMNC-sEVs	A) sEV dosage exp: 0.4, 2 µg/wound; B) sEV+LTHAG exp: 2 µg/wound	Single dose or Bi-daily doses	1) Topical; 2) Injection	-sEV dosage exp: PBS; -sEV+LTHAG exp: HA hydrogel (Gel+sEVs+light)	10 days	No	A-sEV dosage exp: 1) PBS, 2)PDGF_bb_ 4µg/cm^2^; B-sEV+LTHAG exp: 1) Gel only+light, 2)Gel+sEVs only, 3) sEV+Gel on top+light
92	SD rats; STZ induced diabetes (type1)	NR (n = 3 in figures)	Full-thickness excisional dorsal diabetic wound; 1.5 cm	1) Low-concentration RAW 264.7-exos; 2) High-concentration RAW 264.7-exos; 3) High-concentration - RAW 264.7-exos + LPS	1) (100 µg/mL); 2) (1mg/mL); 3) 1 mg/mL exo+ LPS (10 µg/mL)	Once	SC injection	NR	14 days	No	1) 1ml PBS
42	Adult male macaques	4 animals	24 skin punch full-thickness dorsal excisional wounds/animal; 5-mm	Macque-Fibro-iPSCs-exo	50 μg exosomes	Once	Topical	NR	14 days	No	4.6 × 104 iPSCs (autologous or allogeneic) in 20µl
86	Adult male C57Bl/6 mice; 8-10 weeks	3Gs, 6/G	Full-thickness excisional wound-splinting model; 4mm	Mouse-Leukocyte-TBC1D3-exo	2x10^10^ EVs in 25 µl of PBS	Once	Topical	PBS	13 days	No	1) PBS; 2) EVs obtained from vector control cells
81	Adult male Wistar	12 (3Gs)	A full-thickness excisional wound model; 1.5 cm	Rat-ADMSC-exo	300 μl Alg-exo hydrogel	Once	Topical	Alg hydrogel	14 days	No	1) 300 μL Alg hydrogel; 2) Untreated control
82	Adult, SD rats weight 248 ± 26 g	10 (n = 9-10 wounds)	Full-thickness excisional dorsal wound model; 0.19 ± 0.03 cm^2^	sOMEC-cExo (Sheets of oral mucosa epithelial cells)	Exp1: 7.6 µg (day 0 and day 1); Exp2: 12.5 µg on day 0	2× vs 1×	Topical	Unclear	17 days	No	1-PBS (n = 6 wounds) 2-noncond* exo (from auto.serum supplemented medium)
53	Male, SD rats; Eight week old	4/G	Skin punch full-thickness dorsal excisional wounds; 8mm	4 types of hUCB-MSCs-EVs (10% O_2_, 40 U thrombin, 1 µg LPS, or 50 µM H_2_O_2_)	Exp1: EVs from (5×10^5^) of hUCB -MSCs Exp2: 20µg/10 µl of EVs	Once	NR	NR	8 days	No	1-Saline, 2-Naive EVs: Exp1: EVs from (5×10^5^) of hUCB-MSCs; Exp2: 20µg/10 µl of EVs
70	Male, ICR mice; 30gm, STZ induced diabetes	48 (3Gs, 12/G)	Full-thickness excisional dorsal diabetic wound; 8mm	Mouse- ADMSC-exo	10 µg of: 1-free exosomes or, 2-loaded in FHE-exo hydrogel	Once	Injection	FHE hydrogel	21 days	No	1-Saline; 2- FHE hydrogel alone
71	Male, ICR mice; 8 weeks old, STZ induced diabetes, Type 1 diabetes	48 (4Gs, 12/G)	Full-thickness round excisional dorsal diabetic wound; 10mm	1-Mouse-ADMSC-exo; 2-FEP+exo	Unclear	Once	Injection	FEP scaffold	21 days	No	1- Untreated 2- FEP only
108	BALB/c Mice; 8-10 wk old	5-7 /G, 2Gs	Full-thickness dorsal wound; 1 cm × 1 cm	FDMSC-exo	200 μg FDMSC-exosomes in 200 μl PBS	Once	SC injection	PBS	14 days	No	200 µl PBS (SC injection)
100	Female (BKS-Dock Leprem2Cd479, db/db); Genetically diabetic mice wounds	40 (5 at each timepoint)	Full thickness excisional dorsal diabetic wound; 16 mm	hdMSC -exo	5.22 × 10^11^ particles /ml in 100 μL PBS	At day 7, 14, 21, and 28	SC injection	PBS	28 days	No	100 µl PBS (SC injection)
102	Kunming, male mice; 9-12 wks, 26-30g	60 (4Gs, 15/G)	Full thickness excisional dorsal wound; 12 mm	hUC-MSC-EVs	200 µg in 100 µl PBS	Once	SC injection	PBS	8 days	No	1) 200 µl PBS (SC injection); 2) hUC-MSCs-EVs-inhibitor-NC; 3) hUC-MSCs-EVs-miR-27b-inhibitor
51	SD rats	3Gs, 3/G	Full thickness excisional dorsal wound; 15 mm	rat-sEV-AT or p-sEV-AT	600 µg in 100 µlPVA+100 µl PBS	Every week (3x)	Topical (dropping)	1:1 PVA+PBS	21 days	No	1:1 PVA+PBS (200 µl)
74	SD rats; 8 wk old, female, 200 g	30 (10/G, 3Gs)	Full thickness excisional dorsal wound; 15 mm	hEPSC-exos	200 µl (100 µg/ml of EPSC-Exos dissolved in PBS and hydrogel-1:1)	Every weeks (4x)	SC injection	Hydrogel (Hydro-matrix)	28 days	No	1)PBS (200 µl); 2) EGF+ hydrogel (200 µl)
66	SD rats, adult; 200 ± 50 g	25 (5/Gs)	Full thickness excisional dorsal wound; 1.5 cm× 1.5cm	1) Naïve hAMSC-exo; 2) hAMSC-exo-miR-135a OE; 3)hAMSC-exo-miR-135a KD	NR	Once	Topical (coated)	Type I collagen coat	15 days	No	1) Saline; 2) HEK 293T-exo
83	C57BL/6JRj mice; Female, 9 weeks	6/G	Full thickness excisional dorsal wound; 5 mm	HS-5 exos	15 µg or 1.5 × 10^11^ vesicles	3x (day 0, 2, 4)	I.D.	NR	5 days	No	1) SELL (1.5 × 1011 vesicles); 2) PBS
59	C57BL/6J mice; 8 weeks	6Gs	Full thickness excisional dorsal wound; 6mm	1) hBMMSC-TSG6-OE	100 µg/100 µl	Once	SC injection	NR	35 days	No	1) no wound ctrl; 2) wound+Saline; 3) wound hBMMSC exo-Lenti-Ctrl; 4) wound hBMMSC-Lenti-shTSG6-exo; 5) Wound hBMMSC-exo-Lenti-shCtrl
67	C57BL/6J mice; Male, 5 wks; 20.88 ± 1.94 g; STZ induced diabetes	3Gs, 12/G	Full thickness excisional foot diabetic wound (DFU); 10 mm	1) Mouse myeloid-MSC-exo vector; 2) MSC-exo OE H19	NR	Once	Injection	NR	13 days	No	1) Untreated control (12)
46	Wistar rats; 6 wks, male	4Gs, 9/G	Full skin thickness dorsal burn by diode laser	1) naïve hUC-MSCs-exo group in 100 μL PBS; 2) hUC-MSCs-exo + NPs; 3) hUC-MSCs-exo + NPs + MAG group	1) 100 μg Exos in 100 μL PBS; 2) 100 μg Exo + NPs in 100 μL PBS; 3) 100 μg Exo + NPs in 100 μL PBS	Once	I.V.	PBS	5 weeks	No	1) PBS group (100 μL PBS)
48	SD rats; Female, 6 weeks, 100 ± 5 g	15 /4Gs	Photoaged skin induced by ultraviolet B, wavelength 290-320 nm, dose of 7.8 J/cm^2^	hADSC-exo	25 g/mL in 100 μl PBS	Once	SC injection	PBS	28 days	No	(1) 100 μl PBS
47	SD rats; Male, 200-240 g, Deep second-degree skin burn induced rats	NR	Deep second-degree skin burns; 20 mm	hUC-MSC-ExAng-2 and hUC-MSC- Ex	1 mg in 200 µl	Once	SC injection	PBS	13 days	No	1) PBS 200 µl 1mg in 200 µl of: 2) hUC-MSC-Ex-GFP; 3) hUC-MSC-Ex-shCtr; 4) hUC-MSC-Ex-shAng-2
55	SD rats; 8 weeks old, Male, 250 g ± 10 g, Diabetic STZ induced model	54/3G	Full‐thickness dermal skin wound; 20-mm	hBMMSCs-melatonin-exo	NR	Once	SC injection	PBS	14 days	No	1)PBS (Control), 2) hBMMSCs-Exo
101	Kunming mice; 6-8 old weeks, Male, 18-22 g	25 (5/G)	Two full-thickness excisional skin wounds; 12-mm	hADMSC -sEVs	200 μg in 100ml of PBS	Once	SC injection	PBS	8 days	No	1)100uL PBS; 2) miR-486-5p antagomir; 3) antagomir NC (Concomitant injection of miR-486-5p antagomir or antagomir NC)
65	SD rats; Male, 150-200 g, 5-week-old, STZ induced diabetes	30/5Gs	Full‐thickness diabetic dorsal skin wound; 15 mm	hADMSCs-miR-21-5p-exo	NR	Three times	Topical and covered with gel	200 μl PBS	15 days	No	(1) Control; (2) Free miR-21; (3) Naïve Exos; (4) hADMSCs-miR-21-5p-exo-NC
88	C57BL/6 mice; 6-8 weeks old, Male	30/5Gs	Full-thickness excisional wound; 10-mm	hsaliva-exos	Saliva-Exos (100 μg in 100 μl PBS)	Once	SC injection	PBS	14 days	No	1) PBS (100 μl); 2) saliva (100 μl)
77	BALB/c mice; 8 weeks old, Male, 27 to 32g	15/3Gs	Full-thickness excisional wound, circular; 7-mm	Chitosan-glycerol-hENSC-exo	200 µl of corresponding hydrogels	Twice (day 3 and 7)	Topical	hydrogels	14 days	No	1) paraffin gauze; 2) Chitosan-glycerol
85	SPF Balb/c mice; 5-8 weeks old	9 (3G)	Full-thickness dorsal skin wound (excisional); 1 cm^2^ round	mAPCs-Exo	5 mg/kg	8x	I.P.	PBS	10 days	No	1) PBS; 2) Vim-/-APC-Exo
58	Wild-type (WT) neonatal and adult C57BL/6J mice; 5-7 g, 14 days old	3Gs, 9/G	Full-thickness excisional dorsal skin wounds; 1cm	1-mBMMSC-NS-exo; 2- mBMMSC-AS-exo	100 µg in 100µl PBS	Once	I.D.	PBS	14 days	No	100uL PBS
56	SD rats; 8 weeks, male, STZ induced diabetes	4Gs	Full-thickness skin wounds	PTHrP-2- HUVEC- exo	NR	Once	SC injection	PBS	14 days	No	Untreated HUVEC derived exosome
68	C57BL mice (male); STZ induced diabetes	18 (6/G, 3Gs)	Full-thickness excisional wound at dorsal leg; 4mm	1) Naïve ADMSC-exo 2) mmu_circ_0000250_ADMSC-exo	200µg exo in 100 µl of PBS	Once	SC injection	PBS	15 days	No	100 µl of PBS
61	Balb/c mice; 18-25 g	NM	Full-thickness midline excisional wound; 10-mm	Mouse melanoma B16F10 cell -exosomes 1-WT+PD-L1, 2-WT+IFN-γ	10 µg of exosome in 200µL of PF-127 hydrogel	Once daily from day 3 until day 7	Topical	Thermoresponsive PF-127 hydrogel	7 days	No	1) Negative group was treated with 20% PF-127 alone (Ctrl); 2) 20% PF-127 containing bFGF cytokine
78	C57BLKS-Leprdb mice; Male, 6-8 weeks, Congenital diabetes	60 (15/G, 4Gs)	Full thickness excisional dorsal, above the tail, diabetic wound; 7mm	1) hPMSC-exo in PBS; 2) hPMSC-exo in hydrogel	Total concentration 2 × 10^12^ mL^-1^ in hydrogel or 100 μL PBS	Once	Injection	MC-CS hydrogel or PBS	15 days	No	1) 100 μL PBS; 2) MC-CS hydrogel only
103	Mice, 4-week-old, STZ induced diabetes	48/G (3Gs)	Full thickness excisional midline dorsal; Size of 1 × 1 cm^2^	hPEC-EV	(100 μL of 50 μg/mL)	Every 3 days for a period of 14 days	Injection	PBS	14 days	No	1) 100 μL PBS every 3 days for14 days (injection); 2) 100 μL of 50 μg/mL S-NC every 3 days for 14 days (injection)
89	Male db/db mice (C57BL/KsJ); 8-12 weeks; leptin receptor-deficient diabetes	40 mice (n = 3 in figure)	Two full thickness dorsal wounds; 8mm	1) DMSO + hAMSC-exos; 2) LY294002 + hAMSC-exos	1) 10%DMSO + 200 µl hAMSC-exos (1000 µg/ml); 2) LY294002 (2.5 mg/kg) + 200 µl hAMSC-exos (1000 µg/ml)	Once	SC injection	PBS	18 days	No	1) 10% DMSO + 200 µl PBS
69	SD rats, Male, 300-400 g, Six-week-old	24 rats (3Gs, 8/G)	A full-thickness excisional dorsal skin wound; 20 ×20 mm	1) hBMMSC-exos; 2) Fe_3_O_4_ NPs-SMF-hBMMSC-exos	100 μg in 100 μL PBS	Once	SC injection	PBS	14 days	No	100 μL PBS
191	C57BL/6J mice; Male, 6 weeks, 20-30 g	5G, 6/G	Full-thickness dorsal skin wound (excisional); 10 mm	1) DFU-peripheral blood-exo; 2) NDFU- peripheral blood-exo; 3) DFU-Exos- AntagomiR-15a-3p	1,2) 200 µg exos in 100 µl PBS; 3) 2OD AntagomiR+ 200 µg exos	6 times	SC injection	PBS	14 days	No	(1) 100 μl PBS; (2) AntagomiR-15a-3p
107	BALb/c mice; Adult female, 5 weeks, 170-200 g	NR	Full-thickness dorsal skin wound (excisional, square); 1 cm^2^	hADMSCs-exos	NR	Once	SC injection	PBS	14 days	No	(1) PBS; (2) hADMSCs (1x10^7^)
72	SD rats; Male, 210±25 g, 10 weeks, STZ induced diabetes	24 (4G)	Two symmetrical; Full-thickness diabetic dorsal skin wound (excisional); 10 mm	1)hUCMSC-exo in PF-127 Hydrogel; 2) hUCMSC-exo in PBS	100 µg Exos in 100µl PF-127 or PBS	Every 3 days	Injected topically	PBS	14 days	No	(1) 100µl PF-127 only; (2) 100µl PBS
57	SD rats, Male, 250 g ± 20 g, 8 weeks, STZ induced diabetes	NR	Circular full-thickness diabetic dorsal skin wound (excisional); 2 cm	1)ATV-hBMMSC- exo; 2)hBMMSC- exo	NR	Once	Injection	PBS	14 days	No	1) PBS
64	C57BL/6 mice; 8-week-old, 20-25 g	24 (3Gs, 8/G)	Full thickness dorsal excisional skin wound	hBMMSC-miR-126-exo	200 μg Exo in 100 μl PBS	5x (0, 3, 6, 9, 12 days)	SC injection	PBS	14 days	No	1) 100 μl PBS; 2) 200 μg Exo-NC in 100 μl PBS
75	SD rats; 280-320 g, Male	18 (6/G)	Full-thickness dorsal skin wound; 10mm × 10 mm	HUVEC-exos	10^8^ particles/mL of GelMA	Once	Topical	In GelMA hydrogel	14 days	No	1) Control group (pressure dressing, no treatment); 2) GelMA hydrogel only
99	Balb/C mice, Male, 20-25 g	60 (6Gs, 10/G)	Full thickness dorsal excisional wound;0.8 cm × 0.8 cm	hUC-MSC-exos	100 μg 100 μl PBS	Once	SC injection	PBS	14 days	No	1) hUC-MSCs (1x10^6^ in 100 μl PBS); 2) PBS; 3) hUC-MSCs-CM (100 μl); 4) hUC-MSCs -dp-Ex (100 μl); 5) Sham (no treatment)
104	C57BL/6 mice; 8 weeks, 20-25 g	30 (10/G, 3Gs)	Full-thickness dorsal excisional skin wound	1) h-hDPSCs-EVs; 2)P-hDPSCs-EVs	200 μg in 100 μl PBS	Once	SC injection	PBS	14 days	No	1) 100 μL of PBS only
43	New Zealand Rabbit; Female, 2.5-3.0 kg	16 (8/G)	Hypertrophic scar model excisional; 8-mm-wound on the ventral side of both ears	1) hADMSC-EVs;2) EV-free medium	1) Unclear (0.1 ml EVs in PBS)	4 times (on day 0, 7, 14, 21)	Injection (base and edge of wound)	PBS	28 days	No	1) 0.1 mL of PBS
192	Balb/C mice, 20-25 g	15 (3Gs, 5/G)	Full-thickness dorsal excisional skin wound; 1cm in	1)hADMSC-exos-NC; 2) miR-19b inhibitor-hADMSC-exos-	100 µg exosomes in 100ml PBS	Once	SC injection	PBS	8 days	No	1)100 μL PBS
49	C57BL/6 mice; male, 6 weeks, Induced skin aging model (1 g/kg d-galactose for 2 months)	6/G (from results)	Skin pressure ulcers, 12mm	1) hUSC-EVs only; 2) hUSC-EVs-HAAM	NR	Once	Topical	HAAM/PBS	21 days	No	1) HAAM only (aged mice); 2) PBS (aged mice); 3) PBS (young mice)
90	Female C57BL/6 mice; 6 weeks old	3G (5/G)	Full-thickness dorsal excisional skin wound; 6 mm	1)S-MSC-exosomes; 2)Naïve-MSC-exosome	100 μg exo in 100 μl PBS	Once	SC injection	PBS	14 days	No	1) 100 μl PBS
79	SD rats; Female, 180-200 g, STZ induced diabetes	6, 2Gs	Circular full-thickness dorsal excisional skin wound; 15 mm	1)hADMSC-exo-PVA-Alg nanohydrogel	Unclear (300μL)	Once/day	Injection		18 days	No	1) hADMSC-exo only, 300 μL; 2) PVA-Alg Nanohydrogel only

Summary of the experimental design parameters of small extracellular vesicle intervention for treatment of wounds in animal models. **Abbreviations:** Alg hydrogel: alginate hydrogel; CS hydrogel: chitosan/silk hydrogel; CM-Exo: exosome-free conditioned medium; CM: conditioned medium; DFU: diabetic food ulcer; EV: extracellular vesicle; Exos: exosomes; EGF: epidermal growth factor; FDMSCs: Fetal dermal mesenchymal stem cells; FEP: scaffold of Pluronic F127 (F127) polyethylenimine (PEI) and aldehyde pullulan (APu); FHE hydrogel: Pluronic F127 (F127)+oxidative hyaluronic acid(OHA)+EPL; Fibro-iPSCs: iPSCS derived from fibroblasts; G: groups; GelMA: gelatin methacryloyl hydrogel; GF: Growth factor; HAAM: human acellular amniotic membrane; hADMSCs- Nrf2:human adipose derived mesenchymal stromal cell high expressed Nrf2; hADMSC: Human adipose-derived mesenchymal stem/stromal cells; hAECs: human amniotic epithelial cells; HAPCS: hydroxyapatite/chitosan composite hydrogels; hAMSC: human amnion mesenchymal stem cells; hAMSC-exo-miR-135 OE: miR-135-overexpressing human mesenchymal stem cell exosome; hAMSC-exo-miR-135 KD: miR-135-knocked down human mesenchymal stem cell exosome; hBMMSCs: human bone marrow derived mesenchymal stem cells; hdMSC-sEVs: human decidua-derived mesenchymal stem cells; HEC: Hydroxyethyl cellulose; HEK293T: human embryonic kidney 293 cells; hENSC: human endometrial stem cell; hEPSC: human epidermal stem cells; HFL1: human lung fibroblasts; hGMSCs; human gingival mesenchymal stem cells; h-hDPSCs: healthy teeth derived- human dental pulp stem cells; P-hDPSCs patient teeth derived- human dental pulp stem cells;hMenSC-exo: human menstrual blood‐derived mesenchymal stem cells exosomes; hPMSCs: Human placenta mesenchymal stromal cells; hPEC-EV: Human plasma endothelial cells-derived-extracellular vesicles;HS-5: HPV-16 E6/E7 transformed human bone marrow mesenchymal stromal cell; hUCB-EPC-exo: human umbilical cord blood-derived endothelial progenitor cells derived exosomes; hUCBMNCs: human umbilical cord blood mononuclear cells; hUCB-MSCs: hUC-MSCs: human umbilical cord derived mesenchymal stem cells; hucMSC-dp-Ex: exosome depleted conditioned medium; iPSCs: induced pluripotent stem cells; hUCBP: human umbilical cord blood plasma; human umbilical cord blood-derived MSCs; hUC-MSCs-EVs-inhibitor-NC: hUC-MSCs transfected miR-27b-inhibitor negative control-EVs; iPSCs- MSCs: induced pluripotent stem cell-derived mesenchymal stem cells; I.P: Intraperitoneal injection; I.V.: intravenous; I/C: intervention group vs control group; I.D.: Intradermal injection; mths: months; LPS: lipopolysaccharide; sEV-LTHAG: Hyaluronic acid light-triggerable hydrogel with EVs; MAG: magnet guided; MC-CS hydrogel: self-healing methylcellulose-chitosan hydrogel; mAPCs: mouse adipocyte progenitor cells; mBMMSC-NS-Exo: mouse bone marrow stromal cell preconditioned with neonatal serum derived extracellular vesicles prior to sEV isolation; mBMMSC-AS-Exo: mouse bone marrow stromal cell preconditioned with adult serum derived extracellular vesicles prior to sEV isolation; Macaque-Fibro-iPSCs-exo: macaque-fibroblast-derived-induced pluripotent stem cells-exosomes; NA: not applicable; NR: not reported; Nrf2: nuclear factor-E2-related factor2; PTHrP-2: Parathyroid hormone related peptide; PB-EPCs: Peripheral blood derived endothelial progenitor cells; p-sEV-AT: porcine adipose tissue derived EVs; PBS: phosphate-buffered saline; PDGF_bb_: PRP: platelet-rich plasma; Prep: preparation; PVA: polyvinyl alcohol; rat-sEV-AT: rat adipose tissue derived EVs; SAH: sodium alginate hydrogel; SD: Sprague Dawley; sEVs: small extracellular vesicles; SELL: synthetic exosome-like liposomes; shRNA: small hairpin RNA vector; Con shRNA; scramble control shRNA; shDMBT1: cells with knocked down DMBT1; shDMBT1: lentivirus shRNAs with deleted in malignant brain tumors1; Lenti, lentivirus; Ctrl, control; OE: overexpression; sh-Ctrl, negative control; SMF-Fe3O4 NPs- hBMMSC-exos: human bone marrow MSCs exposed to Static Magnetic Field and Fe_3_O_4_ nanoparticles; SMSCs : synovium mesenchymal stem cells; SMSCs-126: synovium mesenchymal stem cells high expressed miR-126-3p; S-MSC-exosomes: exosomes isolated from H_2_O_2_ induced-senescent MSCs; S-NC: immunoprecipitation-supernatant-negative control; sOMECs-cExo: sheets of oral mucosa epithelial cells derived exosomes from conditioned media; SC injection: subcutaneous injection; UEFS, UC-MSC: umbilical cord derived mesenchymal stem cells; umbilical cord-derived mesenchymal stem cell exosome-free supernatant; hUSC-EVs: urine derived stem cell extracellular vesicles; Wks: weeks; Xeno*: xenogenic.

**Table 3:** Safety and efficacy findings of the selected studies

Ref.	sEV type	Primary outcome measures	Main results	Secondary outcome measures	Main results	Conclusion
52	Human GF stimulated-Fibrocyte derived Exos	1-Wound healing rate; 2-Protein expression of re-epithelialization markers	1-Sig* dose-dependent acceleration of wound closure; 2-Sig* dose-dependent rise in COL1, α-SMA, and CK14 (day 14)	1-Adverse effect; 2-Angiogenesis	NS/NR; Sig* dose-dependent rise in MECA32 (day 7)	Exosome derived from fibrocyte may accelerate diabetic wound healing in a dose dependent manner.
50	LPS-hUC-MSC-exo	1- Wound closure rate	Wound closure rate was enhanced with LPS-hUCMSC-exo treatment.	1-Adverse effect; 2-Angiogenesis; 3-Inflammation	1-NR/NS; 2-More capillaries in LPS-hUC-MSC-exo group; 3-Reduced inflammatory cell infiltration and higher M2 than M1 at day 3. Increased TLR4, p-P65, pSTAT3 and p-AKT.	LPS-hUCMSC-exo can promote wound healing by modulating inflammation in diabetes rat model.
45	hUC-MSC-exos or HFL1-exo	1-Cell proliferation at burn wound -PCNA; 2- Re-epithelialization of burn wound - IF, WB: Cytokeratin19; 3- Collagen deposition: ColI: ColIII ratio	1) hUC-MSC-exo and hUC-MSC treated wounds had a similar sig* increase in cell numbers and PCNA compared with HFL1-exo, HFL1 and untreated control; 2) hUC-MSC-exo and hUC-MSC treated wounds similarly showed highest CK19 (wk1) and complete re-epithelialization (day 14); 3) hUC-MSC-exo and hUC-MSC showed sig* highest ColI: ColIII ratio	1-Adverse effect	1-NR/NS	hUC-MSC-exos improved wound healing and cell proliferation in second degree skin burn model in rats.
98	hu-iPSCs- MSC-exos	1-Wound size reduction rate; 2- %Re-epithelialization and scar width (mm); 3-Collagen maturity MT	1- hiPSCs-MSCs-exo treated wounds had a sig* accelerated WH at day 4, 7, 14; 2- hiPSCs-MSCs-exo treated wounds had a sig* higher degree of re-epithelialization, sebaceous glands and hair follicle formation with thinner scars at day14 compared to controls; 3- hiPSCs-MSCs-exo treated wounds had sig* highest collagen deposits at day14 with no loss of periodicity.	1-Adverse effect; 2-Angiogenesis; -IF: CD31 and α-SMA	1-NR/NS; 2- hiPSCs-MSCs-exo treated wounds had the greatest number of newly formed and mature blood vessel and the highest expression of α-SMA, CD31.	hiPSCs-MSCs-exos show potential in promoting angiogenesis and WH in rat model.
73	hUCMSC-Exo+hydrogel	1-Wound diameter; 2-Scar length; 3-IHC: α-SMA	1- hUCMSC-Exo+hydrogel showed a sig* reduction in wound diameter at day14 compared to controls (HEK-293T-exo, UEFS). Wound closed at day 25. 2- hUCMSC-Exo+hydrogel showed a sig* reduction in scar length at day 14 compared to controls; 3- Strongly reduced α-SMA in the hUCMSC-Exo+hydrogel group at day 25.	1-Adverse effect	1-NS/NR	hUCMSC-Exo can induce wound healing and also decrease scarring and myofibroblast development
94	hADMSC-Exo	1-Wound closure rate; 2-Collagen deposition and maturation: -MT, IHC (ColI,III), qRT-PCR	1-Wound closure of treatment was enhanced with hADMSC-Exo, relative to untreated and PBS control. -I.V. administration caused sig *superior closure rate compared with S.I (90% closure by day 21); 2- hADMSC-Exo sig* upregulated collagen I, III (highest level was at day 7) followed by gradual decrease; Collagen maturation was detected at late stage at day 14 and 21.	1-Adverse effects	1-NR/NS	- hADMSC-Exo promoted wound healing and collagen deposition and maturation. I.V administration of hADMSC-exo seems superior to S.I in healing process suggesting exosome homing to wound area.
60	HAP-CS-SMSCs-126-exos	1-Wound closure rate; 2-Neoepithelium length; 3-Collagen deposition and maturation: MT (H&E)	-Both HAP-CS-SMSCs-126-Exos and HAP-CS sig* promoted wound closure compared with untreated control; -HAP-CS-SMSCs-126-Exos had a superior effect over HAP-CS at day 7 and 14; - HAP-CS-SMSCs-126-Exos resulted in total wound closure at day 14; 2-Both HAP-CS-SMSCs-126-Exos and HAP-CS sig* promoted re-epithelialization compared with untreated control; -HAP-CS-SMSCs-126-Exos had a superior effect over HAP-CS; 3- HAP-CS-SMSCs-126-Exos greatly stimulated collagen deposition with improved alignment and maturity compared with the other groups.	1-Adverse effect; 2-Angiogenesis; -Microfil perfusion and micro-CT; -IHC: CD31 and α-SMA	1-HAP-CS-SMSCs-126-Exos did not show any adverse effects; 2-Both HAP-CS-SMSCs-126-Exos and HAP-CS sig* promoted vascularization compared with control. -HAP-CS-SMSCs-126-Exos had the greatest effect.	HAPCS-SMSCs-126-Exos has the potential to accelerate healing process of diabetic wounds.
62	Human SMSCs-126-exo	1-Wound closure rate; 2-Neoepithelium length; 3-Collagen deposition and maturation: MT	1- Human SMSCs-126-exo-Chitosan hydrogel sig* promoted wound closure compared with the Chitosan hydrogel and untreated group Exo-hydrogel > hydrogel > untreated Closed at day 14, while others not. 2-Human SMSCs-126-exo-Chitosan hydrogel had a sig* longer neoepithelium compared with hydrogel only and untreated controls. 3-Human SMSCs-126-exo-Chitosan hydrogel had sig* promoted collagen deposition, maturation, and alignment. The granulation tissue was the thickest.	1-Adverse effect; 2-Angiogenesis -Microfil perfusion and micro-CT -IHC: CD31 and α-SMA	1- No death or abnormality; 2- Human SMSCs-126-exo-Chitosan hydrogel promoted the greatest vessel density and vessel maturation compared with controls.	Chitosan hydrogel helped the controlled release of SMSC-126-Exos and improved wound healing in diabetes rat model.
93	hUCB-EPC-exos	1-Wound size reduction rate; 2-Re-epithelialization And scar formation (width); 3-Collagen maturity MT.	1- hUCB-EPC-exos treated wounds had a sig* accelerated WH at day14 compared to PBS; 1 × 10^11^ sig* > 2 × 10^10^ exosomes > PBS. 2- hUCB-EPC-exos treated wounds had a sig* higher degree of re-epithelialization and thinner scars at day14 compared to PBS; 1 × 10^11^ sig* > 2 × 10^10^ exosomes* > PBS. 3-larger amount of collagen deposits in hUCB-EPC-exos treated wounds 2 × 10^10^ exosomes had the greatest effect; 1 × 10^11^ sig* > 2 × 10^10^ exosomes > PBS.	1-Adverse effect; 2-Angiogenesis -Microfil perfusion and micro-CT -IF: CD31 and α-SMA	1-NR/NS; 2- hUCB-EPC-exos treated wounds had the greatest number of newly formed and mature blood vessel and the highest expression of α-SMA, CD31; 1 × 10^11^ sig* > 2 × 10^10^ exosomes* > PBS.	Exosome treatment promoted WH, and the increased dose had further enhanced exosome efficacy in promoting angiogenesis and WH in diabetes rat model.
105	hADMSC-EVs+HEC	1-Wound diameter	hADMSC-EVs+HEC sig* accelerated wound closure at day 7 and 14, but was equivalent to control at day 21	1-Adverse effect; 2-Angiogenesis	-NS/NR -NS	Topical application of a gel containing MSC EVs promoted wound healing in an animal model.
80	hPRP-exo	1-Rate of wound closure; 2- Neoepithelium formation; 3-Collagen deposition	1-hPRP-exo sig* accelerated wound closure (3, 7, 14 days), but showed no difference with PRP-only treated wounds at day 14; 2-Neo-epithelium was sig* longer in PRP-exo treated wounds compared to controls (including PRP only group) at day 14; 3- Massive deposition of woven collagen fibers in PRP-exo treated wounds compared to controls (including PRP only group).	1-Adverse effect; 2-Angiogenesis; -Microfil perfusion and micro-CT -IHC,IF: CD31, α-SMA and blood vessel denisty	1-No adverse effect detected; 2- Sig* high number of blood vessels in PRP-exo treated wounds compared to controls (including PRP only group); - Sig* high number of blood vessels and mature blood vessels in wounds treated with PRP-exo.	Exosomes secreted by PRP may mediate PRP-stimulated angiogenesis and accelerate diabetic wound healing.
76	hGMSC-exo	1-Wound closure rate; 2- Neo-epithelium length; 3-Collagen deposition and maturation: MT	1-hGMSC-exo-CS hydrogel sig* promoted wound closure compared with hydrogel and PBS group Exo-hydrogel > hydrogel > PBS Wound had almost closed by 2 weeks. 2-hGMSC-exo-CS hydrogel had sig* longer neoepithelium compared with hydrogel and PBS group Exo-hydrogel > hydrogel > PBS. 3-hGMSC-exo-CS hydrogel sig* promoted collagen deposition and maturation resembling normal skin at 2 weeks, compared with hydrogel and PBS group Exo-hydrogel > hydrogel > PBS.	1-Adverse effect; 2-Angiogenesis IHC: CD34 and blood vessel density.	1- NR/NS; 2- hGMSC-exo-CS hydrogel sig* promoted new blood vessels formation. -More aligned new vessels at week 1, and more oval/round at week 2.	The mixture of the exosomes and hydrogel may effectively enhance the skin wound healing of diabetes rat wounds.
109	hADMSC-exo	1-Scar width and scar depth (H&E); 2-Collagen deposition: -PSR staining -IHC IHC, WB, qRT-PCR: Col I, Col III. 3- Fibroblast differentiation to myofibroblast: -IHC,WB, qRT-PCR of TGF-β3:TGF-β1 -IF, WB, qRT-PCR: α-SMA.	1-Sig* Less scar width and depth in hADMSC-exo compared with PBS and the CM-Exo groups. 2- hADMSC-exo showed well-arranged collagen fibers comparable to native tissue. -Sig* Lower density of Collagen fibers compared to controls -Increased ratio of collagen III to collagen I. 3- hADMSC-exo raised the ratio of TGF-β3 to TGF-β1 at day 21 -α-SMA was sig* reduced at day 14 and 21 in hADMSC-exo compared with controls.	1-Adverse effect	1-NR/NS	hADMSC-exo stimulated ECM regeneration and reduced scar formation.
91	hAEC-exos	1-Wound size reduction rate; 2- %Re-epithelialization and scar formation; 3-Collagen deposition and organization: MT, IHC	1-Sig* dose-dependent acceleration of wound closure with hAEC-exos treatment (50 and 100 µg/ml) at day 14; 2-Remarkable dose -dependent re-epithelialization hAEC-exos treatment groups; -Complete re-epithelialization with less scar formation at day 14 with 100 µg/ml hAEC-exos. 3- Collagen fibers appear well organized in hAEC-exos treatment groups	1-Adverse effect	1-NR/NS	Exosome derived from amniotic epithelial cells may accelerate wound healing in a dose dependent manner.
84	USCs^shDMBT1 #1^-Exos	1-Rate of wound closure; 2-Re-epithelialization (H&E); 3-Scar width (H&E); 4-Collagen maturity (MT); 5_ Proliferation at wound area (IF of ki67)	1- USCs^Con shRNA-^Exos sig* accelerated wound closure, compared with PBS and shDMBT1 exo group (sig at day 14 only); 2- USCs^Con shRNA^-Exos sig* increased rate of re-epithelialization at day 12; 3- USCs ^Con shRNA-^Exos sig* reduced scar formation at day 12; 4- USCs^Con shRNA^-Exos - induced sig* higher collagen deposition; 5- USCs^Con shRNA^-Exos treated wound had sig* more ki67+ cells day 12.	1-Adverse effect; 2-Angiogenesis; IF: CD31 and blood vessel density.	1-NS/NR; 2- USCs^Con shRNA-^Exos significantly increased blood vessel formation (*p* < 0.05) day 12.	-USC-Exos is effective in promoting diabetic wound healing. -DMBT1 seems to mediate USC-Exos' wound healing and pro-angiogenic potential in diabetes mice.
87	hUCBP-exo	1-Wound closure rate; 2- Scar width; 3- %Re-epithelialization (H&E); 4-Collagen deposition and maturation: MT	1-hUCBP-exo sig* promoted wound closure. Wound has almost closed at day 8 post-wound. 2- hUCBP-exo greatly lowered scar formation. 3-Wound area re-epithelialization was sig* improved and longer compared with PBS control. Hair follicles and adipocytes appear to be restored. 4- Wavy collagen fibers were more abundant in hUCBP-exo treated wounds.	1-Adverse effect; 2-Angiogenesis IF: CD31 Vessel density	1-NR/NS. 2- hUCBP-exo sig* increased blood vessel formation (*p* < 0.05) indicated by increased CD31 expression (day 8)	Exosomes derived from UCB plasma could mediate the effect of plasma in stimulating skin cell proliferation, re-epithelialization angiogenesis and accelerating wound healing.
97	iPSCs-exo	1-Wound closure rate	1- iPSCs-exo enhanced wound closure but difference was not statistically sig* (wound closed after 19.0±3. 6), only at day 7 the difference was sig* -Wound closure in - iPSCs-exo was by regeneration of epithelium while it was by contraction in the PBS group.	1-Adverse effect; 2-Angiogenesis IF: CD31, α-SMA and blood vessel density	1-NR/NS. 2- Vascularization improved in the course of the healing process in all groups. iPSCs-exo treated wounds showed sig* better scores at day 7 only.	iPS-exos mildly stimulate wound healing in diabetes mice.
63	hADMSCs-exos or hADMSC-Nrf2-exos	1-% Wound closure; 2-Collagen deposition: MT.	1-Both hADMSCs-exos and hADMSC-Nrf2-exos sig* promoted wound closure compared with PB-EPC and PBS; - hADMSC-Nrf2-exos showed. 2- Sig* Reduced fibrosis by hADMSCs-exos and hADMSC-Nrf2-exos, with hADMSC-Nrf2-exos showing the greatest effect.	1-Adverse effect; 2-Angiogenesis IF: CD31 and blood vessel density.	1-NR/NS; 2- Both hADMSCs-exos and hADMSC-Nrf2-exos sig* promoted vessel formation, with hADMSC-Nrf2-exos showing the greatest effect.	Adipose derived stem cell-exosomes could potentially promote wound healing in DFU animal model. Overexpressing Nrf could further potentiate exosomes' therapeutic impact.
96	hMenSC-EVs	1-%Wound closure (day 0, 4, 7, 12, and 14). 2-Re-epithelialization (H&E). 3- Epithelial thickness (mm). 4-Size of scar tissue (width, depth, area). 5- Collagen deposition MT, qRT-PCR.	1- hMenSC-EVs sig* increased wound closure only at day 12, 14 (was not sig at day 4, 8). 2- hMenSC-EVs enhanced re-epithelialization. Difference was sig* with hMenSC group only at day 4, while effect was equivalent to hMenSC at days 7, 14. 3-Both EVs and hMenSCs increased epithelial thickness, but only hMenSCs showed a sig.effect compared with PBS. 4- EVs and hMenSCs similarly reduced scar size sig*. 5- hMenSC-EVs improved collagen deposition compared with hMenSCs and PBS (60%, 40%, 20%). Col1:Col3 ratio was higher in the EV group compared with controls at Day 7, it reduced at Day 14.	1-Adverse effect; 2-Angiogenesis -IHC: CD34 and microvessel density -qRT-PCR: *Vegfa* expression; 3-Inflammation Rela gene, iNOS and ARG detection	-NS/NR The mice's general health and behavioural changes were monitored. 2-hMenSC-EVs had sig* increased microvessel density and CD34 positive microvessels. -Vegfa was sig* upregulated with hMenSC-EVs. 3- *Rela* gene expression reduced slightly in exosome group at day 4 but increased at day 7. The ARG:iNOS ratio (M2:M1) was sig* higher in exo group.	hMenSC derived exosomes significantly promoted healing of diabetic wounds.
54	hBMMSCs-DFO-Exo	1-Rate of wound closure; 2-Scar width (H&E); 3-Collagen maturity (MT).	1-Wound closure was sig* accelerated with Exo and DFO-Exo at day 7 and 14 with DFO-Exo showing better effect. 2- Both Exo and DFO-Exo sig* lowered scar formation, but DFO-Exo showed a sig.effect compared with Exo. 3- Wavy collagen fibers more abundant in DFO-Exos-treated-wounds.	1-Adverse effect; 2-Angiogenesis: -microfil perfusion and micro-CT -IHC: CD31 and α-SMA.	-NS/NR. 2- sig* high number of blood vessels and mature blood vessels in wounds treated with DFO-Exos (*p <* 0.001) at day 14.	BMSCs preconditioned with DFO improve the proangiogenic capacity of exosomes and promote skin wound healing in diabetes mice.
44	hUCBMNC-sEVs	1-Wound healing rate; 2- Re-epithelialization (H&E); 3- IF: keratin 14, keratin 5 expression.	1-Wound healing was sig* enhanced when small doses of EVs (0.02 µg, total 0.4 µg/wound) applied bidaily for 10 days compared with single dose application. -Wound healing was sig* highest because of the controlled release of sEVs from LTHAG; 2-Epidermal regrowth was the highest in sEVs+ LTHAG+light; 3- Keratin 14 was highly expressed in cells of sEVs+ LTHAG+light, while keratin 5 expression was similar to control (gel+light only).	1-Adverse effect; 2-Angiogenesis IF: CD31; Vessel density.	1-NR; 2- sEVs+ LTHAG+light CD31 expression and vessel density was sig* the highest relative to all control groups.	Dosage regimen does influence the efficacy of sEVs. Small frequent doses showed better outcome than an equivalent single dose. Controlled release LTHAG showed superior effect on healing, regeneration, and new vessel formation in diabetic I, II, and nondiabetic wounds.
92	RAW 264.7-exo	1-Wound size reduction rate; 2-Collagen deposition and maturation: MT.	1-Wound closure rate was significantly higher in sEV groups (100 µg/ml and 1 mg/ml) compared with PBS group at day 7, 14, and 21. At day 7, wounds closed by 18%, 64%, 81% when treated with PBS, 100 µg sEVs, 1mg/ml sEVs respectively. Completely closed at day 14 (for EVs) but not for PBS group. 2-Significantly larger deposition of well-organized collagen fibres in sEV treated groups compared with PBS control at day 7 and 14.	1-Adverse effect; 2-Angiogenesis: -IHC: CD31; 3-Inflammation	1-NS. 2-Increased blood vessel density and CD31 expression in sEV treated groups within 7 days of treatment. 3-Lower infiltrating immune cell count (neutrophils and macrophages) and TNF-a and IL-6, in sEV treated groups compared with control.	Macrophage derived-sEVs promoted wound closure, collagen deposition and maturation, angiogenesis and suppressed inflammation in a dose dependent manner.
42	Macque-Fibro-iPSCs-exo	1-Wound closure rate; 2-Epithelial thickness (mm); 3-Collagen deposition: MT.	1-Both Auto-iPSCs-exo and Allo- iPSCs-exo sig* accelerated wound closure compared with EPC and PBS, - Auto-iPSCs-exo showed better efficacy. -No sig* difference between Auto- iPSCs and Auto-iPSCs-exo; and no sig* difference between Allo-iPSCs vs Allo- iPSCs-exo; 2- Auto-iPSCs-exo* > Allo- iPSCs-exo* > PBS Auto-iPSCs-exo showed the greatest effect in stimulating epithelial growth. 3- Auto-iPSCs-exo* > Allo- iPSCs-exo* > PBS Auto-iPSCs-exo showed the greatest effect in stimulating collagen deposition.	1-Adverse effect; 2-Angiogenesis IHC: CD34 and blood vessel density.	1-Auto and allo iPSCs, and their exosomes, did not trigger immune rejection; 2- Both Auto-iPSCs-exo and Allo- iPSCs-exo sig* promoted vessel formation. - Auto-iPSCs-exo showed the greatest effect. -Auto-iPSCs-exo* > Allo- iPSCs-exo* > PBS.	-Both allogenic and autologous iPC-derived exosomes promoted wound healing with no immune rejection. -Autologous-exo showed better performance.
86	Mouse-leukocyte-TBC1D3-EVs	1-% Wound closure	TBC1D3-evs failed to promote wound closure while control EVs sig* accelerated wound healing	1-Adverse effect	1-NR/NS	Transduction of source cells with TBC1D3, which is involved in vesicle trafficking, hampered the stimulatory effect of EVs in wound healing.
81	Rat-ADMSC-exo	1-% Wound closure; 2-Epithelial thickness (mm); 3-Collagen deposition: MT.	1- Alg-EXO hydrogel sig* reduced wound size compared with Alg-hydrogel (day 7, 14). 2- Alg-EXO hydrogel showed the highest epithelial thickness. 3-Both Alg-EXO hydrogel and Alg-hydrogel promoted collagen deposition compared with untreated control.	1-Adverse effect. 2-Angiogenesis IF: CD31 and blood vessel density.	1-NR/NS. 2-Alg-EXO hydrogel showed the highest CD31 expression and blood vessel density.	Loading alginate hydrogel with ADMSC-exo promoted wound healing considerably.
82	shOMEC-cExo	1) Wound size area; 2) Re-epithelialization; 3) Collagen (Picrosirius staining).	1-Sig difference in wound healing capacity compared to non-exo control (day 6 and 17). With one application of 12.5 g was less beneficial than 2 applications of 7.6 g. 2) Reduction of the granulation tissue. At the 17-day, the hypertrophic epithelium detected in all groups was decreased to a close to normal size layer. 3) Normal collagen localization and deposition.	1-Adverse effect	1-NR/NS	Oral mucosal sheet derived EVs significantly promoted wound healing. Lower doses but frequent treatment application appears to have better efficacy than a single higher dose.
53	hUCB-MSCs-EVs	1-Wound closure rate	1- Thrombin preconditioned EVs group had sig* higher rate of WH compared with the other groups (hypoxia, LPS, H2O2, saline naïve EVs).	1-Adverse effect; 2-Inflammation.	1-NR/NS. 2-Thrombin preconditioned EVs showed reduced levels of TNFα and IL6 compared with the other groups (hypoxia, LPS, H2O2, saline naïve EVs).	Thrombin preconditioning showed improvement in sEV efficacy in wound healing.
70	Mouse-ADMSC-exo	1- Wound closure rate; 2-Re-epithelialization -H&E -IHC cytokeratin. 3-Collagen deposition and maturation: -MT -IHC Col I, Col III.	1-ADMSC-exo +FHE hydrogel showed a sig* accelerated wound healing. ADMSC-exo +FHE hydrogel > Free exo > FHE hydrogel > Saline; 2-ADMSC-exo +FHE hydrogel showed the best outcome with visible skin appendages and cytokeratin at day 21. ADMSC-exo +FHE hydrogel > Free exo > FHE hydrogel > Saline; 3- ADMSC-exo +FHE hydrogel had the most sig* amount of well-organized collagen fibres and highest collagen I/III.	1-Adverse effect; 2-Angiogenesis IHC: α-SMA and blood vessel density	1-NR/NS; 2- ADMSC-exo +FHE hydrogel promoted the greatest vessel density and vessel maturation compared with controls	Incorporating ADMSC-exo with FHE hydrogel along with the pH-responsive release of exo had a synergistic effect in promoting wound healing in diabetic mice.
71	Mouse-ADMSC-exo	1-Wound closure rate; 2- Re-epithelialization H&E; 3-Collagen deposition: MT, Immunostaining ColI, Col III; 4-Cytokeratin and Ki67.	1- At day 3, FEP-exo and exo groups had a similar enhancing effect on WCR. -At day 7 FEP-exo had the highest effect; 2- Abundant and thickest granulation tissue with skin appendages in FEP-exo group FEP-exo > exo > FEP > control. 3- FEP-exo group showed sig* highest collagen deposition at days 7, 14. FEP-exo > exo > FEP > control; and increasing trend of ColI and ColIII expression at days 7, 14. 4- FEP-exo group showed sig* highest Cytokeratin and Ki67 expression.	1-Adverse effect; 2-Angiogenesis IF: α-SMA and blood vessel density.	1-NR/NS; 2- Had the greatest number of newly formed blood vessel and the highest expression of α-SMA.	Loading ADMSC-exo to FHE hydrogel along with the pH-responsive release of exosome improved exosome efficacy in promoting wound regeneration and angiogenesis in diabetic mice.
108	FDMSC-exos	1-Wound closure rate; 2- Re-epithelialization H&E -IHC: Cytokeratin19; 3-Cell proliferation markers: -IHC PCNA.	1- FDMSC-exo sig* accelerated WH at day 7 and 14; 2- FDMSC-exo group had more cells and collagen deposition as well as thicker epidermis and higher cytokeratin 19 expression compared to PBS control at day 7 and 14; 3- FDMSC-exo group showed higher expression of PCNA.	1-Adverse effect	1-NR/NS	FDMSC-exos can promote wound healing by stimulating re-epithelialization, protein deposition and cell proliferation.
100	hdMSC-sEVs	1-Wound size reduction; 2-Scar width; 3-Collagen deposition and organization MT; 4- IF: PCNA, CXCR4, α-SMA, and p21.	1-Sig* wound size reduction on day 14 and 21 with hdMSC-sEVs treatment. 2- Sig* narrowest scar width at day14, 21 in hdMSC-sEVs group. 3- More abundant well-organized collagen fibers in hdMSC-sEVs treatment group. 4-Remarkably higher PCNA, CXCR4, α-SMA, and lower p21 expression in hdMSC-sEVs treatment group.	1-Adverse effect	1-NR/NS	hdMSC-sEVs can promote WH by stimulating re-epithelialization, protein deposition and cell proliferation in diabetic mouse model.
102	1) hUC-MSCs-EVs or, 2) hUC-MSCs-EVs-inhibitor-NC or, 3) hUC-MSCs- EVs-miR-27b-inhibitor.	1-Wound size reduction rate; 2-Re-epithelializationand scar width; 3-Collagen deposition and organization MT.	1- hUC-MSCs-EVs sig* accelerated WH rate, while hUC-MSCs-EVs-miR-27b-inhibitor sig* reduced WH rate (at days 4, 6, 8); hUC-MSCs-EVs > hUC-MSCs-EVs-inhibitor-NC > hUC-MSCs-EVs-miR-27b-inhibitor > PBS. 2-hUC-MSCs-EVs group had sig* narrower scar width and more neoepithelium , while hUC-MSCs-EVs-miR-27b-inhibitor sig* wider scar and less neoepithelium (at day 8); hUC-MSCs-EVs > hUC-MSCs-EVs-inhibitor-NC > hUC-MSCs-EVs-miR-27b-inhibitor > PBS. 3- More abundant well-organized collagen fibers in hUC-MSCs-EVs and hUC-MSCs-EVs-inhibitor-NC treatment groups, in contrast to hUC-MSCs-EVs-inhibitor-NC. hUC-MSCs-EVs > hUC-MSCs-EVs-inhibitor-NC > hUC-MSCs-EVs-miR-27b-inhibitor > PBS.	1-Adverse effect	1-NR/NS	Suppression of miR-27b in hUC-MSCs-EVs impaired the pro-reparative impact of hUC-MSCs-EVs on WH, including re-epithelialization, and collagen fibre organization and deposition in animal wound model.
51	rat-sEV-AT or p-sEV-AT	1-Wound size reduction rate; 2-Re-epithelializationand granulation tissue thickness.	1- Both rat-sEV-AT and p-sEV-AT equivalently sig* promoted wound closure. Biggest difference with PBS-PVA control was at day 10 (60% vs 20% closure respectively). 2- Both rat-sEV-AT and p-sEV-AT equivalently sig* promoted re-epithelialization and enhanced the thickness and order of granulation tissue and supported hair follicle growth compared to control.	1-Adverse effect; 2- Angiogenesis.	1-NR/NS; 2-Detected capillaries growth.	Allogenic and xenogeneic EVs derived from adipose tissue exhibited similar efficiency in promoting wound healing in rat model, indicating EVs from different species might possess similar therapeutic properties.
74	hEPSC-exos	1-Wound size reduction rate and scar formation (width); 2-Scar and appendages formation; 3-RT-qPCR: TGF-β1, downstream genes, Smad2, α-SMA and collagen I, Ki67.	1- hEPSC-exos sig* promoted wound closure at day 7, 14 and closed at day 14 with the least scar width in contrast to EGF and control group. 2- hEPSC-exos sig* suppressed scar formation with less myofibroblast, collagen type I and III fibers, and more appendages formation than control and EGF. 3- sig* lower levels in hEPSC-exos treatment group at day 14.	1-Adverse effect; 2-AngiogenesisIHC: CD31.	1-NR/NS; 2- hEPSC-exos treatment group had sig* highest expression of CD31 indicating more blood vessels formation.	Exosomes derived from epidermal stem cells could remarkably accelerate wound closure stimulate angiogenesis and suppress scar formation through suppression of TGF-β1 and downstream genes.
66	1) Naïve hAMSC-exo. 2) hAMSC-exo-miR-135a OE. 3)hAMSC-exo-miR-135a KD.	1-Wound size reduction rate a; 2-Re-epithelialization H&E.	1- hAMSC-exo-miR-135a OE sig* promoted wound closure rate. hAMSC-exo-miR-135a OE > Naïve hAMSC-exo > hAMSC-exo-miR-135a KD. 2- hAMSC-exo-miR-135a OE treated wounds had a sig* higher degree of re-epithelialization, less inflammatory cells, and larger granulation area.	1-Adverse effect	1-NR/NS	miR-135a overexpression in exosomes derived from hAMSCs could potentiate exosomes' therapeutic effect and enhance WH in animal wound healing model.
83	HS-5-exo	1-Wound epithelial width and length; 2-Re-epithelialization H&E; 3-Collagen deposition and maturation Herovici staining; 4_ Proliferation at wound area (Ki-67 staining).	1-Wound length sig* reduced in HS-5 and SELL groups, but no sig* dif in wound width. 2-No sig* dif in re-epithelialization degree in the 3 groups. -Contraction was noted. -No sig* reduction in granulation tissue. 3-Sig* increase in collagen deposition and maturation in HS-5 and SELL groups. 4- A mild increase in ki67 expression in HS-5 only that was not sig.	1-Adverse effect; 2-Angiogenesis; -Blood vessel density, α-SMA and MECA32 staining.	1-NR/NS; 2-HS-5 exosomes and SELL similarly promoted blood vessels formation in the granulation tissue in terms of number and size as well as maturation.	-HS-5-exo and SELL exhibited a mild effect in promoting wound. -Equivalent influence of HS-5-exo and SELL in promoting collagen formation and wound length reduction implies that lipids may have a vital role in the wound healing properties of exosomes.
59	hBMMSC-TSG6-OE exo	1-Scar formation Assessment; 2-Collagen deposition MT; 3-RT-qPCR, TGFβ1, collagen I, collagen III, α-SMA, p-SMAD2^Ser467^ and p-SMAD3^S423/S425^	1-hBMMSC-TSG6-OE exo sig* reduced scar formation, recovered cell polarity, increased TSG6 expression compared to knocked down TSG-6 exo and other controls. 2- hBMMSC-TSG6-OE exo sig* lowered collagen deposition in scar (64.4%) compared to naïve hBMMSC-exo (47.3%). Neutralizing TSG-6 in exo reversed this effect and increased collagen deposition in the scar. 3- hBMMSC-TSG6-OE exo sig* lowered TGFβ1, collagen I, collagen III, α-SMA, p-SMAD2^Ser467^ and p-SMAD3^S423/S425^.	1-Adverse effect; 2- Inflammatory markers.	1-Degeneration, necrosis, and fibrosis were evaluated. 2-hBMMSC-TSG6-OE exo sig* lowered inflammatory markers: MCP-1, TNF-α, IL1β, IL6.	Transfection of hBMMSC-exos to overexpress TSG6 improved the pro-healing capacity of exosomes and reduced inflammation and scar formation in animal wound model.
67	1) MSC-exo vector; 2) MSC-exo OE H19 vs untreated control.	1-Wound closure %; 2-Re-epithelialization.	1- MSC-exo oe-H19 significantly accelerated wound healing rate. 2- Exo treatment produced significantly thicker granulation tissue, expression of collagen I (*p* < 0.01).	1-Adverse events; 2- Angiogenesis; 3-Inflammation.	1-TUNEL assay showed apoptosis was suppressed. 2- Sig increased VEGF, TGF-β1, α-SMA levels. 3- MSC-exo OE H19 suppressed IL-1β and TNF-α, and increased IL-10 expression.	
46	hUC-MSCs-exo vs hUC-MSCs-exo+Fe3O4 NPs. hUC-MSCs-exo+Fe3O4 NPs+ MAG.	1-Wound closure%; 2-Re-epithelialization%; 3- Wound edge length (mm); 4-Collagen deposition MT.	1-At week 5, The Exo and Exo + NP groups showed similar sig* accelerated closure rate, enhanced collagen deposition, re-epithelialization, reduced wound edge length. While Exo + NPs + MAG had the greatest sig* effect of all. - At week 5 CK19 expression was sig* greater in the Exo, Exo + NPs, and Exo + NPs + MAG than in the control.	1-Adverse effect; 2-Angiogenesis -IF: CD31 and α-SMA.	1-NR/NS; 2- At week 5, The Exo and Exo + NP groups showed greater average vessel density and number of mature vessels than the control. While Exo + NPs + MAG had the greatest effect of all.	Exosomes derived from Fe3O4 NP-loaded MSCs along with magnet guidance has enhanced homing, retention and efficacy in burn wounds.
48	hADSC-exo	1- Epidermal and Dermal thickness (H&E); 2- Nucleus Proliferation in Stratum Basale Cells of the Epidermis; 3- Type I Collagen, Type III Collagen, MMP-1, and MMP-3 mRNA in the Dermis (RT-PCR); 4- Protein Expression of Type I Collagen, Type III Collagen, MMP-1, and MMP-3 mRNA in the Dermis (WB).	1- hADSC-exo showed a reduced thickness of the epidermis and increased in the dermal thickness of the photoaged skin at day 7, 14, and 28. 2- hADSC-exo sig *reduced the nuclei in a proliferating state in the stratum basale. 3- hADSC-exo upregulated the relative gene expression of type I collagen mRNA and downregulate the expression of type III collagen, MMP-1, and MMP-3. 4- hADSC-exo upregulate the relative protein expression of type I collagen protein and downregulate the expression of type III collagen, MMP-1, and MMP-3.	1-Adverse effect	1-NR	hADSC derived exosomes treatment potentially improved photodamaged skin, promoted proliferation, restored epidermis and dermis thickness and improved collagen type I production.
47	hUC-MSC-ExAng-2	1- Wound healing rate; 2-Epidermis and appendages regeneration	1-hucMSC-Exo showed a sig * faster wound closure at 13 days compared to controls (EX-GFP, PBS). 2- hucMSC-Exo group had a better regenerated epidermis and a small number of appendages.	1-Adverse effect; 2-Angiogenesis CD31-specific staining IHC (qualitative).	1-NR; 2- hUC-MSC- ExAng-2-treated group exhibited stronger CD31- expression, and more blood vessels. -Knocked down Exo: hUC-MSC- Ex-shAng-2 had attenuated CD31 expression.	Overexpression of angiopoietin-2 in hucMSC-Exo enhanced angiogenesis, accelerated cutaneous wound healing, and epidermis regeneration in a rat model of deep second-degree burn injury. On the other hand, knockdown of angiopoietin-2 attenuated exosome therapeutic effects.
55	MT-hBMMSC- Exo	1- Wound closure rate; 2- neoepithelium length rate (H&E); 3-Collagen deposition-Masson staining, RT-PCR.	1- MT-Exo sig* reduced wound size compared to other groups at days 7, 14 (PBS and naïve exo). 2- MT-Exo sig* increased neoepithelium formation compared to others (days 7, 14). 3- MT-Exo sig* upregulated collagen-related genes Collagen I and III, and increased thickness (days 7, 14).	1- Adverse effect; 2-Angiogenesis: IHC: expression of CD31; 3-Neovascularization - Microfil perfusion; 4-Inflammation (air pouch model).	1-NR; 2-MT-Exo increased CD31/α-SMA expression 3-MT-Exo developed sig* higher number of new blood vessels. 4-MT-Exo reduced CCR7 positive cells, and increased CD206 cells (M2: M1).	hBMSCs derived MT-Exo potentially healed diabetic wound by promoting re-epithelialization, wound closure, angiogenesis and increased M2 to M1 polarization activating PTEN/AKT pathway *in vivo*.
101	hADMSC -EVs	1- Wound healing rate; 2-Collagen deposition-MT; 3-Re-epithelialization (H&E);	1-hASC-EVs reduced wound size rate was sig* higher than controls. 2- hADMSC -EVs formed more collagen fibre than control. 3- hADMSC -EVs promoted wound re-epithelialization.	1-Adverse effect; 2-Angiogenesis: IF:CD31 & α-SMA expression.	1-NR; 2-hADMSC -EVs injected skin stained more with CD31 & α-SMA.	hADMSC -EVs accelerated cutaneous wound healing and enhanced angiogenesis *in vivo* model.
65	miR-21-5p-exo	1-Wound closure rate; 2-Re-epithelialization (H&E); 3- Collagen deposition (Masson staining).	1- miR-21-5p-exo -treated diabetic wounds, the unclosed rate was 71, 21, and 8% on days 5, 10, and 15 and higher than control. 2- miR-21-5p-exo reached highest re-epithelialization percentage of 98.4% Compared to controls miR-21 (62.6%), naïve exo (75.4%), (NC) (74.3%) and control group (52.7%). 3- miR-21-5p-exo significantly increased collagen deposition.	1-Adverse effect; 2-Angiogenesis; -IF: CD31+ α-SMA+) and vessel density. 3- Inflammation.	1-NS/NR; 2- miR-21-5p-exo increased the density of blood vessels higher than controls. 3- At day 15, there were less inflammatory cells in miR-21-5p-exo than in controls.	ASC-exos with miR-21 accelerating diabetic wound healing by increasing re-epithelialization, collagen remodelling, angiogenesis, and vessel maturation *in vivo*.
88	hsaliva-Exos	1- Wound diameter; 2- Wound healing rate.	1- The saliva and saliva-Exos groups had a higher rate of wound healing than the control exo > saliva > pbs At days 3, 5, 7, 10, 14. 2- Saliva-Exos showed smaller scar width was smaller in the saliva-Exos group than in the control group Exo > saliva > pbs.	1- Adverse effect; 2-Angiogenesis: IF expression of CD31 and neovascularization.	1- NS/NR; 2-higher number of CD31-positive cells saliva-Exos sig *showed higher neovascularization at the wound.	Saliva exosome accelerated *in vivo* wound healing and angiogenesis.
77	Ch-glycerol-EXO	1- Wound healing rate; 2-Re-epithelialization (H&E); 3-Collagen deposition-Masson staining and appendages.	1- Ch-glycerol-EXO sig* reduced wound size than other group at day 7. Both hydrogels completely healed at day 14 but not NC. 2- Ch-glycerol-EXO sig* sig* increased epithelial thickness. 3- Ch-glycerol-EXO produced more collagen and skin appendages.	1-Adverse effect; 2-Angiogenesis: IF, IHC expression of CD31; -vessel density.	1-No signs of infection; 2- Ch-glycerol-EXO increased vessel density and increased expression of CD 31.	Ch-glycerol combined with exosomes potentiated wound healing and promoted tissue regeneration in *in vivo* model.
85	mAPCs, Vim-/-APC-Exo)	1- wound closure rate; 2- RNA isolation and qPCR analysis.	1- WT-APC-Exo recovered faster with reduced scar compared to Vim-/-APC-Exo and PBS groups. 2- WT-APC-Exos sig* increased TGFβ and collagen I compared to the control group or Vim-/-APC-Exos group.	1-Adverse effect; 2-Angiogenesis; 3-Inflammation.	1-NS/NR; 2-NS; 3- WT-APC-Exo sig* lowered inflammation and immune cell infiltration compared to Vim-/-APC-Exo and control. 4- WT-APC-Exos downregulated of pro-inflammatory cytokines IL-6 and TNF- α and suppressed IL-10. Vim-/- exosomes have no effect in these cytokine productions.	Exosomal vimentin shortens the healing time and reduces scar formation thus enhances wound healing.
58	MSCNS-Exo 2- MSCAS-Exo	1- Wound diameter; 2- Wound healing rate; 3-Collagen deposition-Masson staining; 4-Re-epithelialization (H&E).	1-NS-Exo educated MSCs (MSCNS-Exo) healed the fastest among the three groups. 2- MSCNS-Exo formed better epithelial and appendages. 3- Collagen deposition is higher in MSCNS-Exo compared to AS-Exo educated MSCs (MSCAS-Exo). 4-Increased Krt14. Less alpha sma that indicate myofibroblast and contraction of the wound.	1-Adverse effect; 2-Angiogenesis: IF; CD31 expression.	1-NR; No effect on body weight; 2-MSCNS-Exo showed better CD31 expression MSCAS-Exo group- not significant; *p < 0.05.	-Exosomes released by educated MSCs could promote angiogenesis. -Exosomes released by neonatal-exo-educated-MSCs showed better performance than MSC-exo educated with adult exo.
56	PTHrP-2-pre-treated HUVEC derived exosomes vs untreated HUVEC derived exosomes.	1-Wound closure %; 2-Collagen deposition MT.	1) Sig* improved wound closure rate in PTHrP-2-HUVEC-exo. 2) 1, 2, and 14 days post wounding PTHrP-2-HUVEC-Exos group showed greatly enhanced re-epithelialization, and collagen organization.	1-Adverse effects; 2-Angiogenesis IHC: CD31 IF: CD31/α-SMA Microfil perfusion.	1-NR/NS; 2- 14 days post wounding PTHrP-2-HUVEC-Exos group showed greatly enhanced angiogenesis.	Pretreatment of HUVEC with PTHrP-2 has improved exosome efficacy in promoting healing and angiogenesis in rat diabetic wound model.
68	1) Naïve ADMSC-exo; 2) mmu_circ_0000250_ ADMSC-exo.	1-Wound closure%	1- mmu_circ_0000250 exosome group had a sig* accelerated wound closure, wound closed at day 14.	1-Adverse effects; 2-Angiogenesis IF: CD31.	2- TUNEL staining showed that mmu_circ_000025 exosomes significantly reduced apoptosis of skin tissue relative to control. 2-mmu_circ_0000250 exosome treated group showed sig* enhanced neovascularization.	Exosomes with a high concentration of mmu_circ_0000250 showed improved therapeutic outcomes via enhancing wound closure, angiogenesis and reduced apoptosis and autophagy activation, in diabetic wound model.
61	Mouse melanoma B16F10 cell line derived exosomes. WT+IFN-γ and WT+PD-L1 With pf-127 hydrogel.	1-Wound closure %; 2-IHC: Ki67, α-SMA, vimentin.	1- WT+IFN-γ and WT+PD-L1 groups exosome group had a sig* accelerated, re-epithelialization and wound closure equivalent to the positive bFGF group. Wound almost closed at day 10 while negative control had a large scab. 2- PD-L1 group showed sig* better expression indicating better, proliferation, migration, and maturation.	1-Adverse effect; 2-Inflammation.	1-No abnormalities with spleen, lymph nodes and animal weight. 2-Less infiltrated immune cells at day 7, and reduced IL6, TNF-a, granzyme B levels in EV groups.	Exosomal PD-L1 improves tissue repair and regeneration.
78	2) hPMSCs exosomes in MC-CS hydrogel.	1-Wound reduction rate; 2-Re-epithelialization (H&E) and appendage formation; 3-Collagen deposition MT.	1- Exo-hydrogel group accelerated wound closure sig* at day15 compared with exosome only, hydrogel only and PBS groups. 2-At days 5, 10, and 15, Exo-hydrogel group performed sig* better in terms of tissue thickness compared to exosome only, hydrogel only and PBS groups. Appearance of hair follicles. 3-MC-CS-Exo hydrogel group had more well-organized collagen fibers.	1-Adverse effects; 2-Angiogenesis IF: CD31; α-SMA; -VEGF expression (WB).	1- (Apoptosis): Bcl-2 levels increased (day 10) while BAX levels decreased (days 5, 10) by EXO-hydrogel group, indicating inhibition of apoptosis. 2-Increase expression of α-SMA and CD 31 in EXO-hydrogel group. -VEGF sig* increased in day 10 and 15.	Loading exosomes into self-healing hydrogels improved their functionality by improved wound closure rate, re-epithelialization, collagen maturation and appendage formation, and reduced apoptosis.
103	hPEC-EV	1-Wound reduction rate; 2-Re-epithelialization; 3-Collagen deposition (MT).	1- hPEC-EVs showed a sig* difference in wound reduction rate at days 3, 7, 10, and 14, and wounds almost closed at day 14 in comparison to control that did not close. 2- Sig* improved epithelial thickness and epidermal maturity, better epithelial-mesenchymal transition in hPEC-EVs group compared to control. 3- Sig* more collagen deposition in hPEC-EVs group compared to control.	1-Adverse effects: apoptosis; 2-Angiogenesis IF: CD31, CD34; 3-Anti-inflammatory effect.	1-No adverse reaction -hPEC-EV inhibited premature senescence. 2-improved neovascularization with Sig* more CD34- and CD31-positive cells in hPEC-EVs group compared to control. 3- hPEC-EVs group enhanced percentage of type II macrophages (CD163+) to type I macrophages (CD86+).	hPEC-EVs enhanced wound closure, improved re-epithelialization with improved epithelial thickness, collagen deposition, angiogenesis, and anti-inflammation in diabetic mice model.
89	hAMSC-exos	1-Wound reduction; 2-Collagen deposition (MT) picrosirius red; 3-IHC: Col 1A1, COL3A1.	1- hAMSC-exos group had a sig* accelerated wound closure (days 8, 12, 14) and reduced wound size relative to the DMSO + PBS. LY294002 hAMSC-exos had sig *decreased wound closure rate. 2- hAMSC-exos group had an increased collagen deposition that was well-organized and resemble native tissue. LY294002+hAECs-Exos group was in between. 3- hAMSC-exos group had a sig* increase in COL 1A1 and COL 3A1 expression. This was not observed in LY294002+hAECs-Exos group.	1-Adverse effects; 2-Angiogenesis IHC: CD31.	1-NR/NS; 2- hAMSC-exos group had a sig* higher capillary density of the (56±5.10/hpf) relative to PBS group (23±3.74/hp, *p* < 0.001). LY294002 + hAMSC-exos (35.67±2.87/hpf) was lower.	-hAMSC-exos have shown to remarkably accelerate wound closure, collagen deposition, neovascularization in diabetic wounds. -Blocking PI3K-AKT-mTOR activity with LY294002 suppressed hAMSC-exos therapeutic benefits which highlight the importance of this pathway.
69	SMF-Fe3O4 NPs-hBMMSC-exos and naïve hBMMSC-exos	1-Wound reduction; 2-Re-epithelialization, scar width; 3-Collagen deposition and maturation MT	1- SMF-Fe_3_O_4_ NPs- hBMMSC-exos showed the greatest wound closure rate at days 4, 7, 10 and 14 after wound creation. 2- SMF-Fe3O4 NPs- hBMMSC-exos significantly enhanced re-epithelialization with lowest scar width, increased formation of appendages. 3- SMF-Fe3O4 NPs- hBMMSC-exos promoted largest collagen deposition at day 14.	1-Adverse effects; 2-Angiogenesis: IF, IHC CD31.	1-NR/NS; 2- Sig* enhanced neovascularization SMF-Fe_3_O_4_ NPs- hBMMSC-exos marked by increased CD31 expression.	Loading hBMMSC-exos with SMF-Fe3O4 nanoparticles potentiated their pro-regenerative efficacy of EVs.
191	DFU-exo	1-Rate of wound closure	1- Closure rate was significantly higher in DFU-ExosAntagomiR-15a-3p compared DFU Exos.	1-Adverse effects; 2-Angiogenesis: IHC; Assess the blood perfusion.	1-Unclear; 2-CD31 was lower in DFU-Exo treated wounds and enhanced in ExosAntagomiR-15a-3p.	Inhibition of miR-15a-3p in exosome isolated from DFU patients, improved wound healing and angiogenesis.
107	hADMSC-exos	1-Rate of wound closure	1- hADMSC-exos accelerated wound closure. Day 7: (cell = exos)* > pbs Day 14: all healed but PBS group had large scar tissue.	1-Adverse effect.	1-NS/NR.	miR-21 is highly expressed in AD-exos and can significantly accelerate the wound healing process and enhance the migration and proliferation.
72	hUCMSC-Exo in PF-127 hydrogel	1- Rate of wound closure; 2- Ki67; 3- Re-epithelialization (H&E).	1- hUCMSC-exos- PF-127 treatment resulted in a significantly accelerated wound closure rate at days 7, 14 (almost healed at day 14) compared with hydrogel only and PBS groups. 2- Ki67 highly expressed in hUCMSC-exos- PF-127 compared to exosome only, PF-127 only or PBS hUCMSC-exos- PF-127 > (exo = pf-127 = pbs). 3- Re-epithelialization was more pronounced in hUCMSC-exos- PF-127 group, with appendages formation.	1-Adverse effect; 2-Angiogenesis -IHC: CD31 -Vessel density.	1-NR/NS; 2) Increased expression of CD31, hUCMSC-exos- PF-127 and exo only group. - Significant increase in hUCMSC-exos- PF-127 group and exo only group. - Increased expression of VEGF and TGFβ-1.	PF-127 hUCMSC-exos improved wound healing rate, re-epithelialization, and skin appendage formation in diabetic wound model.
57	1)ATV-hBMMSC- Exo; 2) hBMMSC- Exo.	1-Rate of wound closure; 2-Wound length; 3-Re-epithelialization; 4-Collagen deposition (MT).	1- sig * accelerated wound closure was observed in the Exos groups (ATV and naïve);ATV-Exo > naïve Exo > PBS at day 3, 7, and 14. 2- sig* lower wound length in ATV-Exo compared to naïve exo or pbs group; ATV-Exo*> naïve Exo* > PBS. 3- Better re-epithelialization and neoepithelium length. 4- Well-organized and more deposition of collagen fibers in the ATV-Exos group.	1-Adverse effect; 2-Angiogenesis IF; CD31, α-SMA, Microfil perfusion assay; 3-Inflammation.	1-Did not affect renal or liver functions (creatinine, BUN, ALT, AST). No edema or sensitivity. 2-Exo treated groups had better vessel area and number ATV-Exo* > naïve Exo*> PBS, higher CD31, α-SMA (7 to day 14 were sig*). 3-At day 14, considerable infiltration of inflammatory cells was still noticed in control groups.	ATV preconditioning of hBMMSC enhanced exosomes regenerative capabilities in diabetes rats and facilitated wound closure, re-epithelialization and angiogenesis.
64	hBMMSC-miR-m126-Exo	1-Rate of wound closure; 2-Scar width (H&E).	1- hBMMSC-miR-m126-Exo accelerated wound closure, compared with Control group; miRNA-126 exo > naive Exo > PBS. 2- hBMMSC-Exo-miR-126 sig* reduced scar formation.	1-Adverse events; 2-Angiogenesis (IF).	1-NR; 2-hBMMSC-Exo-miR-126 group promoted CD34 & CD31expression.	Exo-miR-126 is a potential agent to promote angiogenesis, and wound healing.
75	HUVECs GeIMAExos	1-Rate of wound closure; 2-Re-epithelialization (H&E); 3-Collagen deposition (MT), WB (ColI, ColIII).	1- HUVECs GelMA-Exos sig* accelerated wound closure, compared with Control group, & GelMA group. 2- HUVECs-Exos loaded in GelMA could significantly enhance re-epithelialization. 3- At day 14, HUVECs GelMA-Exos sig* improved collagen organization and deposition. WB showed sig* higher Col I and Col III.	1-Adverse events; 2-Angiogenesis: IF, IHC: CD31.	1-NR; 2-Significant higher volume and density of newly formed blood vessel in GelMA-Exos group.	HUVECs GelMA Exos promoted re-epithelialization, collagen deposition and angiogenesis thus accelerated wound healing.
99	hUC-MSCs-Exos	1-Rate of wound closure; 2-Re-epithelialization (H&E); 3-Epidermal regeneration: CK10 (IHC); 4-Scar assessment (α-SMA).	1- hUC-MSCs-Exos sig* increased wound closure (days 7, 14). 2. hUC-MSC-Exo injection sig* enhanced re-epithelialization hUC-MSC-Exo and hUC-MSC had sig* effect (days 7, 14). 3. hUC-MSCs-Exos elevated CK10, indicating epidermal regeneration. 4. hUC-MSCs-Exos decreased α-SMA at day14 indicating reduced scarring.	1-Adverse effect; 2-Angiogenesis: IHC: CD31.	1-NR/NS; 2-Promoted new blood vessels formation in the wound area; -Sig* upregulated CD31.	hUC-MSC-Ex injection effectively promoted rapid wound closure, re-epithelialization, formation of blood vessels; and reduced fibrosis and scar formation.
104	1- h-hDPSC-EVs; 2- P-hDPSC-EVs	1-Rate of wound closure; 2-Scar width (H&E).	1- P-EVs accelerated cutaneous wound healing in mice; P-EVs* > H-EVs* > PBS. 2- P-EV-treated wounds had a lower level of scar formation; P-EVs* > H-EVs*> PBS.	1-Adverse effect; 2-Angiogenesis (IF) C31.	1-No signs of distress; 2-Increased VEGF and C31 in P-EV group indicating higher vessel formation; P-EVs* > H-EVs* > PBS.	P-EVs outperformed H-EVs in terms of promoting wound closure, re-epithelialization, vascularization, and scar reduction.
43	hADMSC-EVs	1-Scar height (H&E); 2-Collagen 1 and myofibroblast aggregation formation, α-SMA expression (WB, MT).	1- hADMSC-EVs significantly reduced hypertrophic scars and less SEI. hADMSC-EVs* > (EV-free medium = PBS) 2- hADMSC-EVs reduced myofibroblast aggregation and collagen I deposition. More well-organized collagen I.	1-Adverse effects	1-NR	- hADMSC-EVs suppressed hyperthropic scar formation by reducing collagen 1 and myofibroblast aggregation.
192	1- hADMSC-exos; 2- miR-19b inhibitor-hADMSC-exos-	1-Rate of wound closure; 2-Granulation.	1- Both hADMSC-exos and miR-19b inhibitor- hADMSC-exos showed accelerated wound healing but with reduced regenerative capability of the miR-19b- inhibitor exo. 2- Thicker granulation tissue in hADMSC-exos group and TGF-β1 expression.	1-Adverse effects; 2- Inflammation.	1-NR; 2-Less infiltration of inflammatory cells and decreased expression of the inflammatory factor CCL1.	Suppression of miR-19b in exosomes repressed the therapeutic benefits of the exosomes. hADMSC-exos accelerated wound healing and improved granulation.
49	1) hUSCs-EVs only; 2) hUSCs-EVs-HAAM.	1-Rate of wound closure; 2-Scar; 3-Collagen maturity (MT).	1- hUSCs-EVs-HAAM treated group showed a sig* accelerated wound closure compared to all groups, followed by hUSCs-EVs only group (day 3, 7, 14). 2- Sig* diminished scar formation compared with control young mice. 3-Improved collagen deposition.	1-Adverse effect; 2-Inflammation.	1-NR/NS; 2-Reduced expression of IL-1, IL-6, and MMP-13.	HAAM further enhanced regenerative effect of hUSCs-EVs and was able to ameliorate cellular senescence and enhance healing in aged mice.
90	1- S-MSC-exosomes; 2-Naïve-MSC-exosome.	1-Wound closure.	1- Naïve-MSC-exosome group had Sig* reduced wound size compared with PBS (day 3, 6, 9) and accelerated closure. Naïve > Senescent > PBS.	1-Adverse effect; 2- Angiogenesis IHC, CD31.	1-NS; 2-Positive expression in naïve, and S-MSC groups but not PBS group Naïve > Senescent > PBS.	EVs isolated from senescent cells had impaired functionality compared with naïve EVs, highlighting the importance of aging status of parent cells in source selection.
79	1- hADMSC-exo- PVA-Alg nanohydrogel.	1-Wound closure; 2-Collagen deposition MT.	1- Wound almost closed at day 18; Accelerated wound closure with exo-NH* > exo* > NH = negCTRL. 2- Improved collagen deposition, and organization.	1-Adverse effects; 2-Angiogenesis.	1-NS/NR; 2-Exo hydrogel group scored sig* higher CD31, αS-MA expression (Q) and consequently, the amount of mature blood vessels. exo-NH *> exo* > NH only and increased VEGF levels.	EVs encapsulation in PVA-Alg nanohydrogel outperformed EV only group in accelerating wound healing in diabetes rat model.

Summary of the outcomes of small extracellular vesicle intervention for treatment of wounds in animal models. **Abbreviations;** CM-Exo: exosome-free conditioned medium; FDMSCs: fetal dermal mesenchymal stem cells; FHE hydrogel: pluronic F127 (F127)+oxidative hyaluronic acid(OHA)+EPL; ECM: extracellular matrix; EGF: epidermal growth factor; H&E: haematoxylin and Eosin stain; hADMSC: human adipose-derived mesenchymal stem/stromal cells; hADMSCs- Nrf2:human adipose derived mesenchymal stromal cell high expressed Nrf2; hAECs: human amniotic epithelial cells; hAMSC: human amnion mesenchymal stem cells; hAMSC-exo-miR-135 OE: miR-135-overexpressing human mesenchymal stem cell exosome; hAMSC-exo-miR-135 KD: miR-135-knocked down human mesenchymal stem cell exosome; HAPCS: hydroxyapatite/chitosan composite hydrogels; HAP-CS-SMSCs-126-Exos: hydroxyapatite/chitosan composite hydrogels enclosed synovium mesenchymal stem cells high expressed miR-126-3p exosomes; hBMMSCs: human bone marrow derived mesenchymal stem cells; hdMSC-sEVs: human decidua-derived mesenchymal stem cells; HEC: hydroxyethyl cellulose; HFL1: human lung fibroblasts; hEPSC: human epidermal stem cells; hGMSCs; human gingival mesenchymal stem cells; h-hDPSC-EVs: healthy teeth derived- human dental pulp stem cells; P-hDPSC-EVs patient teeth derived- human dental pulp stem cells; hMenSCs: human menstrual blood‐derived mesenchymal stem cells; HS-5: HPV-16 E6/E7 transformed human bone marrow mesenchymal stromal cell; hPEC-EV: Human plasma endothelial cells-derived-extracellular vesicles; hPRP: human platelet rich plasma; hUCB-EPCs: human umbilical cord blood-derived endothelial progenitor cells; hUCBP: human umbilical cord blood plasma; hUCB-MSCs: human umbilical cord blood-derived MSCs; hUCBMNCs: human umbilical cord blood mononuclear cells; hUC-MSCs-EVs-inhibitor-NC: hUC-MSCs transfected miR-27b-inhibitor negative control-EVs; IF: immunofluorescence; I.V.: intravenous; iPSCs: induced pluripotent stem cells; LPS-hUC-MSC-exo: lps induced human umbilical cord derived MSCs; LTHAG: hyaluronic acid light-triggerable hydrogel; MC-CS hyrogel: self-healing methylcellulose-chitosan hydrogel; MECA32:mouse specific microvessel marker; MT:Masson's trichrome staining; microcomputed tomography; NH: nanohydrogel; NP: nanoparticle; Nrf2: nuclear factor-E2-related factor2; NS: not studied, NR: not reported; p-sEV-AT: porcine adipose tissue derived EVs; PTHrP-2: parathyroid hormone related peptide; PB-EPC: peripheral blood derived endothelial progenitor cells; PBS: phosphate buffered saline; PCR: polymerase chain reaction; PSR: picrosirius red staining; (Q): analysed quantitatively; rat-sEV-AT: rat adipose tissue derived EVs; SEI: S-MSC-exosomes: exosomes isolated from H_2_O_2_ induced-senescent MSCs; Scar elevation index; SELL: synthetic exosome-like liposomes; shOMECs-cExo: sheets of human oral mucosa epithelial cells derived exosomes from conditioned media; Sig*: significance; S-NC: immunoprecipitation-supernatant-negative; SMF-Fe3O4 NPs- hBMMSC-exos: human bone marrow MSCs exposed to Static Magnetic Field and Fe3O4 nanoparticles; S.I: subcutaneous injection; MT-hBMMSC-Exo: melatonin stimulated human bone marrow MSC-derived exosomes; UEFS: umbilical cord-derived mesenchymal stem cell exosome-free supernatant; USC-EVs: urine derived stem cell extracellular vesicles; VEGF a: vascular endothelial growth factor A; WCR: wound closure rate.

**Table 4:** Tracking of transplanted extracellular vesicles

Ref.	EV type	Dye	Category	Tracking mode	Route of delivery	Organ examined	Detection timepoints	Dose/frequency	Findings
94	hADMSC-exo	DiR	Lipophilic fluorescent dye	*In vivo*	I.V.	Skin/wound bed	Day 1, 3, 7, 14 and 21	200 µg, once	Detected as early as day1, peaking at day 7 and then gradually declining but still detectable until day 21
87	hUCBP-exo	PKH67	Lipophilic fluorescent dye	*In vivo*	S.C	Skin/wound area	3 h, 12 h, 24 h; day 2, 5 and 8	200µg, once	Perinuclear localization in skin cells at 3h post-injection. Progressive, time dependent drop in signal at 24h and day 2 respectively. No signal was observed on day5 and 8
44	hUCBMNC- sEVs	Cy7- DPPE Cy5.5 or-DPPE (evaluated by FRET)	Lipid conjugated, lipophilic fluorescent dye, FRET	*In vivo*	Topical in PBS or in hydrogel (with and without light activation)	Skin/wound area	1, 12, 24, 48, 72, 96, 120, 144, 168 h	2 µg in PBS or hydrogel, once	Within 24h, reduction in FRET was 50%, 20%, 30% in sEVs only, Gel+sEVs, light triggerable gel +sEVs groups respectively, indicating improved stability with light-triggered hydrogel formulation.
42	Macaque-Fibro-iPSCs-exo	PKH26	Lipophilic fluorescent dye	Ex-vivo	Topical in PBS	Skin/wound area	Day3 (in figure day 7)	50 µg, once	Intercellular localization of sEVs was detected at day 3. Uptake of the autologous sEVs was more efficient than the allogenic sEVs.
86	Mouse-Leukocyte-exo	PKH26	Lipophilic fluorescent dye	*In vivo*	Topical in PBS	Skin/wound area	4h	2×10^10^, once	Clustering of labeled-sEVs in wound bed 4h post application
82	sOMEC-cExo	PKH26	Lipophilic fluorescent dye	*In vivo* and *ex vivo*	Topical in PBS	Skin/wound area	*In vivo*: days 0-6 *Ex vivo*: day 6	20 µg, twice (day 0, 1)	*In vivo*: positive signals were detected from day 0 till day 4 and could not be detected on day 5 and 6 due to formation of scab and hair regrowth. *Ex vivo*: positive signal was detected at day 6 after the sacrifice of animals.

A summary of research findings on the labeling and tracking of transplanted sEVs in skin wounds. **Abbreviations:** hADMSC-exo: human adipose stem cell derived exosomes; hUCBMNCs-derived exosome: human umbilical cord blood mononuclear cells; hUCBP-exo: human umbilical cord blood plasma exosomes; sOMECs-cExo: sheets of oral mucosa epithelial cells derived exosomes from conditioned media. Macaque-Fibro-iPSCs-exo: macaque-fibroblast-derived-induced pluripotent stem cells-exosome; I.V: intravenous transfusion. S.C: subcutaneous injection, PBS: phosphate buffer saline.

## Abbreviations

EV: extracellular vesicle
sEV: small extracellular vesicle
MSC: mesenchymal stem/stromal cells
NTA: nanoparticle tracking analysis
UF: ultrafiltration
UC: ultracentrifugation
SYRCLE's ROB tool: The Systematic Review Centre for Laboratory Animal Experimentation risk of bias tool
